# Riparian buffers made of mature oil palms have inconsistent impacts on oil palm ecosystems

**DOI:** 10.1002/eap.2552

**Published:** 2022-03-29

**Authors:** Michael D. Pashkevich, Sarah H. Luke, Anak Agung Ketut Aryawan, Helen S. Waters, Jean‐Pierre Caliman, Nadine Dupérré, Mohammad Naim, Anton M. Potapov, Edgar C. Turner

**Affiliations:** ^1^ Insect Ecology Group, Department of Zoology University of Cambridge Cambridge UK; ^2^ Sinar Mas Agro Resources and Technology Research Institute (SMARTRI) Pekanbaru Indonesia; ^3^ School of Geosciences University of Edinburgh Edinburgh UK; ^4^ Zoologisches Museum University of Hamburg Hamburg Germany; ^5^ J.F. Blumenbach Institute of Zoology and Anthropology University of Goettingen Goettingen Germany

**Keywords:** arthropod, biodiversity, chronosequence, heterogeneity, oil palm, riparian buffer, spider, tropical agriculture

## Abstract

Expansion of oil palm has caused widespread declines in biodiversity and changes in ecosystem functioning across the tropics. A major driver of these changes is loss of habitat heterogeneity as forests are converted into oil palm plantations. Therefore, one strategy to help support biodiversity and functioning in oil palm is to increase habitat heterogeneity, for instance, by retaining forested buffers around rivers when new plantations are established, or maintaining buffers made of mature oil palms (“mature palm buffers”) when old plantations are replanted. While forested buffers are known to benefit oil palm systems, the impacts of mature palm buffers are less certain. In this study, we assessed the benefits of mature palm buffers, which were being passively restored (in this case, meaning that buffers were treated with no herbicides, pesticides, or fertilizers) by sampling environmental conditions and arthropods within buffers and in surrounding non‐buffer areas (i.e., areas that were 25 and 125 m from buffers, and receiving normal business‐as‐usual management) across an 8‐year chronosequence in industrial oil palm plantations (Sumatra, Indonesia). We ask (1) Do environmental conditions and biodiversity differ between buffer and non‐buffer areas? (2) Do buffers affect environmental conditions and biodiversity in adjacent non‐buffer areas (i.e., areas that were 25 m from buffers)? (3) Do buffers become more environmentally complex and biodiverse over time? We found that buffers can have environmental conditions (canopy openness, variation in openness, vegetation height, ground cover, and soil temperature) and levels of arthropod biodiversity (total arthropod abundance and spider abundance in the understory and spider species‐level community composition in all microhabitats) that are different from those in non‐buffer areas, but that these differences are inconsistent across the oil palm commercial life cycle. We also found that buffers might contribute to small increases in vegetation height and changes in ground cover in adjacent non‐buffer areas, but do not increase levels of arthropod biodiversity in these areas. Finally, we found that canopy openness, variation in openness, and ground cover, but no aspects of arthropod biodiversity, change within buffers over time. Collectively, our findings indicate that mature palm buffers that are being passively restored can have greater environmental complexity and higher levels of arthropod biodiversity than non‐buffer areas, particularly in comparison to recently replanted oil palm, but these benefits are not consistent across the crop commercial life cycle. If the goal of maintaining riparian buffers is to consistently increase habitat heterogeneity and improve biodiversity, an alternative to mature palm buffers or a move toward more active restoration of these areas is, therefore, probably required.

## INTRODUCTION

Agriculture is expanding rapidly across the tropics (Gibbs et al., 2010; Tilman et al., 2001). One of the crops showing among the largest expansions in recent years is oil palm (*Elaeis guineensis*), which is grown to produce palm oil: the most widely traded vegetable oil worldwide (>70 million metric tons traded in the 2019–2020 fiscal year; USDA, 2020). The majority of palm oil production occurs in Southeast Asia, where oil palm plantations are the dominant landscape in some regions (Qaim et al., 2020). Although expansion of oil palm plantations can bring socioeconomic benefits, such as improved food security (e.g., Edwards, 2019), the conversion of natural habitat to oil palm also leads to widespread declines in biodiversity (Drescher et al., 2016; Foster et al., 2011) and alters a range of ecosystem functions, such as predation and soil fertility (Barnes et al., 2017; Dislich et al., 2017).

The relatively low levels of biodiversity and reduced ecosystem functioning within oil palm plantations are driven, in part, by reduction of habitat heterogeneity that occurs as natural landscapes are converted to oil palm systems (e.g., Drescher et al., 2016; Luskin & Potts, 2011). Therefore, one strategy to support biodiversity and functioning in oil palm plantations is to increase habitat heterogeneity within the crop landscape (Foster et al., 2011; Luke et al., 2020a; Luskin & Potts, 2011; Sirami et al., 2019). In comparison to other crops, particularly annuals, such as rice and soybean, oil palm plantations are an ideal system in which to enhance habitat heterogeneity through changes in management practice (Beyer et al., 2020). Oil palm is a perennial tree crop with a long commercial life cycle (20–30 years, although palms can live for more than a century; Corley & Tinker, 2016), providing ample time for heterogeneous habitat to develop. The crop is also grown over vast swathes of land (Descals et al., 2020), across which large areas of heterogeneous habitat can be established. Additionally, oil palm growers are incentivized to maintain heterogeneous habitat, as such practices are often a requirement for sustainability certification schemes (such as the Roundtable on Sustainable Palm Oil; RSPO, 2018).

Increasing habitat heterogeneity in oil palm plantations can be achieved at various scales. For instance, at a local scale, structural complexity can be increased by applying empty oil palm fruit bunches (EFBs) to the bases of mature palms (Tao et al., 2018), by retaining epiphytes on palm trunks (Prescott et al., 2015), or by enhancing the structural complexity of understory vegetation (e.g., Darras et al., 2019; Hood et al., 2020a; Luke et al., 2019a, 2020a, 2020b; Spear et al., 2018). At a landscape‐scale, habitat heterogeneity within plantations can be increased by intercropping palms with other cash crops (Ashraf et al., 2018; Asmah et al., 2017; Yahya et al., 2017), retaining rainforest fragments within plantations at the time of establishment (Lucey et al., 2014; Lucey & Hill, 2012), establishing diverse tree islands within plantations (Teuscher et al., 2016; Zemp et al., 2019a, 2019b), and maintaining riparian buffers along plantation waterways (Luke et al., 2019b). Collectively, strategies to increase habitat heterogeneity in oil palm plantations have been demonstrated to benefit a wide variety of taxa, including spiders (Spear et al., 2018), insects (Ashraf et al., 2018; Ashton‐Butt et al., 2018; Hood et al., 2020a; Lucey et al., 2014; Lucey & Hill, 2012), and birds (Teuscher et al., 2016; Yahya et al., 2017).

Increasing landscape‐scale heterogeneity through maintaining or restoring riparian buffers has particularly high potential to bring a wide range of environmental benefits in oil palm agriculture. Buffers, also called riparian reserves, corridors, strips, margins, and zones, border plantation waterways and are managed less intensely than surrounding cultivated areas. They can provide terrestrial habitat, freshwater protection, and landscape connectivity (Luke et al., 2019b), but require relatively little land area (Bicknell, n.d.). Within oil palm systems, buffers are typically formed from either (1) remnant patches of rainforest that were retained during plantation establishment (hereafter, “forested buffers”) or (2) zones of mature oil palm that are managed less intensely than surrounding cultivated areas and are maintained when mature oil palm is replanted with young palms at the end of its commercial life cycle (hereafter, “mature palm buffers”). These mature palm buffers are often allowed to passively restore; an approach to restoration in which ecosystems recover on their own, or with minimal human intervention (Ghazoul & Chazdon, 2017), and which allows succession within buffers to occur.

Most research to date has focused on forested buffers and has shown that they provide multiple benefits to oil palm systems. For instance, forested buffers can mitigate soil erosion, improve stream water quality (Chellaiah & Yule, 2018; Luke et al., 2017a), and act as microclimate refugia in oil palm landscapes (Williamson et al., 2020). In comparison to surrounding oil palm areas, forested buffers support more species of ants (Gray et al., 2015), birds (Mitchell et al., 2018), adult dragonflies (Luke et al., 2017b), and dung beetles (Gray et al., 2014). Birds (Knowlton et al., 2017) and moths (Gray et al., 2019) can move through forested buffers to cross oil palm landscapes and, in some circumstances, invertebrates can move from forested buffers into adjacent oil palm plantations (Gray et al., 2016). In comparison to forested buffers, the impacts of mature palm buffers on oil palm systems are less known. Only two studies to date, occurring in industrial plantations in Sumatra (Indonesia), have focused on mature palm buffers, finding that they do little to support different environmental conditions or levels of ecosystem functioning or multifunctionality (Luke et al., 2020a; Woodham et al., 2019), in comparison to cultivated areas. It is unknown whether mature palm buffers have levels of biodiversity that are different from those in cultivated areas, or whether buffers affect environmental conditions and levels of biodiversity in adjacent cultivated areas (i.e., just outside the mature palm buffers). This knowledge gap exists despite mature palm buffers becoming increasingly widespread as plantations across Southeast Asia are replanted, and because maintaining mature palm buffers during replanting is a requirement for major sustainability certifications (Barclay et al., 2017; RSPO, 2018; Indonesian Sustainable Palm Oil, http://ispo-org.or.id).

Oil palm is a long‐lived crop with a 20–30 year commercial life cycle. The impacts of mature palm buffers on oil palm systems are expected to vary across this period, as conditions in the surrounding cultivated areas change. As cultivated areas age, they become cooler and more humid (Luskin & Potts, 2011; Pashkevich et al., 2021a) and, depending on management, trunk epiphytes can become more abundant, depth of leaf litter can increase, and soil quality and nutrient cycling can fluctuate over time (Hamilton et al., 2016; Luskin & Potts, 2011; Pauli et al., 2014). These changes in environmental conditions may cause cultivated areas to have different levels of biodiversity, relative to mature palm buffers. For instance, the species‐level composition of ground‐foraging ants was found to differ between young (4–7 years), mature (10–13 years), and old (15–26 years) oil palm plantations (Wang & Foster, 2016). In addition, we previously demonstrated that the order‐level community composition of arthropods, and species‐level community composition of spiders, changed as second‐generation oil palm plantations aged (Pashkevich et al., 2021a). Further, it is likely that conditions within mature palm buffers themselves will change over time as succession occurs although, to our knowledge, this has not yet been investigated.

This study investigated whether mature palm buffers that were being passively restored affected habitat heterogeneity and biodiversity within oil palm systems and, if so, whether the effects were consistent across the oil palm commercial life cycle. We focused our biodiversity surveys on arthropods, as they are abundant within oil palm plantations; facilitate important ecosystem processes such as waste management (Gray et al., 2016), pollination (Li et al., 2019), decomposition (Eycott et al., 2019), and pest control (Nurdiansyah et al., 2016); and affect other animals as both prey and predators (Barnes et al., 2014). We aimed to (1) quantify differences in environmental conditions and biodiversity between buffers and surrounding non‐buffer areas (i.e., areas that were 25 and 125 m from buffers), and determine whether differences were consistent across the oil palm commercial life cycle; (2) evaluate whether buffers affected environmental conditions and levels of biodiversity in adjacent non‐buffer areas (i.e., areas that were 25 m from buffers) across the oil palm commercial life cycle; and (3) assess whether buffers became more environmentally complex and biodiverse over time.

## METHODS

### Study design

Data were collected in industrial oil palm plantations in Riau, Sumatra, Indonesia (N0 55.559, E101 11.619) as part of the Biodiversity and Ecosystem Function in Tropical Agriculture (BEFTA) Programme (Luke et al., 2020a; Appendix S1: Figure S1). The plantations are owned and managed by PT Ivo Mas Tunggal (a subsidiary of Golden Agri Resources [GAR]), and run with technical input from Sinar Mas Agro Resources and Technology Research Institute (SMARTRI). The area is divided into seven estates, which are managed semi‐independently. The natural habitat in the region is lowland rainforest, but oil palm plantation is now the dominant land use type (Ramdani & Hino, 2013). Mean annual temperature and rainfall at SMARTRI is 26.8°C and 2350 mm, respectively, with the rainy season occurring between October and April (Tao et al., 2016).

SMARTRI is managed in accordance with guidelines from three independent certification bodies: the Roundtable on Sustainable Palm Oil (RSPO; http://rspo.org), Indonesian Sustainable Palm Oil (ISPO; http://ispo-org.or.id), and International Sustainability and Carbon Certification (ISCC; http://iscc-system.org). SMARTRI plantations were chartered before recommendations and laws were in place that required retaining forested buffers during plantation establishment, and therefore all buffer areas in SMARTRI are currently made of mature oil palms. These mature palm buffers occupy ~200 ha of land (although we did not consider topography when we calculated this estimate), which corresponds to 1.36% of all area in SMARTRI plantations. Following guidelines, buffers are 50 m wide and managed less intensely than surrounding cultivated areas (i.e., they are treated with no herbicides, pesticides, or fertilizers). Therefore, buffers are managed according to a passive restoration strategy, an approach to restoration that allows ecosystems to recover on their own or with little human intervention (Ghazoul & Chazdon, 2017) and with no enrichment planting, although bamboo is sometimes planted along riverbanks to reduce erosion. As in cultivated areas, palms within buffers are harvested by hand using scythe‐like tools (*egreks*). Buffers are maintained when old plantations are replanted, creating remnant strips of mature palms in a landscape of young palms (Appendix S1: Figure S2). SMARTRI began replanting first‐generation palms in 2010 following recommended replanting strategies, which represent how replanting is likely to occur, or has already occurred, across most industrial estates in Southeast Asia (Pashkevich et al., 2021a). Over time, this has resulted in a chronosequence of differently aged areas of oil palm (hereafter, Cohorts), which contain mature palm buffers. We previously demonstrated that these cohorts differ from each other environmentally, in their vegetation composition, canopy openness, and soil temperature, and in terms of their management, including application of herbicides, pesticides, and fertilizers (Pashkevich et al., 2021a).

**FIGURE 1:**
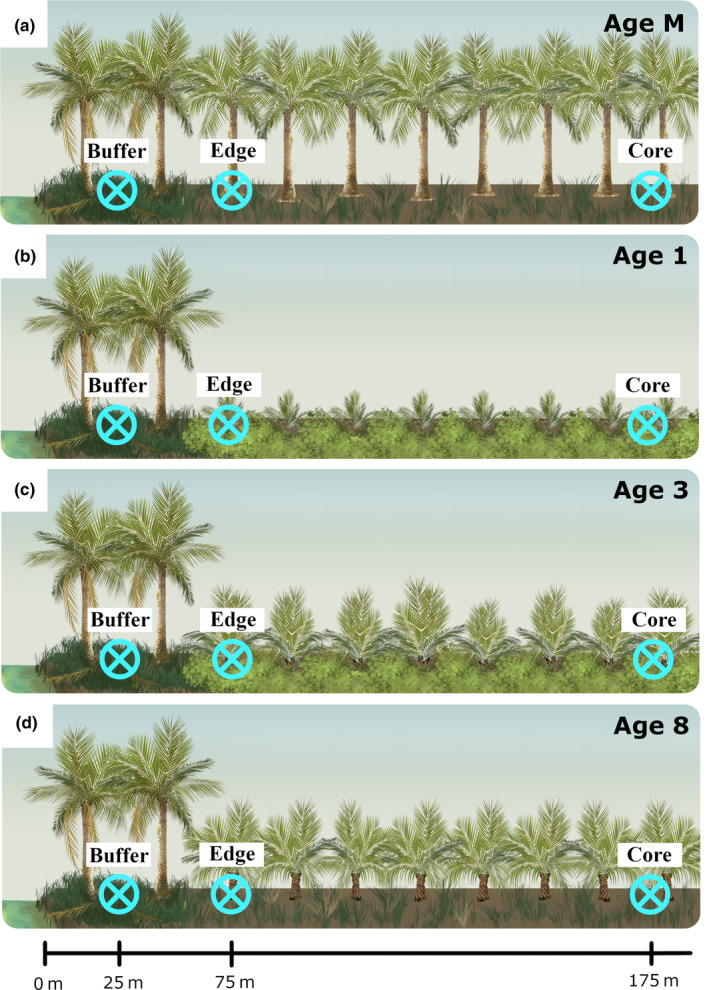
Schematic of the experimental design (Riau, Sumatra, Indonesia). We established study sites across four oil palm age cohorts: (a) Age M, (b) Age 1, (c) Age 3, (d) Age 8. All study sites were located near rivers and neighboring mature oil palm riparian buffers. We established a triplet of 100‐m long transects within each site that followed the course of the adjacent river and along which data collection occurred. Transects were located at three distances from rivers: within riparian buffers (Buffer), just outside buffers within adjacent oil palm crop (Edge), and far from riparian buffers within oil palm crop (Core). All Buffer areas were composed of mature first‐generation oil palms and were managed less intensely than the surrounding non‐buffer area

To understand the impacts of mature palm buffers on oil palm systems across the crop commercial life cycle, we assessed differences in environmental conditions and biodiversity between buffers and surrounding cultivated areas across four cohorts in a space‐for‐time study design. Cohorts were first‐generation mature oil palms that were nearing the end of their commercial life cycle (aged 31–33; Age M), and second‐generation replanted oil palms aged 1, 3, and 8 years (Age 1, Age 3, Age 8; Figure 1). We established four study sites (hereafter, Sites) in each cohort. Every site was located near a river with a neighboring mature palm buffer (i.e., no sites were located on rivers without buffers). We split sites within a cohort across two estates to account for differences in local management (see Pashkevich et al. [2021a] for details on differences in estate management across the sites). However, spatial constraints allowed only three Age 8 sites within one estate, resulting in 15 sites in total (Appendix S1: Figure S1). Also due to spatial constraints, two Age 1 sites were located within 100 m of each other, and two Age 8 sites were 135 m apart. All other sites were at least 300 m apart.

We established three 100‐m long transects within each site that followed the course of the adjacent river (Figure 1). Transects were located at three distances from the river edge (hereafter, Distance): within riparian buffers (Buffer; 25 m from riverbanks), just outside buffers within the surrounding cultivated area (Edge; 75 m from riverbanks and 25 m from buffers; and receiving typical levels of management), and far from buffers in the core of the plantation landscape (Core; 175 m from riverbanks and 125 m from buffers; and receiving typical levels of management). Hereafter, we collectively refer to Edge and Core as non‐buffer areas. We included Edge as a sampling area to assess whether buffers affected environmental conditions and arthropod biodiversity in non‐buffer areas immediately adjacent to mature oil palm buffers. We inferred that buffers affected Edge if, in comparison to Core, environmental conditions and arthropod biodiversity in Edge were more similar to those in Buffer. We assumed no influence of mature palm buffers on Core areas, as a previous study indicated that 125 m from buffers was an appropriate distance at which any impacts of buffers would no longer be detected (Gray et al., 2016).

### Data collection

#### Environmental conditions

All fieldwork occurred between February and May 2018. Mean weekly rainfall (± 1 SE) during the study period was 25.9 ± 2.3 mm (collected from nine rainfall gauges across SMARTRI).

Within each of the Buffer, Edge, and Core transects we measured canopy openness, vegetation height, and ground cover at 10‐m intervals. This corresponded to 11 data points per transect. We recorded canopy openness using a spherical densiometer (Lemmon, 1956). We alternated pointing the densiometer to the left and right of each transect as we measured. We recorded vegetation height using a drop disc (30 cm diameter and 231 g, dropped from 170 cm above the ground). Where the drop disc fell, we categorized the type(s) of ground cover touching the disc as: fallen palm frond, herbaceous plant, fern, bare ground, or water‐filled ditch. Openness, vegetation height, and ground cover data from two sites (one each in Age M and Age 1) could not be collected so, in order to retain statistical power, we included data from a comparable Age M site that were collected during the same study period (data from an additional Age 1 site were not available). Canopy openness, vegetation height, and ground cover data were therefore recorded at 14 sites in total. We used iButton dataloggers (HomeChip; DS1922L‐F5 thermochrons set at high capacity and programmed to record at three‐hourly intervals) to record soil temperatures at Buffer and Core transects. We did not sample in Edge owing to limited availability of dataloggers, and because canopy openness data suggested that soil temperatures between Edge and Core would not differ. We buried one datalogger at 5 cm depth at the start of each transect, retrieving both dataloggers at a site after 24 h. Each datalogger recorded eight temporal data points. Soil temperature data from two Age M sites could not be collected, and three dataloggers overheated in the field (two in Age 1 and one in Age 3). To retain statistical power, we included soil temperature data from a comparable Age M site that were collected during the same study period (data from additional Age 1 and Age 3 sites were not available), and therefore soil temperature was recorded at 11 sites in total.

#### Order‐level arthropod sampling

We collected arthropods along Buffer, Edge, and Core transects at all sites in canopy, understory, and ground microhabitats. We collected canopy arthropods by fogging one palm at the start of each transect (Pulsfog K‐10‐SP Portable Thermal Fogger filled with 4.950 L diesel and 50 mL lambda‐cyhalothrin insecticide). We fogged for 60 s after the canopy was completely covered with insecticide, and then waited 2 h before collecting arthropods from six trays that were systematically suspended ~1 m above the ground under each palm (the total tray area under each sampled palm was 4.74 m^2^). We kept fogging methods consistent across cohorts so that samples were comparable, and to ensure that a consistent area of vertical space was sampled for each palm (for more details of the fogging protocol see Pashkevich et al. [2021a]). To collect understory arthropods, we suspended sticky traps (each brown in color, sticky on both sides, and having a total sticky area of 513 cm^2^) ~ 1.5 m above the ground at the start, middle, and end of each transect. To sample ground arthropods, we placed a single pitfall trap (19.5 cm diameter at mouth, filled with 70% ethanol, and covered with a plastic sheet to shield from rainfall) adjacent to each sticky trap. We set all sticky traps and pitfall traps at a Site on the same day, and collected them after 72 h.

In the lab, we used dissecting microscopes to separate and identify arthropods to order level. However, to be consistent with comparable studies (Ashton‐Butt et al., 2019; Pashkevich et al., 2021a) and due to their distinctive ecology, we identified the following to groups of their own: Chilopoda (class), Diplopoda (class), Formicidae (family), and Isoptera (infraorder). Endopterygote larvae were also placed in their own group. We hereafter refer to all groups as orders, for brevity.

#### Species‐level spider sampling

We also conducted focused surveys of spiders along Buffer, Edge, and Core transects at all sites in canopy, understory, and ground microhabitats. We sampled canopy and ground spiders by separating them from other arthropods in fogging and pitfall samples. As sticky traps often damaged understory spiders and did not collect a high number of individuals, we collected understory spiders by walking the length of each transect and hand‐collecting all spiders within 1 m of the observer. We walked all transects at a Site on the same day and sampled between 07:00 and 14:00 in dry conditions.

In the lab, we separated juvenile spiders from adults (dissecting when necessary to differentiate haplogyne adult females and juveniles), and used morphological traits and the relevant keys (Deeleman‐Reinhold, 2001; Jocqué & Dippenaar‐Schoeman, 2006; http://ecotaxonomy.org/ecokeys) to identify adults to family and morphospecies (hereafter, species). Since it was not possible to match males and females for all species, we counted each unique male and female as its own morphospecies. All arthropods are preserved in 75% ethanol at SMARTRI research station (Siak Regency, Riau, Indonesia).

### Statistical approach

We conducted all analyses in R version 3.5.1 (R Core Team, 2018) using R Studio version 1.1.456 (R Studio Team, 2018). We used *readxl* (Wickham et al., 2019), *tidyverse* (Wickham, 2017), *zoo* (Zeileis et al., 2019), *data.table* (Dowle et al., 2019), *lattice* (Sarkar et al., 2020), and *plyr* (Wickham, 2016) for data wrangling and exploration, following the data exploration procedure outlined by Zuur et al. (2010). Visualizing our results required packages *gridExtra* (Auguie & Antonov, 2017), *cowplot* (Wilke, 2019), and *lemon* (Edwards et al., 2019). We analyzed our data using the following techniques (additional details on individual models are described below):

#### Bayesian regression models (hereafter, GLMMs)

We fitted GLMMs using *brms* (Bürkner & Gabry, 2020) and the No‐U‐Turn sampler (NUTS) algorithm in Stan (Carpenter et al., 2017). We fitted five candidate models for each response: a parent model (Cohort × Distance), and four derivative models (Cohort + Distance, a Cohort‐only model, a Distance‐only model, and a null model). Unless otherwise stated, we included Site as a random intercept effect in all models, to account for potential spatial autocorrelation, site‐specific differences in environmental conditions and timing of sampling in our modeling. After generating and validating each set of models (see Appendix S1: Section S1 for full details of model fit and validation), we calculated their exact leave‐one‐out cross‐validation information criterion (LOOIC) and selected the model with the lowest LOOIC as the optimal model, unless the standard errors of the difference in expected log pointwise predictive density (ELPD) of other models exceeded the difference in ELPD between these models and the model with the lowest LOOIC (Gabry et al., 2019). In this case, we chose the simplest model. We calculated a Bayesian version of *R* ^2^ for each optimal model in order to approximate the percent variance in the response that each model explained. If the null model was not the optimal model, we used *emmeans* (Lenth et al., 2020) to conduct post‐hoc analyses by computing estimated marginal means for each factor level and comparing these in a pairwise fashion. We concluded that factor levels were meaningfully different if the 95% highest posterior density (HPD) interval of the median point estimate calculated from our comparisons did not overlap with zero.

#### Bayesian generalized linear latent variable model (hereafter, GLLVM)

We fitted a pure (i.e., no covariates included) GLLVM using *boral* (Hui & Blanchard, 2020; see Appendix S1: Section S1). We included Site as a random row effect, in order to account for potential spatial autocorrelation and site‐specific differences in environmental conditions in our modeling, and two latent variables (LVs). We plotted a two‐dimensional ordination from the posterior medians of the LVs in order to visualize the results of our analysis. To aid visualization, we drew polygons around each Cohort × Distance combination of points (e.g., Age 1‐Buffer). To determine factor levels that were meaningfully different, we compared the spatial positions of polygons in a pairwise fashion. We concluded that meaningful differences existed when polygons did not overlap.

#### Multivariate generalized linear models (hereafter, mGLMs)

We fitted mGLMs using *mvabund* (Wang et al., 2019), with the interaction of Cohort × Distance. After validating models (see Appendix S1: Section S1), we used likelihood ratio tests (LRTs) and bootstrapped probability integral transform (PIT) residuals (using 10,000 resampling iterations; Warton et al., 2017) to determine any significant effects of covariates. We included Site as a blocking variable when calculating *p* values, in order to account for potential spatial autocorrelation, site‐specific differences in environmental conditions, and timing of sampling in our modeling. We followed a backward‐stepwise model selection procedure to determine whether the interaction of Cohort × Distance, or either covariate independently, was significant (*p* < 0.05). When covariates were significant, we conducted post‐hoc analyses to determine factor levels that were significantly different.

#### Impacts of mature palm buffers across cohorts on the environment

We used GLMMs to analyze changes in canopy openness, variation in openness, and vegetation height. We did not include Site as a random effect in our canopy openness and variation in openness analyses, as there was no reason to expect that these data would be non‐independent within sites, and sensitivity analyses showed that models fitted without Site performed equally well (Appendix S1: Table S1). The vast majority (82.5%) of canopy openness values were from mostly open (i.e., >80% openness) or mostly closed (i.e., <20% openness) areas, resulting in a bimodal distribution. We therefore transformed these data into a binary variable (Open canopy: “1”, or >50% openness; Closed canopy: “0”, or <50% openness), summed values along each transect, and modeled canopy openness using a binomial distribution. Our models were overdispersed, and so we re‐fitted models using beta‐binomial distributions (logit links; Parent model: Canopy openness ~ Cohort × Distance). We analyzed variation in openness by calculating the standard deviation in raw openness data for each transect and modeling these data using a normal distribution (identity links), after applying a logit transformation in order to meet model assumptions (Parent model: Variation in openness ~ Cohort × Distance). We analyzed mean vegetation height per transect using a Gaussian distribution (identity links), after applying a logit transformation in order to meet model assumptions (Parent model: Vegetation height ~ Cohort × Distance + [1 | Site]). We used a GLLVM (Poisson distribution, log link) to analyze changes in ground cover (Parent model: Ground cover ~ LV1 + LV2 + [1 | Site]). We analyzed ground cover using a GLLVM so that we could visualize differences in this multivariate data set as ordinations. We analyzed changes in soil temperature using a GLMM, which included smoothing functions (using cyclic penalized cubic regression splines) fitted to the time of day at which recording occurred (Parent model: Temperature ~ Cohort + Distance + s(Time, by = interaction(Cohort, Distance) + (1 | Site)).

#### Impacts of mature palm buffers across cohorts on all arthropods

While conducting fieldwork, three sticky traps (Age 8‐Edge, Age 8‐Buffer, Age M‐Buffer) and one pitfall trap (Age 8‐Edge) were damaged in the field and removed from analyses. We also eliminated Formicidae from an additional sticky trap sample because a high number (*n* = 278) of winged ants had emerged from their nest and flown into the trap. When fogging, we lost data from seven sample trays across three palms because they were overturned before collection. We therefore standardized total canopy arthropod abundance data prior to analysis by calculating mean abundance per tray and multiplying by 6 (the number of trays originally set under each palm). To meet model assumptions, we then rounded the standardized data to the nearest integer.

We used GLMMs (negative binomial distributions, log links) to separately analyze changes in total arthropod abundance in the canopy, understory, and ground microhabitats (Parent models: Abundance ~ Cohort × Distance + (1 | Site)). We included all collected arthropods in our total abundance analyses. We used mGLMs (negative binomial distributions, log links) to separately analyze changes in arthropod order‐level community composition in the canopy, understory, and ground microhabitats (Parent models: Composition ~ Cohort × Distance + (1 | Site)). In these analyses, we excluded endopterygote larvae and individuals that could not be identified to order (together representing about 3% of all collected arthropods). We separately aggregated understory and ground data at the transect level prior to fitting mGLMs. We aggregated these data because otherwise *mvabund* would not allow us to fit Site as a blocking variable in our analyses, and we wanted to account for site‐specific differences in environmental conditions and timing of sampling that could have impacted arthropod composition. We aggregated these data in a standardized way by calculating mean abundance per trap, multiplying by 3 (the number of pitfall traps and sticky traps originally set along each transect), and rounding to the nearest integer, in order to meet model assumptions and account for the sticky traps and pitfall trap that were damaged during fieldwork. If the interaction term (i.e., Cohort × Distance) or either covariate was significant in our community composition analyses, we ran univariate analyses to determine how the abundance of individual taxa changed across study areas. Univariate *p* values were adjusted to correct for multiple testing using a step‐down resampling algorithm (Wang et al., 2012). We visualized the results of our community composition analyses using stacked bar charts.

#### Impacts of mature palm buffers across cohorts on spiders

To better understand the spider assemblage within the plantation, and to assess our sampling completeness, we used *iNEXT* (Hsieh et al., 2016) to calculate interpolated and extrapolated species richness within each microhabitat (using the richness estimators derived by Chao et al., 2014) and plotted these as species accumulation curves. We extrapolated to double the number of observed individuals (Chao et al., 2014; Gotelli & Colwell, 2001). We also assessed species evenness within each microhabitat by plotting rank abundance curves. We included only adult spiders in both analyses.

We used GLMMs to separately analyze changes in spider abundance (negative binomial distributions, log links) and species richness (Poisson distributions, log links) in the canopy, understory, and ground microhabitats (Parent models: Abundance ~ Cohort × Distance + (1 | Site); Richness ~ Cohort × Distance + (1 | Site)). We included juveniles and adults in abundance analyses but only adults in species richness analyses. We adjusted canopy abundance and species richness data to account for overturned trays, as previously described. We eliminated juvenile wolf spiders (Lycosidae) from one pitfall sample (Age M‐Core) prior to analyzing ground abundance data, due to an unusually high abundance of juveniles (*n* = 61) that were likely to have been on the abdomen of their mother when she fell into the trap. We assessed changes in spider species‐level composition within each microhabitat in two ways, including only adult spiders in these analyses. First, using *betapart* (Baselga et al., 2020), we calculated overall incidence‐based beta diversity (Sørensen index) across Cohort × Distance groups within each microhabitat, partitioning this value into nestedness (i.e., species loss or gain) and turnover (i.e., species replacement) components (Baselga & Orme, 2012). Data from different sites within a Cohort × Distance group (e.g., all sites within Age 1‐Buffer) were pooled for this analysis. We then used mGLMs (negative binomial distributions, log links) to separately analyze changes in species‐level community composition in the canopy, understory, and ground microhabitats (Parent models: Composition ~ Cohort × Distance + (1 | Site)). Prior to analysis, and as previously described, we aggregated ground spider data at the transect level so that Site could be fitted as a blocking variable. We did not aggregate canopy or understory spider data, since these were collected at the transect level. Our spider community composition analyses otherwise were unchanged from our arthropod community composition analyses.

## RESULTS

### Impacts of mature palm buffers across cohorts on the environment

**FIGURE 2:**
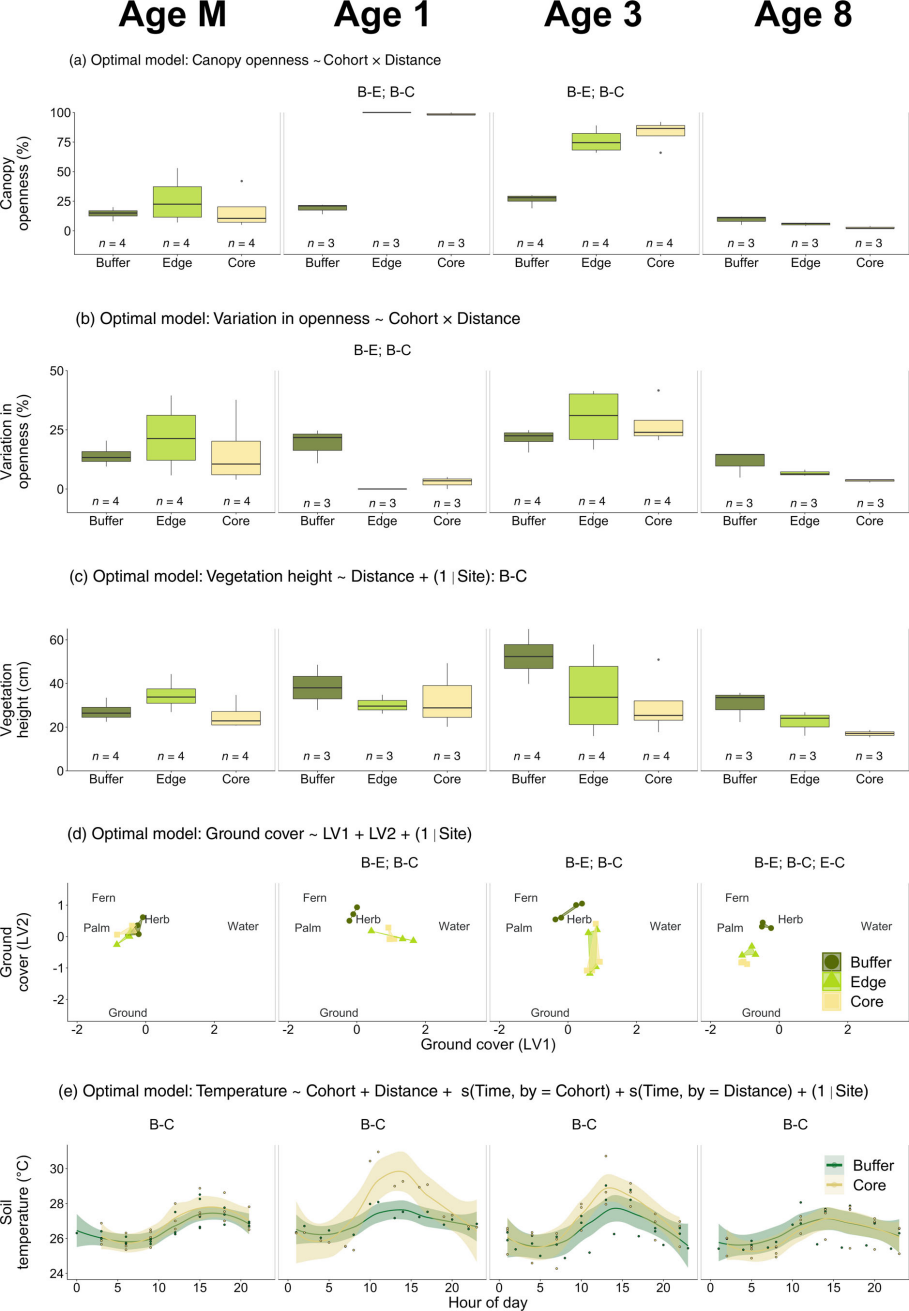
Differences in (a) canopy openness, (b) variation in openness, (c) vegetation height, (d) ground cover, and (e) soil temperature across cohorts (Age M, Age 1, Age 3, Age 8) and distances to riparian buffers (Buffer, Edge, Core). Posterior distributions from all GLMMs tracked to their underlying data sets. We indicate the optimal model (determined using LOOIC) in text above each subplot. The optimal model for panel d was not determined using LOOIC, as we did not follow a model selection procedure for our ground cover analyses. In panels a, b, d, and e, differences between Buffer (B), Edge (E), and Core (C) within each cohort (as determined by our post‐hoc analyses) are indicated in text above each facet. In panel c, differences between Buffer, Edge, and Core across cohorts (as determined by our post‐hoc analyses) are provided after the optimal model, since the optimal model included only Distance. See Supplementary Materials (Appendix S1: Tables S3, S5, S7, S9) for a full list of post‐hoc comparisons between Buffer, Edge, and Core areas across cohorts. In panels a, b, and c, boxplots display the median and interquartile ranges of the data, and lettering below boxplots indicates the number of independent replicates per Cohort × Distance (e.g., Age 1‐Buffer). In panel d, points indicate the posterior medians of the latent variables from the pure GLLVM that we fitted to analyze ground cover data (Fern, ferns; Herb, herbaceous plants; Palm, dead and fallen palm fronds; Ground, bare ground; Water, water‐filled ditch). Polygons are drawn around outlying points from the same Cohort × Distance combination, in order to aid visualization. In panel e, lines are visualizations of differences in soil temperature (generated using loess smoothers in *ggplot*; Wickham et al., 2019), shaded regions around lines indicate 95% credible intervals, and black circles indicate raw data points. LV, latent variable; s, smoothing function. Soil temperatures were only recorded in Buffer and Core

All environmental conditions differed between buffer and non‐buffer areas, but the magnitude of these differences changed across cohorts. The model that included an interaction between Cohort and Distance best explained differences in canopy openness (*R* ^2^ = 90.9% ± 2.1%; Table 1; Appendix S1: Tables S1, S2) and variation in openness (*R* ^2^ = 67.1% ± 5.8%; Table 1; Appendix S1: Tables S1, S4). Post‐hoc analyses from the canopy openness model showed that openness differed between buffer and non‐buffer areas in Ages 1 and 3, with openness per transect in Age 1‐Edge and Age 1‐Core being more than 400% higher than in Age 1‐Buffer, and openness per transect in Age 3‐Edge and Age 3‐Core being more than 180% higher than in Age 3‐Buffer (Figure 2a; Appendix S1: Table S3). Post‐hoc analyses from the variation in openness model showed that variation differed between buffer and non‐buffer areas in *Age 1*, with variation per transect in Age 1‐Buffer being more than 570% higher than in Age 1‐Edge and Age 1‐Core (Figure 2b; Appendix S1: Table S5).

**TABLE 1:** Effects of Cohort and Distance on environmental conditions (canopy openness, variation in openness, vegetation height, ground cover, and soil temperature) and arthropod biodiversity (total arthropod abundance, arthropod order‐level community composition, and spider abundance, species richness, and species‐level community composition) in the canopy, understory, and ground microhabitats

Response	Optimal model	Bayesian *R* ^2^ ± Estimate Error
Environmental conditions		
Canopy openness	~ Cohort × Distance	90.9% ± 2.1%
Variation in openness	~ Cohort × Distance	67.1% ± 5.8%
Vegetation height	~ Distance + (1 | Site)	39.8% ± 12.8%
Ground cover	~ 1 + LV1 + LV2 + (1 | Site)	‐
Soil temperature	~ Cohort × Distance + s(Time, by = Cohort) + s(Time, by = Distance) + (1 | Site)	74.1% ± 2.2%
All arthropods abundance		
Canopy	~ 1 + (1 | Site)	40.8% ± 10.1%
Understory	~ Cohort × Distance + (1 | Site)	51.0% ± 4.6%
Ground	~ 1 + (1 | Site)	34.9% ± 10.9%
All arthropods composition		
Canopy	~ 1 + (1 | Site)	‐
Understory	~ Cohort + (1 | Site)	‐
Ground	~ Cohort + (1 | Site)	‐
Spider abundance		
Canopy	~ 1 + (1 | Site)	31.4% ± 14.8%
Understory	~ Cohort × Distance + (1 | Site)	7.14% ± 4.9%
Ground	~ 1 + (1 | Site)	23.6% ± 7.7%
Spider species richness		
Canopy	~ 1 + (1 | Site)	51.3% ± 7.8%
Understory	~ Cohort + (1 | Site)	48.1% ± 9.7%
Ground	~ 1 + (1 | Site)	26.2% ± 6.0%
Spider composition		
Canopy	~ Cohort × Distance + (1 | Site)	…
Understory	~ Cohort × Distance + (1 | Site)	…
Ground	~ Cohort × Distance + (1 | Site)	…

*Note*: For each model, we present its Bayesian *R* ^2^ value and associated standard error. No *R* ^2^ values are given for our ground cover and community composition analyses, as this is not a feature supported by the packages that facilitated these analyses. LV, latent variable; s, smoothing function.

The Distance‐only model best explained differences in vegetation height, indicating that vegetation height differed between buffer and non‐buffer areas independently of cohort (*R* ^2^ = 39.8% ± 12.8%; Table 1; Appendix S1: Table S6). Post‐hoc analyses showed that vegetation height differed between Buffer and Core, with vegetation in Buffer being 11% higher than in Core (Figure 2c; Appendix S1: Table S7). Ground cover was different between buffer and non‐buffer areas in Ages 1, 3, and 8. This was indicated by the spatial separation of polygons in these cohorts in the ordination generated from our GLLVM. In Ages 1 and 3, Buffer was consistently dominated by ferns (mostly *Nephrolepis biserrata*, *Asplenium longissimum*, and *Dicranopteris linearis*), which represented ~50% of all vegetation type occurrences. However, in Edge and Core, herbaceous plants (mostly the leguminous cover crop) represented ~70%–95% of all vegetation type occurrences. Age 8‐Edge and Age 8‐Core differed from Age 8‐Buffer, and from each other, owing to higher occurrences of bare ground (Figure 2d).

The additive model best explained changes in soil temperature (*R* ^2^ = 74.1% ± 2.2%; Table 1; Appendix S1: Table S8). This indicated that soil temperature exhibited consistent trends between Buffer and Core in all cohorts. Post‐hoc analyses showed that soil temperatures in Core were consistently hotter than in Buffer. This was most pronounced in Age 1, where soil temperatures in Age 1‐Core were ~2°C hotter at midday than in Age 1‐Buffer (Figure 2e; Appendix S1: Table S9).

### Impacts of mature palm buffers across cohorts on all arthropods

**FIGURE 3:**
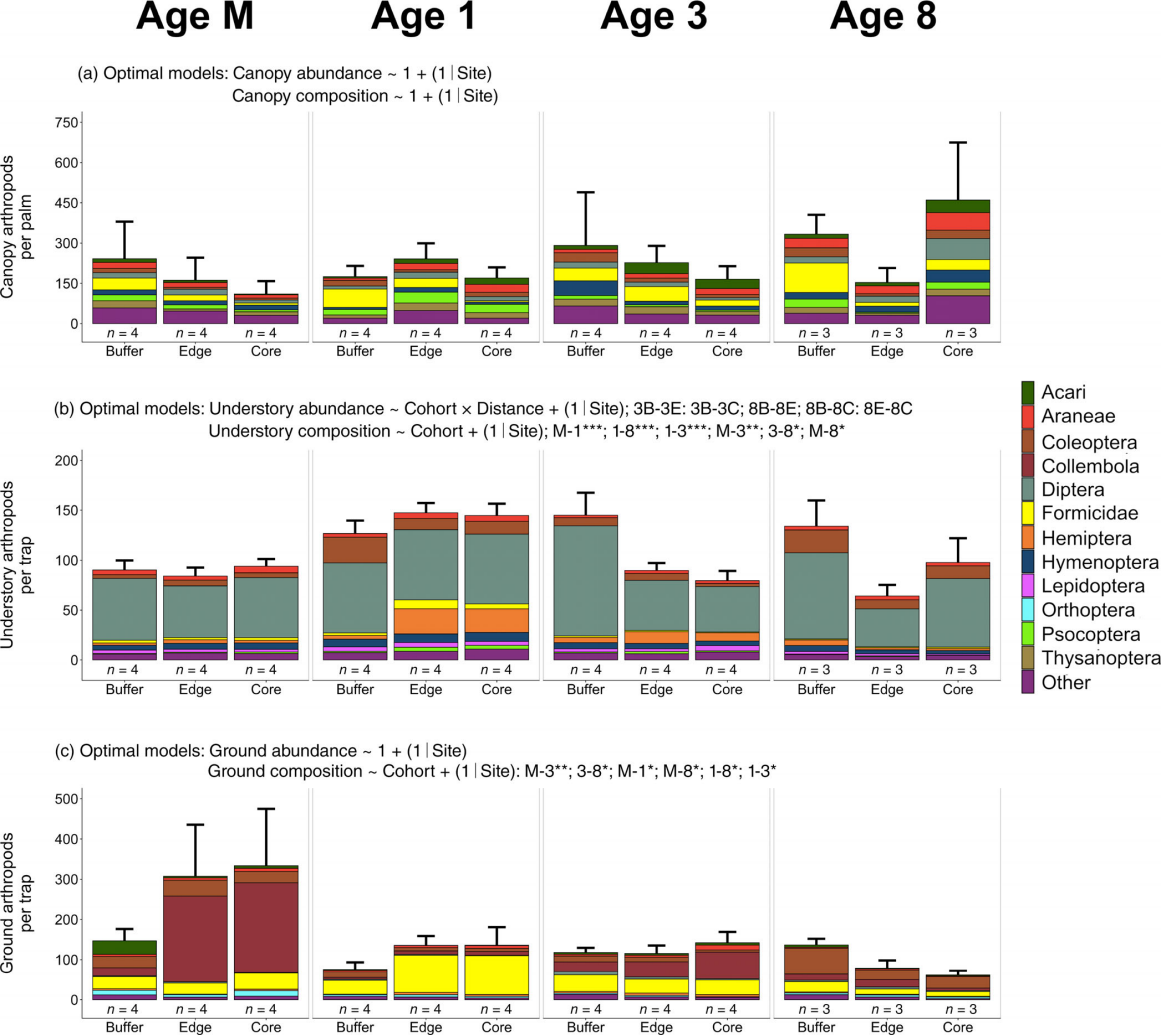
Differences in total arthropod abundance and arthropod order‐level community composition in the (a) canopy, (b) understory, and (c) ground microhabitats across cohorts (Age M, Age 1, Age 3, Age 8) and distances to riparian buffers (Buffer, Edge, Core). Posterior distributions from all GLMMs tracked to their underlying data sets. We indicate the optimal model for total abundance (determined using LOOIC) and order‐level composition (determined using backward stepwise selection) in text above each subplot. If the null model was not the optimal model, we list factor levels within each cohort between which significant differences occurred (as determined by our post‐hoc analyses): Age M (M), Age 1 (1), Age 3 (3), Age 8 (8), Buffer (B), Edge (E), and Core (C). For order‐level community composition analyses, we also list the magnitude of significance for post‐hoc comparisons: *** *p* < 0.001, **p < 0.01, *p < 0.05. See Supplementary Materials (Appendix S1: Tables S17, S18, S19) for a full list of post‐hoc comparisons between Buffer, Edge, and Core areas across cohorts. Arthropods are plotted as they are sequenced in the legend. Error bars indicate one standard error from the mean. Lettering below stacked bars indicates the number of independent replicates per Cohort × Distance (e.g., Age 1‐Buffer). Data for panels (b) and (c) are plotted at the trap level, but note that data were aggregated at the transect level for order‐level community composition analyses

In our order‐level sampling (canopy fogging, sticky traps, and pitfall traps), we collected 44,984 arthropods that were identified to 26 orders or other taxonomic groups. These included 9970 arthropods from the canopy (after correcting for overturned fogging trays; Appendix S1: Table S10), 14,473 arthropods from the understory (Appendix S1: Table S11), and 20,541 arthropods from the ground (Appendix S1: Table S12). Total arthropod abundance in the canopy and on the ground differed little between buffer and non‐buffer areas across cohorts, with the null model being the optimal model for both (*R* ^2^ = 40.8% ± 10.1% in the canopy; *R* ^2^ = 34.9% ± 10.9% on the ground; Figure 3a,c; Table 1; Appendix S1: Tables S13, S14, S16). In the understory, the interaction model best explained differences in total arthropod abundance (*R* ^2^ = 51.0% ± 4.6%; Table 1; Appendix S1: Tables S13, S15). Post‐hoc analyses showed that total understory arthropod abundance differed between buffer and non‐buffer areas in Ages 3 and 8, with arthropods per trap in Age 3‐Buffer being 61% and 81% more abundant than in Age 3‐Edge and Age 3‐Core, respectively, and arthropods per trap in Age 8‐Buffer being 108% and 36% more abundant than in Age 8‐Edge and Age 8‐Core, respectively (Figure 3b; Appendix S1: Table S17).

Different arthropod orders were dominant within each microhabitat. Formicidae (*n* = 1717), Araneae (*n* = 1056), and Diptera (*n* = 976) were numerically dominant in the canopy; Diptera (*n* = 8865), Coleoptera (*n* = 1384), and Hemiptera (*n* = 1132) were dominant in the understory (after aggregating data at the per‐transect level); and Collembola (*n* = 7677), Formicidae (*n* = 5751), and Coleoptera (*n* = 2932) were dominant on the ground (after aggregating data at the per‐transect level). Arthropod order‐level composition in the canopy did not differ across buffer and non‐buffer areas or across cohorts, as no model terms were significant (*p* > 0.05; Figure 3a; Table 1). The optimal model for order‐level composition of understory arthropods included only cohort (Cohort: LRT = 224.0, *p* < 0.001; Figure 3b; Table 1), indicating that order‐level composition differed significantly across cohorts in the chronosequence but did not differ significantly between buffer and non‐buffer areas. Post‐hoc analyses indicated that all cohorts differed significantly in order‐level composition from each other (*p* < 0.05; Appendix S1: Table S18), and univariate analyses showed that these trends were driven by changed abundances of Blattodea (LRT = 24.185, *p* = 0.016), Coleoptera (LRT = 22.685, *p* = 0.022), Formicidae (LRT = 22.852, *p* = 0.022), Hemiptera (LRT = 31.013, *p* = 0.004), Lepidoptera (LRT = 15.632, *p* = 0.039), and Psocoptera (LRT = 30.112, *p* = 0.005). A similar trend was found on the ground, as the optimal model for order‐level composition of ground arthropods included only cohort (Cohort: LRT = 228.1, *p* = 0.008; Figure 3c; Table 1). Post‐hoc analyses indicated that all cohorts differed significantly in order‐level composition from each other (*p* < 0.05; Appendix S1: Table S19), and univariate analyses indicated that these trends were driven by different abundances of Acari (LRT = 27.704, *p* = 0.027), Coleoptera (LRT = 33.463, *p* = 0.010), Collembola (LRT = 25.463, *p* = 0.042), Formicidae (LRT = 22.686, *p* = 0.042), and Orthoptera (LRT = 24.278, *p* = 0.042).

### Impacts of mature palm buffers across cohorts on spiders

We collected 4112 spiders that were identified to 22 families and 219 species. These included 1040 spiders from the canopy (*n*_adults_ = 245; *n*_species_ = 98), 2346 spiders within the understory (*n*_adults_ = 713; *n*_species_ = 80), and 726 spiders on the ground (*n*_adults_ = 374; *n*_species_ = 73). Species accumulation curves in all microhabitats were starting to asymptote, indicating that we had sampled a high proportion of all species within each microhabitat (an estimated 56.2% of species in the canopy, 67.1% of species in the understory, and 70.9% of species on the ground; Appendix S1: Figure S3). Rank abundance curves indicated that species evenness in all microhabitats was low, with a few numerically dominant species representing the majority of individuals within each microhabitat. Species evenness was lowest on the ground (one species of Oonopidae represented 35.3% of all adult individuals) and highest in the canopy (no single species represented more than 7% of all adult individuals) (Appendix S1: Figure S3).

**FIGURE 4:**
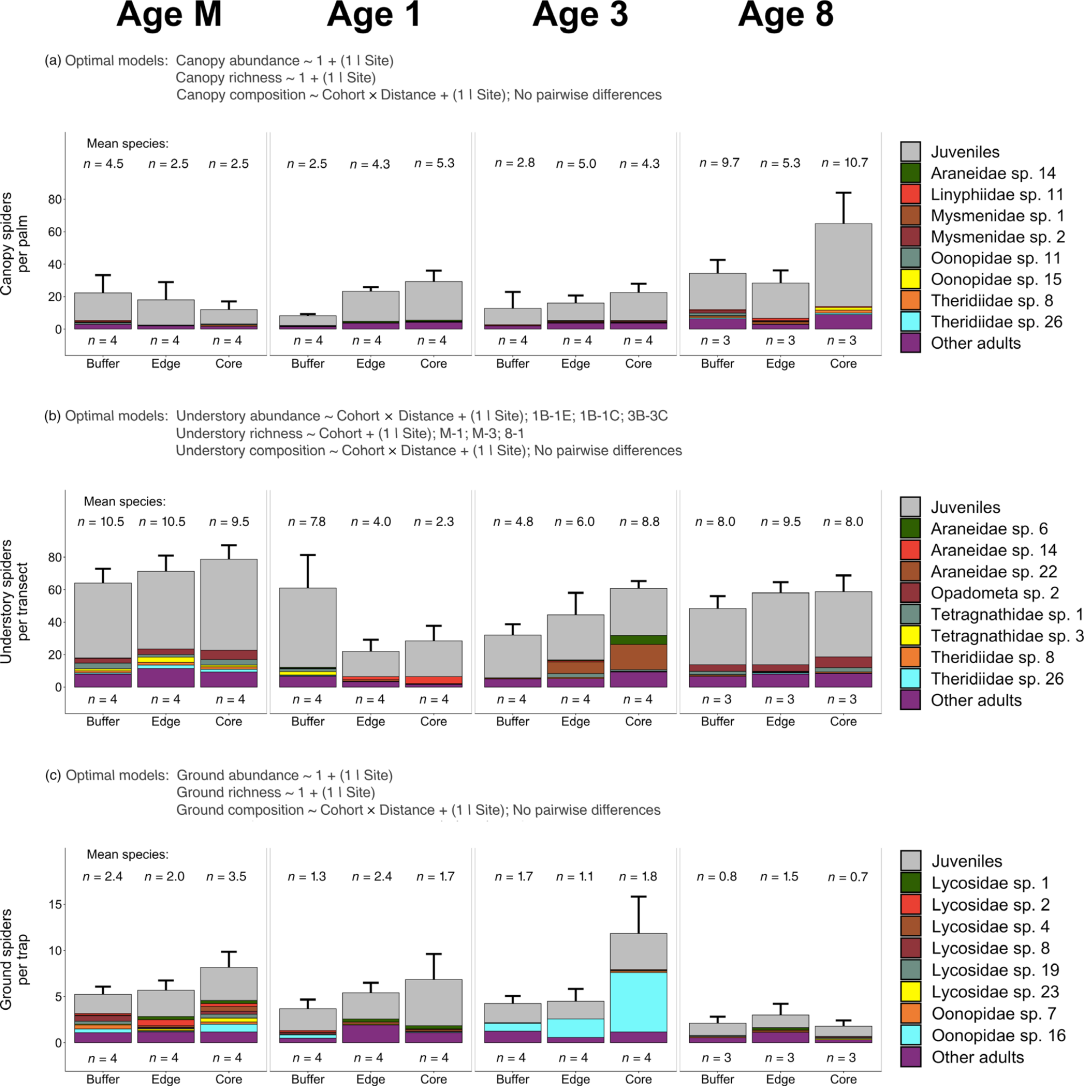
Differences in spider abundance, species richness, and species‐level community composition in the (a) canopy, (b) understory, and (c) ground microhabitats across cohorts (Age M, Age 1, Age 3, Age 8) and distances to riparian buffers (Buffer, Edge, Core). Posterior distributions from all GLMMs tracked to their underlying data sets. We indicate the optimal model for spider abundance and species richness (determined using LOOIC) and species‐level composition (determined using backward stepwise selection) in text above each subplot. If the null model was not the optimal model, we list factor levels within each cohort between which differences occurred (as determined by our post‐hoc analyses): Age M (M), Age 1 (1), Age 3 (3), Age 8 (8), Buffer (B), Edge (E), and Core (C). See Supplementary Materials (Appendix S1: Tables S24, S28, S29, S30, S31) for a full list of post‐hoc comparisons between Buffer, Edge, and Core areas across cohorts. Spiders are plotted as they are sequenced in the legend. Error bars indicate one standard error from the mean. Lettering below stacked bars indicates the number of independent replicates per Cohort × Distance (e.g., Age 1‐Buffer). Data for panel (c) are plotted at the trap level, but note that data were aggregated at the transect level for species‐level community composition analyses

Spider abundance in the canopy and on the ground did not differ between buffer and non‐buffer areas, with the null model being the optimal model for both (*R* ^2^ = 31.4% ± 14.8% in the canopy; *R* ^2^ = 23.6% ± 7.7% on the ground; Figure 4a,c; Table 1; Appendix S1: Tables S20, S21, S23). In the understory, the interaction model best explained differences in spider abundance (*R* ^2^ = 71.4% ± 4.9%; Table 1; Appendix S1: Tables S20, S22). Post‐hoc analyses showed that understory spider abundance differed between buffer and non‐buffer areas in Ages 1 and 3. Spider abundance per transect in Age 1‐Buffer was 177% higher than in Age 1‐Edge and 114% higher than in Age 1‐Core, and spider abundance per transect in Age 3‐Core was 90% higher than in Age 3‐Buffer (Figure 4b; Appendix S1: Table S24).

Spider species richness in the canopy and on the ground did not differ between buffer and non‐buffer areas, with the null model being the optimal model for both (*R* ^2^ = 51.3% ± 7.8% in the canopy; *R* ^2^ = 26.2% ± 6.0% on the ground; Figure 4a,c; Table 1; Appendix S1: Tables S20, S25, S27). In the understory, the Cohort‐only model best explained differences in spider species richness (*R* ^2^ = 48.1% ± 9.7%; Table 1; Appendix S1: Tables S20, S26). Post‐hoc analyses showed that understory spider species richness differed between Age M and Ages 1 and 3, and Age 1 and Age 8. Species richness per transect in Age M was 120% higher than in Age 1 and 58% higher than in Age 3, and species richness per transect in Age 8 was 78% higher than in Age 1 (Figure 4b; Appendix S1: Table S28).

Different spider species were dominant within each microhabitat. A theridiid (*Theridiidae* sp. 8, *n* = 16), mysmenid (*Mysmenidae* sp. 2, *n* = 15), and linyphiid species (*Linyphiidae* sp. 11, *n* = 14) were numerically dominant in the canopy; an araneid (*Araneidae* sp. 22, *n* = 96), and two species of tetragnathid (*Opadometa* sp. 2, *n* = 95; *Tetragnathidae* sp. 1, *n* = 70) were numerically dominant in the understory, and an oonopid (*Oonopidae* sp. 16, *n* = 132) and three lycosid species (*Lycosidae* sp. 1, *n* = 18; *Lycosidae* sp. 2 and *Lycosidae* sp. 6, each *n* = 17) were numerically dominant on the ground (after aggregating data at the per‐transect level). Total dissimilarity across Cohort × Distance groups (e.g., Age 1‐Buffer) in all microhabitats was relatively high (Sørensen index = 91.2% in the canopy, 85.5% in the understory, and 88.6% on the ground). In all microhabitats, the turnover (i.e., species replacement) component was higher than the nestedness (i.e., species loss or gain) component. Turnover was 88.7% in the canopy, 80.0% in the understory, and 84.6% on the ground, while nestedness was <6% in all microhabitats. This indicates that the relatively high dissimilarity within each microhabitat was due to substitution of species rather than species loss or gain across Cohort × Distance groups. Spider species‐level composition differed between buffer and non‐buffer areas in multiple cohorts, as the optimal model for canopy (Interaction term: LRT = 70.3, *p* = 0.002; Appendix S1: Table S29), understory (Interaction term: LRT = 166.4, *p* < 0.001; Appendix S1: Table S30), and ground (Interaction term: LRT = 79.5, *p* = 0.002; Appendix S1: Table S31) spider species‐level composition included the interaction of Cohort × Distance (Table 1). Post‐hoc analyses indicated that no Cohort × Distance groups differed significantly in morphospecies‐level composition from each other (*p* > 0.05). Univariate analyses indicated that overall trends were driven by changed abundances of 6 species (1 araneid, 1 linyphiid, 2 oonopids, and 2 theridiids) in the canopy, 15 species (9 araneids, 5 tetragnathids, and 1 theridiid) in the understory, and 5 species (4 lycosids and 1 oonopid) on the ground (*p* < 0.05 for all species; Figure 4a–c; Appendix S1: Table S32).

### Impacts of mature palm buffers on adjacent edge areas

We found some evidence that buffers affected environmental conditions in adjacent non‐buffer areas (i.e., Edge). The optimal model for vegetation height (i.e., the Distance‐only model) indicated that vegetation in Buffer was taller than that in Core, and that vegetation height in Buffer and Edge was similar (Figure 2c). Ground cover in Age 8‐Buffer and Age 8‐Edge was more similar than ground cover in Age 8‐Buffer and Age 8‐Core. This was indicated by the Age 8‐Buffer and Age 8‐Edge polygons generated from our GLLVM being spatially closer than the Age 8‐Buffer and Age 8‐Core polygons; however, we note that the difference in spatial proximities was small (Figure 2d). We found no evidence that buffers affected levels of arthropod biodiversity in adjacent Edge areas, as arthropod abundance and composition in all microhabitats in Edge was no more similar to Buffer than Core arthropod abundance and composition.

### Changes within mature palm buffers over time

We found changes in canopy openness, variation in openness, and ground cover within mature palm buffers over time, but we did not find changes in other environmental conditions or any aspects of arthropod biodiversity. Post‐hoc analyses from the canopy openness optimal model showed that openness in buffers differed between Age 8 and Ages 1 and 3, with openness per transect in Age 1 and Age 3 buffers being 96% and 173% higher, respectively, than Age 8 buffers (Appendix S1: Figure S4A). Post‐hoc analyses from the variation in openness optimal model showed that variation in buffers differed between Ages 3 and 8, with variation per transect in Age 3 buffers being 88% higher than Age 8 buffers (Appendix S1: Figure S4B). Our ground cover analysis indicated that there were changes in ground cover within buffers over time. This was visualized by no spatial overlap of Buffer polygons between these respective ages (Appendix S1: Figure S4C). Buffers in Ages 1 and 3 were different from those in Ages M and 8 by having a higher occurrence of ferns. However, we note that all Buffer polygons that were generated from our GLLVM were close in proximity, indicating that between‐cohort differences in ground cover within buffers was limited.

## DISCUSSION

### Differences between mature palm buffers and surrounding habitats

This study is the first to investigate the ecology of riparian buffers made of mature oil palms that are being passively restored (“mature palm buffers”), a widespread, but little studied, management strategy used within plantations, across the oil palm commercial life cycle. We found that mature palm buffers can have greater environmental complexity and higher levels of arthropod biodiversity than non‐buffer areas (i.e., Edge and Core), particularly in recently replanted plantations, but these benefits are not consistent across the crop commercial life cycle. To some extent, our findings reflect broadly similar patterns that have been reported on heterogeneity and biodiversity for riparian buffers across various tropical agricultural landscapes (Luke et al., 2019b) and specifically for forested buffers in oil palm (Gray et al., 2015; Mitchell et al., 2018). However, in contrast to studies on forested buffers, our findings are less consistent and vary with the environmental condition and taxon being measured, suggesting that this management approach is not currently delivering the full range of environmental benefits that can result from buffer areas.

In mature plantations (i.e., Age M), we found no differences in environmental conditions and arthropod biodiversity between buffer and non‐buffer areas. This suggests that mature palm buffers, managed in a less intensive way than the surrounding plantation (Luke et al., 2020a), provide limited additional benefits within mature oil palm ecosystems. As the only difference between buffer and non‐buffer areas in Age M was the level of herbicide, fertilizer, and pesticide application, this suggests that the amount of chemicals applied under normal (non‐riparian) management within mature plantations has only limited impacts on the environmental conditions and arthropod community, at least at the scale of this study. In contrast, in young plantations (i.e., Ages 1 and 3), which differed from mature palm buffers in terms of structure as well as chemical application, we found instances where environmental conditions and arthropod biodiversity were substantially different between buffer and non‐buffer areas.

Mature palm buffers had lower canopy openness, a ground cover more dominated by ferns, and cooler soil temperatures than non‐buffer areas in Ages 1 and 3. Buffers also had lower variation in openness than non‐buffer areas in Age 1. These differences in environmental conditions almost certainly resulted from the recent replanting of non‐buffer areas in these cohorts, as replanting of oil palm has previously been shown to change vegetation complexity and microclimate substantially (Pashkevich et al., 2021a). In regard to biodiversity, mature palm buffers had more understory arthropods than non‐buffer areas in Ages 3 and 8, and had more understory spiders than non‐buffer areas in Age 1. Also, spider species‐level community composition in all microhabitats varied between buffer and non‐buffer areas across cohorts. However, we also found evidence that buffers could have lower levels of biodiversity than non‐buffer areas, as buffers had fewer understory spiders than Core areas in Age 3. We suggest that these differences in biodiversity are due to differences in environmental conditions between buffer and non‐buffer areas in Ages 1 and 3. For instance, in Age 1, buffers may have had more understory spiders than non‐buffer areas, because the canopy in non‐buffer areas was almost entirely open and spiders are prone to desiccation (Danks, 2002). Buffers may have had fewer understory spiders than non‐buffer areas in Age 3 due to differences in vegetation complexity. Age 3 buffers were dominated by a dense understory, primarily consisting of ferns, which contributed to these buffers having the highest vegetation height of any cohort. It is possible that these ferns were so dense that understory spiders, the majority of which were web‐weavers in the families Araneidae and Tetragnathidae, were less able to build their webs, therefore reducing their abundance in these areas.

Differences in arthropod biodiversity between buffer and non‐buffer areas across the oil palm commercial life cycle could affect functioning, as arthropods facilitate important ecosystem functions within oil palm plantations, including waste management (Gray et al., 2016), pollination (Li et al., 2019), decomposition (Eycott et al., 2019), and pest control (Nurdiansyah et al., 2016). Woodham et al. (2019) previously studied the impacts of mature palm buffers on ecosystem functioning and found few differences in individual functions or multifunctionality between buffer and non‐buffer areas in second‐generation oil palm plantations (Woodham et al., 2019). These findings are congruous with ours, since we found inconsistent differences in arthropod biodiversity across the oil palm commercial life cycle, and it is possible that the differences we observed were not marked enough to alter rates of functioning. Our findings therefore indicate that mature palm buffers that are being passively restored have few impacts on functioning in second‐generation oil palm plantations.

The differences in arthropod biodiversity that we observed between buffer and non‐buffer areas could be indicative of changes in the biodiversity of a wider range of taxonomic groups. We found that mature palm buffers increased habitat heterogeneity in recently replanted oil palm plantations. Previous studies have shown that maintaining heterogeneity in oil palm landscapes can improve the biodiversity and structural complexity of plants (Luke et al., 2019a), and the abundance or biodiversity of a wide range of invertebrate and vertebrate taxa, including birds (Teuscher et al., 2016; Yahya et al., 2017), bats (Syafiq et al., 2016), soil invertebrates (Ashton‐Butt et al., 2018), and leopard cats (Hood et al., 2019). Additionally, as arthropods influence existing trophic networks in oil palm systems (Barnes et al., 2014), and otherwise interact ecologically with non‐arthropod groups (for instance, termite mounds are valuable nesting sites for snakes in oil palm plantations; Hood et al., 2020b), differences in arthropod biodiversity between buffer and non‐buffer areas could affect the biodiversity of non‐arthropod biota. Future studies are needed to determine the impacts of mature palm buffers on non‐arthropod taxonomic groups, and potential knock‐on effects on ecosystem functioning.

### Impacts of mature palm buffers on adjacent edge areas

We found some evidence that mature palm buffers that are being passively restored affect environmental conditions in adjacent non‐buffer areas (i.e., Edge). For example, vegetation height decreased with distance from mature palm buffers and, in comparison to areas far from buffers (i.e., Core), ground cover in Edge was more similar to that within buffers in Age 8. These impacts on environmental conditions could be attributed to buffers contributing to higher levels of plant biodiversity in Edge areas, possibly by acting as sources of seeds or by influencing soil or microclimatic conditions in adjacent areas. However, despite buffers affecting environmental conditions in Edge, we found no evidence for mature palm buffers contributing to higher levels of arthropod biodiversity in Edge areas. This suggests that the impacts of mature palm buffers on environmental conditions in Edge were not sufficient to enhance levels of arthropod biodiversity outside of buffers. These findings are broadly consistent with research on the effects of forested areas within oil palm plantations (Gray et al., 2016; Lucey & Hill, 2012), which found that forested habitat can affect environmental conditions in the adjacent oil palm, but that effects on biodiversity only occur across limited distances (Gray et al., 2016) and are often confined to certain taxonomic groups (Gray et al., 2016; Lucey & Hill, 2012). Similarly, Woodham et al. (2019) found that mature palm buffers do not affect levels of ecosystem functioning in adjacent non‐buffer areas of plantation. Taken together, these findings indicate that areas of greater habitat complexity within oil palm plantations have only limited ability to alter the environmental conditions and biodiversity of the surrounding landscape.

### Changes in mature palm buffers over time

To our knowledge, this is the first study that has examined whether mature palm buffers that are being passively restored become more environmentally complex and biodiverse over time. We found that canopies within buffers steadily opened and became more variable (from Age M to Age 3) before becoming more closed and less variable over time (i.e., Age 8). Opening of buffer canopies could have been caused by pests or diseases. Outbreaks of pests (such as the rhinoceros beetle, *Oryctes rhinoceros*, and moths in the family Psychidae) and diseases (most notably basal stem rot disease, caused by *Ganoderma* fungi) often occur in oil palm plantations and can result in the defoliation or death of palms, causing the oil palm canopy to become more open (Corley & Tinker, 2016). The lower canopy openness and variation in openness that we observed in buffers in Age 8 could be attributed to other vegetation, such as epiphytic figs, growing among palms and closing the canopy. We also found changes in ground cover within buffers over time, with buffers in Ages 1 and 3 having a higher occurrence of ferns than buffers in Ages M and 8. However, these changes were only slight, suggesting that ground cover within buffers changes little, and reinforcing our vegetation height and soil temperature findings, which indicated no changes within buffers over time.

Although environmental conditions changed within the passively restoring buffers over time, we observed no concurrent changes in arthropod biodiversity (total arthropod abundance; arthropod order‐level composition; spider abundance, species richness, and species‐level composition). This could be attributed to the limited changes in ground cover and no change in vegetation height that we observed, since the biodiversity of arthropods, such as spiders, is dependent on variation in vegetation complexity (Greenstone, 1984; Stenchly et al., 2011). However, it should be noted that the oldest mature palm buffers that we sampled were in 8‐year‐old second generation plantations (i.e., Age 8). It is likely that older mature palm buffers exist in other plantations in Southeast Asia, and it is possible that habitat complexity and arthropod biodiversity could improve or decline within these older buffers over time.

## CONCLUSIONS AND MANAGEMENT IMPLICATIONS

In this study, we asked whether (1) environmental conditions and levels of arthropod biodiversity differ between mature oil palm riparian buffers that are being passively restored and surrounding areas of plantation; (2) mature oil palm riparian buffers affect environmental conditions and biodiversity in adjacent non‐buffer areas; and (3) mature palm buffers become more environmentally complex and biodiverse over time? Mature palm buffers occupied ~200 ha of land and represented about 1.36% of all cultivated area within the plantations in which we sampled (not accounting for topographical differences across the landscape), and therefore any environmental or biodiversity benefits that they provide would have occurred at the expense of relatively little cultivated area. Our findings have clear management implications regarding the maintenance of riparian buffers in oil palm landscapes. First, we show that mature palm buffers that are being passively restored (meaning, in this case, that buffers were treated with no herbicides, pesticides, or fertilizers) can increase habitat heterogeneity and benefit biodiversity within oil palm systems and maintain some pre‐replanting environmental conditions and aspects of arthropod biodiversity within recently replanted oil palm landscapes (i.e., Ages 1 and 3). However, the comparative benefit of these passively restored mature palm buffers to the surrounding non‐buffer area varies across the oil palm commercial life cycle. Although maintaining mature palm buffers cannot mitigate the substantial declines in biodiversity (Drescher et al., 2016; Foster et al., 2011) and functioning (Dislich et al., 2017) that occur when forest is converted to oil palm, buffers may offer real environmental benefits within established oil palm systems. We also found that mature palm buffers that are being passively restored have some impacts on environmental conditions in adjacent non‐buffer areas (i.e., Edge), but do not contribute to higher levels of arthropod biodiversity. Lastly, we demonstrate that canopy openness and ground cover change within passively restored mature palm buffers over time, but that buffers do not show increases in arthropod biodiversity, at least over the ~8‐year timeframe represented by the chronosequence in this study. It is possible that, over time, levels of biodiversity within buffers will also increase, as time elapsed since restoration began is a key predictor of restoration success (César et al., 2021; Crouzeilles et al., 2016, 2017). It is noteworthy that we assessed the effects of mature palm buffers on the biodiversity of most arthropod taxa only at the order level. Species‐level analyses may have indicated larger and more consistent benefits of mature palm buffers to arthropod biodiversity across the oil palm commercial life cycle. Other potential benefits of mature palm buffers, such as supporting other taxa, preventing soil erosion, and improving stream water quality (Luke et al., 2019b), could make them valuable to conservation and palm oil production for other reasons, but were beyond the scope of this study.

If the goal of maintaining riparian buffers within oil palm systems is to consistently increase habitat heterogeneity and improve biodiversity in all microhabitats and across the oil palm commercial life cycle, our findings indicate that more active management of mature palm buffers or adjustments to their design are needed. A possible management strategy could be to enrich mature palm buffers by planting forest tree species. This could increase vegetation complexity within buffers and provide resources for a wider range of biodiversity within oil palm plantations. For instance, the EFForTS Biodiversity Enrichment Experiment (EFForTS‐BEE) in Jambi, Indonesia has shown that planting diverse tree islands can increase structural complexity (Zemp et al., 2019a) and bird species richness (Teuscher et al., 2016) in smallholder oil palm plantations. Similarly, other studies in Mexico have shown that planting native tree species can benefit habitat heterogeneity and improve biodiversity within mahogany plantations (Campos‐Navarrete et al., 2015; Esquivel‐Gómez et al., 2017). We suggest that oil palm managers should consider planting forest trees within mature palm buffers several years before the life span (which can be more than a century) of the oil palms within buffers is reached. This will allow native trees to grow among and eventually replace the mature palms, helping to maintain structural and ecological complexity within riparian areas. However, before strategies such as this are carried out across plantations, studies are needed to determine the costliness and effectiveness of such an approach. One such study is the Riparian Ecosystem Restoration in Tropical Agriculture (RERTA) Project, which is currently testing the value of enrichment planting within riparian buffers to biodiversity, ecosystem processes, and yields in replanted oil palm plantations (Luke et al., 2020a). As results from studies such as this become available, it will be possible to identify tractable strategies that maximize the benefits of riparian buffers on the environment and biodiversity within oil palm systems. These strategies can then inform best practice guidance within certification schemes, such as the Roundtable on Sustainable Palm Oil, and ultimately promote more sustainable development of the global palm oil industry.

## CONFLICT OF INTEREST

Co‐authors with a Sinar Mas Agro Resources and Technology Research Institute (SMARTRI) affiliation were employed by SMARTRI, the research division of Golden Agri Resources (GAR), while research was conducted. SMARTRI and the University of Cambridge share a Memorandum of Understanding that protects the intellectual property rights and data‐use for all researchers involved in this study. This research is therefore a collaboration between the University of Cambridge and GAR.

## AUTHOR CONTRIBUTIONS

Michael D. Pashkevich led data collection (with assistance from Anak Agung Ketut Aryawan and Helen S. Waters), spider identification (with assistance from Nadine Dupérré), statistical analyses (with assistance from Helen S. Waters), and writing of the manuscript. Michael D. Pashkevich, Helen S. Waters, Anak Agung Ketut Aryawan, Sarah H. Luke, and ECT designed the study. Michael D. Pashkevich, Anak Agung Ketut Aryawan, Sarah H. Luke, Nadine Dupérré, Mohammad Naim, Jean‐Pierre Caliman, and Edgar C. Turner contributed to the design of terrestrial arthropod sampling and identification protocols. All authors reviewed and approved the manuscript.

## Supporting information


Appendix S1

## Data Availability

Data (Pashkevich et al., 2021b) are available in Apollo, the University of Cambridge repository, at https://doi.org/10.17863/CAM.73877.
