# Supplementary material for: Riparian buffers made of mature oil palms have inconsistent impacts on oil palm ecosystems
Source: Ecol Appl. 2022 Mar 29;32(4):e2552. doi: 10.1002/eap.2552 (PMC9286838; doi:10.1002/eap.2552)
Supplement: Supplementary file 1 — Appendix S1 [file EAP-32-0-s001.pdf]

Riparian buffers made of mature oil palms have inconsistent impacts on oil palm ecosystems

Michael D. Pashkevich, Sarah H. Luke, Anak Agung Ketut Aryawan, Helen S. Waters, Jean-Pierre Caliman, Nadine Dupérré, Mohammad Naim, Anton M. Potapov, Edgar C. Turner

Ecological Applications

Appendix S1

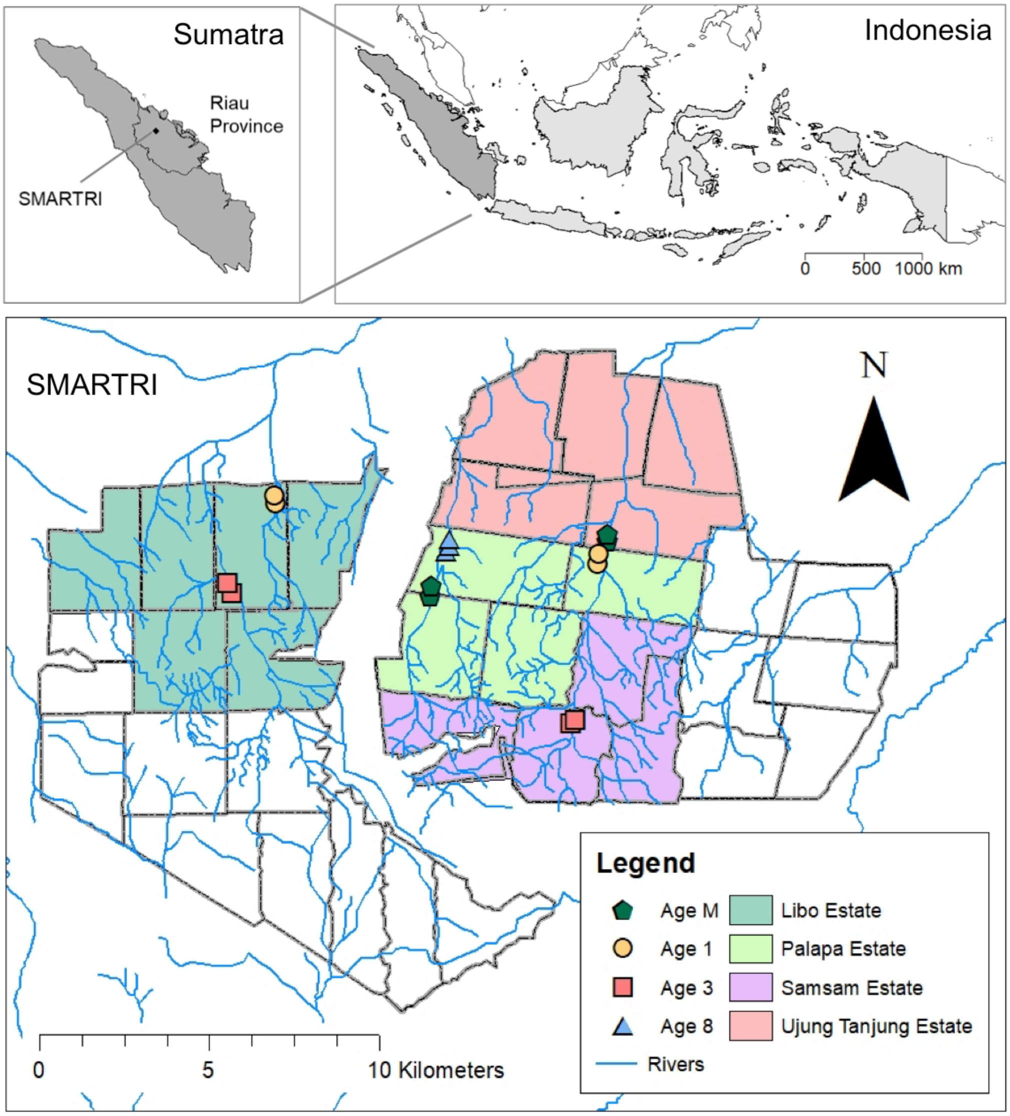

Figure S1. Location of study sites in Riau, Sumatra, Indonesia. Sites were located across four oil palm estates (Libo, Palapa, Samsam, and Ujung Tanjung), which are highlighted in colour, and all sites were near rivers with neighbouring mature palm riparian buffers. Sites were located in four different age cohorts of oil palm, which are indicated by point shape: pentagon (*Age M*), circle (*Age 1*), square (*Age 3*), and triangle (*Age 8*). At each site, we established a triplet of 100 m transects. Transects were located at three distances from riparian buffers (50 m wide from either riverbank): within riparian buffers ("*Buffer*"; 25 m from riverbanks), just outside buffers within the surrounding cropland ("*Edge*"; 75 m from riverbanks), and far from buffers in the core of the plantation landscape ("*Core*"; 175 m from riverbanks). The additional *Age M* sites used in our environmental conditions analyses are not shown but were located in Palapa Estate. Maps were made using ArcMap 10.5.1 (ESRI, 2017) and *maps* (Brownrigg, 2018).

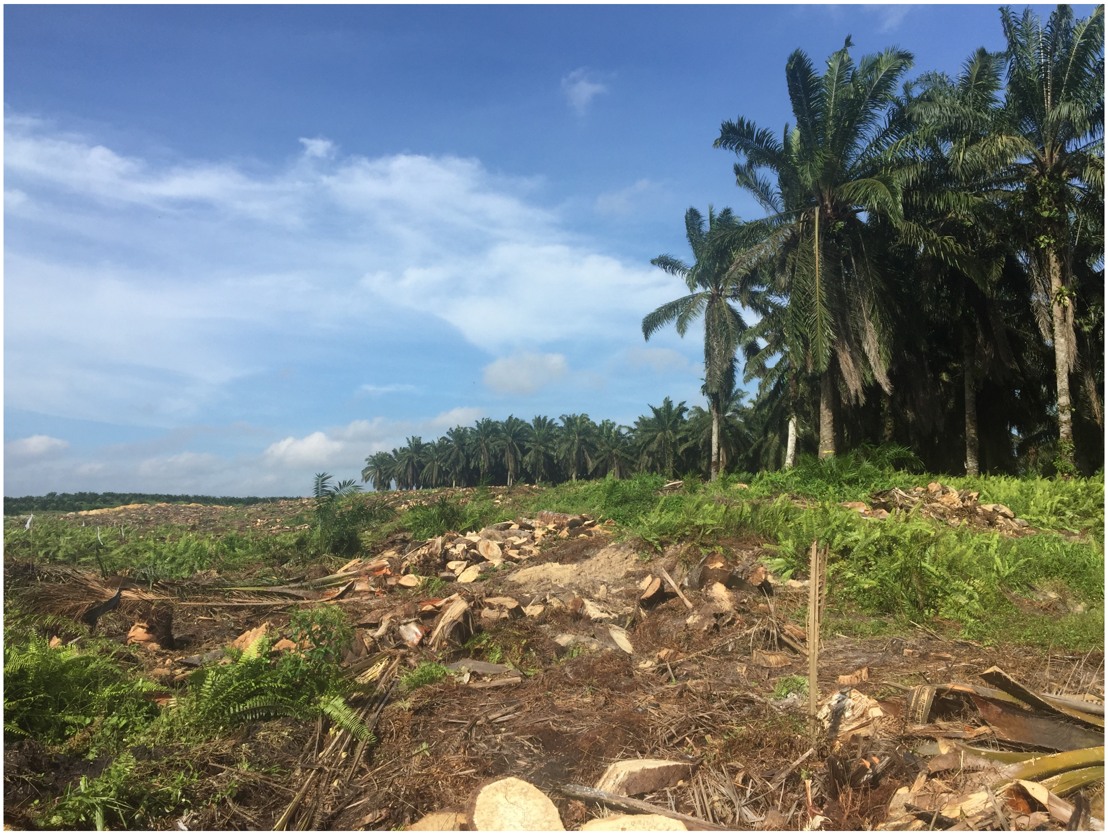

Figure S2. A photograph of a mature oil palm riparian buffer in a plantation that was recently cleared for replanting. In the plantation in which we sampled, mature first-generation oil palms are cleared for replanting using large diggers. The diggers do not enter mature palm buffers, and so these strips of mature oil palm along plantation rivers are left intact. Photo taken by Michael Pashkevich.

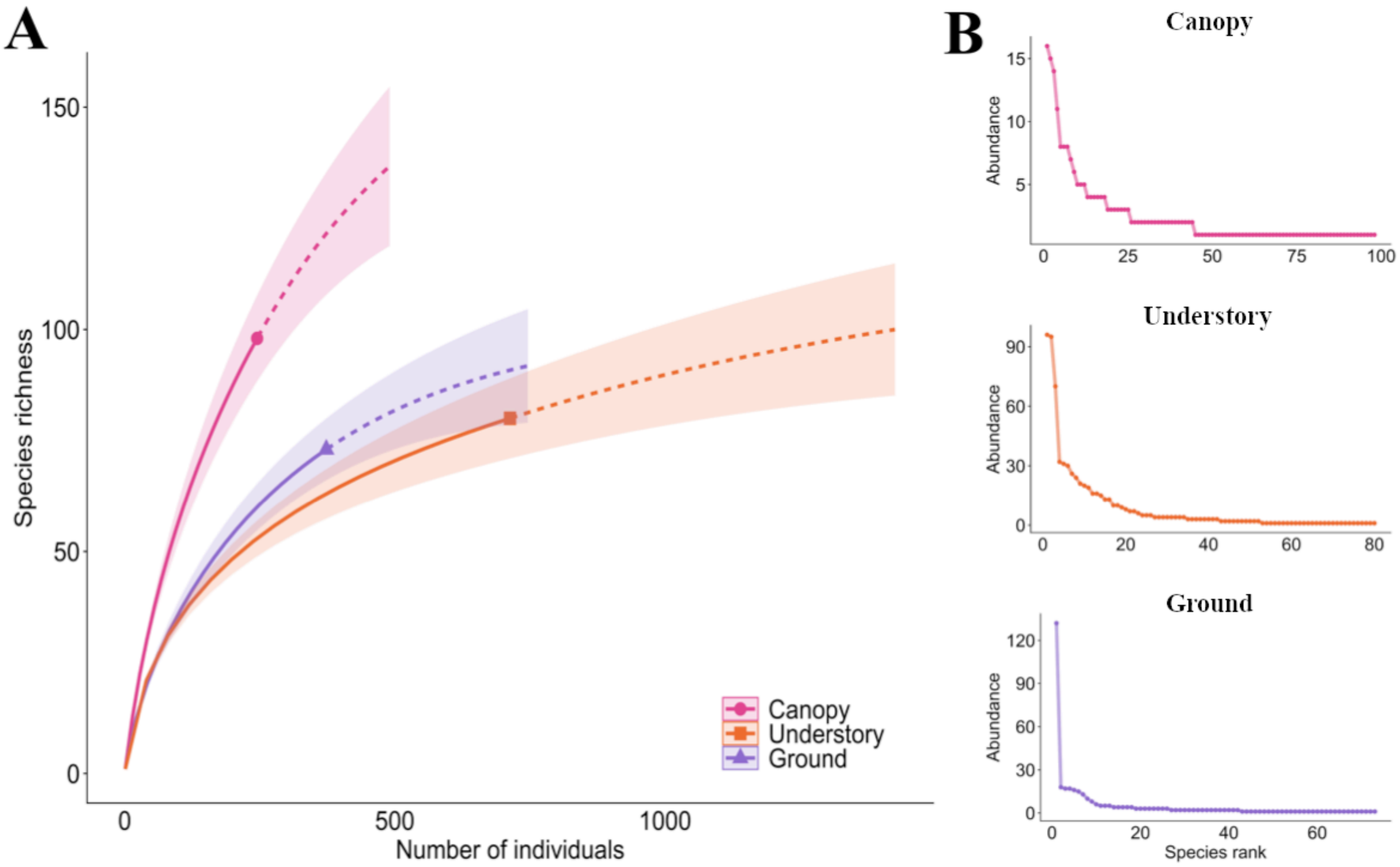

Figure S3. Species accumulation curves (A) and rank abundance curves (B) for the observed spider communities in the canopy, understory, and ground microhabitats. In A, we plot both interpolated (solid lines) and extrapolated (dotted lines) species richness. We extrapolated to double the number of observed individuals (Chao et al., 2014; Gotelli & Colwell, 2001).

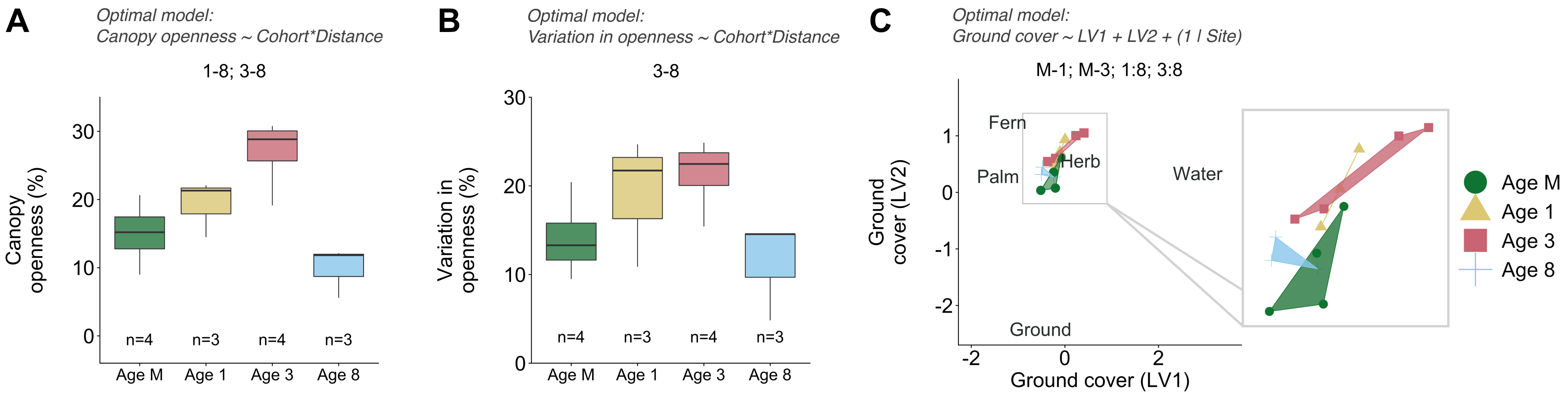

Figure S4. Differences in canopy openness (A), variation in openness (B), and ground cover (C) between *Buffer* areas across cohorts (*Age M*, *Age 1*, *Age 3*, *Age 8*). Posterior distributions from all GLMMs tracked to their underlying datasets. We indicate the optimal model (determined using LOOIC) in text above each subplot. The optimal model for C was not determined using LOOIC, as we did not follow a model selection procedure for our ground cover analyses. Differences between cohorts (as determined by our post-hoc analyses) are indicated in text above each subplot: *Age M (M), Age 1 (1), Age 3 (3) Age 8 (8)*. In A and B, boxplots display the median and interquartile ranges of the data, and lettering below boxplots indicates the number of independent replicates per *Cohort*. In C, points indicate the posterior medians of the latent variables from the pure GLLVM that we fitted to analyse ground cover data (Fern = ferns; Herb = herbaceous plants; Palm = dead and fallen palm fronds; Ground = bare ground; Water = water-filled ditch). Polygons were drawn around outlying points from the same *Cohort* combination, in order to aid visualisation. We magnify polygons in the pop-out window, in order to better show their spatial orientation.

Table S1. Effects of *Cohort* and *Distance* on ***canopy openness, variation in canopy openness, vegetation height, and soil temperature***. For each response, the model with the lowest exact leave-one-out information criterion (LOOIC), and its standard error, is listed first. The difference in expected log pointwise densities (ELPD) and corresponding standard errors is also provided. The optimal model, which is indicated in bold, is that with the lowest LOOIC, unless the standard errors of the difference in ELPD of other models exceeded the difference in ELPD between these models and the model with the lowest LOOIC. In this case, we chose the more parsimonious model. Our ground cover analysis is not included, since we did not use LOOIC to determine the optimal ground cover model. We included the findings of our canopy openness and variation in openness sensitivity analyses, which showed that adding *Site* as a random intercept effect did not improve the fit of these models.

	Model	LOOIC	SE LOOIC	ELPD Difference	SE Difference
Canopy openness	**~ Cohort*Distance**	**105.3**	**15.4**	**0.0**	**0.0**
	~ Cohort*Distance + (1 | Site)	106.2	14.7	-0.5	1.3
	~ Cohort + Distance	117.3	15.1	-6.0	3.8
	~ Cohort + Distance + (1 | Site)	119.3	15.3	-7.0	4.4
	~ Cohort	138.1	17.8	-16.4	5.3
	~ Distance + (1 | Site)	139.0	14.4	-16.9	5.0
	~ Cohort + (1 | Site)	140.2	18.0	-17.5	5.4
	~ 1 + (1 | Site)	159.4	14.7	-27.1	4.6
	~ Distance	166.5	13.0	-30.6	5.1
	~ 1	167.3	13.2	-31.0	4.9
Variation in openness	~ Cohort*Distance + (1 | Site)	82.9	8.6	0.0	0.0
	**~ Cohort*Distance**	**83.3**	**10.2**	**-0.2**	**2.0**
	~ Cohort	96.5	8.6	-6.8	4.6
	~ Cohort + Distance	96.7	8.0	-6.9	3.7
	~ Cohort + (1 | Site)	98.5	8.7	-7.8	4.9
	~ Cohort + Distance + (1 | Site)	98.7	7.7	-7.9	3.9
	~ 1 + (1 | Site)	108.5	7.9	-12.8	5.4
	~ Distance + (1 | Site)	109.0	7.6	-13.1	4.8
	~ 1	117.6	7.5	-17.3	5.1
	~ Distance	119.5	7.9	-18.3	5.0
Vegetation height	~ Cohort + Distance + (1 | Site)	29.8	9.0	0.0	0.0
	**~ Distance + (1 | Site)**	**31.3**	**7.6**	**-0.8**	**1.8**
	~ Cohort*Distance + (1 | Site)	34.1	9.0	-2.2	2.5
	~ Cohort + (1 | Site)	35.9	8.2	-3.1	2.9
	~ 1 + (1 | Site)	37.5	7.5	-3.8	3.7
Soil temperature	**~ Cohort + Distance + s(Time, by = Cohort) + s(Time, by = Distance) + (1 | Site)**	**364.5**	**22.1**	**0.0**	**0.0**
	~ Cohort + Distance + s(Time, by = interaction(Cohort, Distance)) + (1 | Site)	377.2	18.7	-6.4	5.4
	~ Cohort + s(Time, by = Cohort) + (1 | Site)	386.6	25.2	-11.1	6.4
	~ Distance + s(Time, by = Distance) + (1 | Site)	400.6	28.7	-18.1	9.1
	~ 1 +s(Time) + (1 | Site)	408.4	32.0	-22.0	11.2

Table S2. Model summary tables for GLMM analyses used to explain differences in ***canopy openness*** across the chronosequence. We present model estimates and associated standard errors and 95% credible intervals for each fixed parameter and the beta-binomial phi parameter. In all models, we specified *Age M-Buffer* as the intercept (Cohort: *Age M*, *Age 1*, *Age 3*, *Age 8;* Distance: *Buffer*, *Edge*, *Core*).

Canopy openness ~ Cohort*Distance

Parameter	*Estimate*	*SE*	*95% CI*
Intercept	-3.18	0.84	-5.00 - -1.71
Age1	1.26	1.04	-0.75 - 3.36
Age3	1.43	0.98	-0.41 - 3.45
Age8	-3.98	2.50	-9.44 - 0.24
Edge	1.89	0.98	0.07 - 3.90
Core	0.51	1.15	-1.84 - 2.75
Age1:Edge	7.01	3.01	2.19 - 13.83
Age3:Edge	1.46	1.23	-0.98 - 3.87
Age8:Edge	-2.00	3.44	-9.23 - 4.43
Age1:Core	7.94	2.86	3.25 - 14.42
Age3:Core	2.83	1.35	0.23 - 5.58
Age8:Core	-1.15	3.59	-8.72 - 5.48
phi	12.79	15.50	2.74 - 47.38

Canopy openness ~ Cohort + Distance

Parameter	*Estimate*	*SE*	*95% CI*
Intercept	-4.82	0.86	-6.43 - -3.07
Age1	4.05	0.73	2.62 - 5.50
Age3	3.00	0.65	1.69 - 4.25
Age8	-3.81	1.70	-7.74 - -1.13
Edge	3.63	0.76	2.12 - 5.10
Core	3.18	0.73	1.68 - 4.58
phi	6.56	7.61	1.52 - 21.15

Canopy openness ~ Cohort

Parameter	*Estimate*	*SE*	*95% CI*
Intercept	-1.47	0.48	-2.45 - -0.56
Age1	3.07	0.85	1.48 - 4.83
Age3	1.76	0.64	0.52 - 3.04
Age8	-3.80	1.90	-8.22 - -0.81
phi	1.00	0.32	0.50 - 1.76

Canopy openness ~ Distance

Parameter	*Estimate*	*SE*	*95% CI*
Intercept	-1.34	0.46	-2.28 - -0.47
Edge	1.38	0.64	0.15 - 2.65
Core	1.05	0.65	-0.22 - 2.33
phi	0.57	0.17	0.30 - 0.96

Canopy openness ~ 1

Parameter	*Estimate*	*SE*	*95% CI*
Intercept	-0.53	0.25	-1.02 - -0.04
phi	0.48	0.14	0.26 - 0.79

Table S3. Pairwise comparisons of estimated marginal means between factor levels in the optimal ***canopy openness*** model: *Canopy openness ~ Cohort*Distance*. We concluded that factor levels were meaningfully different if the 95% highest posterior density (HPD) interval of the median point estimate calculated from our comparisons did not overlap with zero (indicated in bold). Results are given on the log odds ratio scale and with a HPD interval probability of 0.95.

Comparison			Lower HPD	Upper HPD	Estimate
Age M-Buffer	---	Age 1-Buffer	-3.335	0.774	-1.242
Age M-Buffer	---	Age 3-Buffer	-3.375	0.483	-1.401
Age M-Buffer	---	Age 8-Buffer	-0.548	8.962	3.759
Age M-Buffer	---	Age M-Edge	-3.830	0.000	-1.857
**Age M-Buffer**	**---**	**Age 1-Edge**	**-16.574**	**-4.884**	**-9.708**
**Age M-Buffer**	**---**	**Age 3-Edge**	**-6.886**	**-2.797**	**-4.734**
Age M-Buffer	---	Age 8-Edge	-1.433	10.711	3.601
Age M-Buffer	---	Age M-Core	-2.825	1.747	-0.530
**Age M-Buffer**	**---**	**Age 1-Core**	**-15.705**	**-4.716**	**-9.268**
**Age M-Buffer**	**---**	**Age 3-Core**	**-6.815**	**-2.729**	**-4.728**
Age M-Buffer	---	Age 8-Core	-1.183	11.821	4.136
Age 1-Buffer	---	Age 3-Buffer	-1.953	1.576	-0.166
**Age 1-Buffer**	**---**	**Age 8-Buffer**	**0.542**	**10.383**	**5.003**
Age 1-Buffer	---	Age M-Edge	-2.383	1.138	-0.623
**Age 1-Buffer**	**---**	**Age 1-Edge**	**-15.017**	**-3.924**	**-8.474**
**Age 1-Buffer**	**---**	**Age 3-Edge**	**-5.393**	**-1.714**	**-3.497**
**Age 1-Buffer**	**---**	**Age 8-Edge**	**0.031**	**11.814**	**4.848**
Age 1-Buffer	---	Age M-Core	-1.436	2.994	0.693
**Age 1-Buffer**	**---**	**Age 1-Core**	**-14.184**	**-3.780**	**-8.031**
**Age 1-Buffer**	**---**	**Age 3-Core**	**-5.352**	**-1.708**	**-3.494**
**Age 1-Buffer**	**---**	**Age 8-Core**	**0.179**	**12.864**	**5.375**
**Age 3-Buffer**	**---**	**Age 8-Buffer**	**0.707**	**10.434**	**5.175**
Age 3-Buffer	---	Age M-Edge	-2.050	1.098	-0.458
**Age 3-Buffer**	**---**	**Age 1-Edge**	**-14.835**	**-3.735**	**-8.268**
**Age 3-Buffer**	**---**	**Age 3-Edge**	**-5.014**	**-1.733**	**-3.326**
**Age 3-Buffer**	**---**	**Age 8-Edge**	**0.294**	**11.923**	**5.025**
Age 3-Buffer	---	Age M-Core	-1.078	3.051	0.861
**Age 3-Buffer**	**---**	**Age 1-Core**	**-13.992**	**-3.566**	**-7.829**
**Age 3-Buffer**	**---**	**Age 3-Core**	**-5.001**	**-1.746**	**-3.327**
**Age 3-Buffer**	**---**	**Age 8-Core**	**0.303**	**12.827**	**5.543**
**Age 8-Buffer**	**---**	**Age M-Edge**	**-10.987**	**-1.209**	**-5.631**
**Age 8-Buffer**	**---**	**Age 1-Edge**	**-22.067**	**-6.857**	**-13.723**
**Age 8-Buffer**	**---**	**Age 3-Edge**	**-13.897**	**-4.098**	**-8.535**
Age 8-Buffer	---	Age 8-Edge	-6.627	7.182	-0.036
Age 8-Buffer	---	Age M-Core	-9.966	0.398	-4.269
**Age 8-Buffer**	**---**	**Age 1-Core**	**-21.424**	**-6.776**	**-13.281**
**Age 8-Buffer**	**---**	**Age 3-Core**	**-13.963**	**-4.138**	**-8.505**
Age 8-Buffer	---	Age 8-Core	-6.344	7.953	0.452
**Age M-Edge**	**---**	**Age 1-Edge**	**-14.210**	**-3.300**	**-7.815**
**Age M-Edge**	**---**	**Age 3-Edge**	**-4.508**	**-1.342**	**-2.858**
**Age M-Edge**	**---**	**Age 8-Edge**	**0.776**	**12.307**	**5.481**
Age M-Edge	---	Age M-Core	-0.616	3.390	1.319
**Age M-Edge**	**---**	**Age 1-Core**	**-13.511**	**-3.152**	**-7.365**
**Age M-Edge**	**---**	**Age 3-Core**	**-4.453**	**-1.350**	**-2.865**
**Age M-Edge**	**---**	**Age 8-Core**	**0.862**	**13.377**	**6.009**
**Age 1-Edge**	**---**	**Age 3-Edge**	**0.489**	**11.560**	**4.911**
**Age 1-Edge**	**---**	**Age 8-Edge**	**6.471**	**22.852**	**13.756**
**Age 1-Edge**	**---**	**Age M-Core**	**4.404**	**15.800**	**9.196**
Age 1-Edge	---	Age 1-Core	-7.567	8.619	0.417
**Age 1-Edge**	**---**	**Age 3-Core**	**0.319**	**11.349**	**4.925**
**Age 1-Edge**	**---**	**Age 8-Core**	**6.615**	**23.808**	**14.264**
**Age 3-Edge**	**---**	**Age 8-Edge**	**3.704**	**15.369**	**8.377**
**Age 3-Edge**	**---**	**Age M-Core**	**2.315**	**6.416**	**4.199**
**Age 3-Edge**	**---**	**Age 1-Core**	**-10.655**	**-0.238**	**-4.479**
Age 3-Edge	---	Age 3-Core	-1.609	1.584	-0.007
**Age 3-Edge**	**---**	**Age 8-Core**	**3.775**	**16.322**	**8.899**
Age 8-Edge	---	Age M-Core	-11.253	0.977	-4.116
**Age 8-Edge**	**---**	**Age 1-Core**	**-22.206**	**-6.355**	**-13.274**
**Age 8-Edge**	**---**	**Age 3-Core**	**-15.261**	**-3.620**	**-8.364**
Age 8-Edge	---	Age 8-Core	-8.440	9.466	0.480
**Age M-Core**	**---**	**Age 1-Core**	**-14.924**	**-4.394**	**-8.787**
**Age M-Core**	**---**	**Age 3-Core**	**-6.304**	**-2.278**	**-4.200**
Age M-Core	---	Age 8-Core	-0.685	12.032	4.643
**Age 1-Core**	**---**	**Age 3-Core**	**0.305**	**10.589**	**4.482**
**Age 1-Core**	**---**	**Age 8-Core**	**6.507**	**23.111**	**13.806**
**Age 3-Core**	**---**	**Age 8-Core**	**3.695**	**16.219**	**8.878**

Table S4. Model summary tables for GLMM analyses used to explain differences in ***variation in canopy openness*** across the chronosequence. We present model estimates and associated standard errors and 95% credible intervals for each fixed parameter and the Gaussian sigma parameter. In all models, we specified *Age M-Buffer* as the intercept (Cohort: *Age M*, *Age 1*, *Age 3*, *Age 8;* Distance: *Buffer*, *Edge*, *Core*).

Variation in openness ~ Cohort*Distance

Parameter	*Estimate*	*SE*	*95% CI*
Intercept	-1.52	0.23	-1.96 - -1.07
Age1	-0.15	0.35	-0.85 - 0.52
Age3	0.33	0.32	-0.31 - 0.95
Age8	-0.46	0.35	-1.14 - 0.22
Edge	0.02	0.31	-0.60 - 0.63
Core	-0.31	0.31	-0.93 - 0.30
Age1:Edge	-1.82	0.49	-2.75 - -0.82
Age3:Edge	0.30	0.45	-0.57 - 1.17
Age8:Edge	-0.34	0.48	-1.27 - 0.61
Age1:Core	-0.91	0.48	-1.84 - 0.06
Age3:Core	0.52	0.45	-0.36 - 1.40
Age8:Core	-0.44	0.48	-1.38 - 0.51
sigma	0.58	0.08	0.45 - 0.75

Variation in openness ~ Cohort + Distance

Parameter	*Estimate*	*SE*	*95% CI*
Intercept	-1.40	0.25	-1.89 - -0.92
Age1	-0.97	0.30	-1.55 - -0.38
Age3	0.62	0.28	0.07 - 1.17
Age8	-0.66	0.30	-1.25 - -0.07
Edge	-0.32	0.26	-0.83 - 0.19
Core	-0.43	0.26	-0.94 - 0.09
sigma	0.72	0.09	0.57 - 0.92

Variation in openness ~ Cohort

Parameter	*Estimate*	*SE*	*95% CI*
Intercept	-1.65	0.20	-2.05 - -1.26
Age1	-0.97	0.30	-1.57 - -0.37
Age3	0.62	0.28	0.07 - 1.18
Age8	-0.66	0.30	-1.26 - -0.06
sigma	0.73	0.09	0.59 - 0.93

Variation in openness ~ Distance

Parameter	*Estimate*	*SE*	*95% CI*
Intercept	-1.60	0.25	-2.08 - -1.11
Edge	-0.29	0.34	-0.96 - 0.38
Core	-0.39	0.34	-1.06 - 0.28
sigma	0.98	0.12	0.79 - 1.24

Variation in openness ~ 1

Parameter	*Estimate*	*SE*	*95% CI*
Intercept	-1.83	0.15	-2.13 - -1.53
sigma	0.98	0.11	0.79 - 1.23

Table S5. Pairwise comparisons of estimated marginal means between factor levels in the optimal ***variation in canopy openness*** model: *Variation in openness ~ Cohort*Distance*. We concluded that factor levels were meaningfully different if the 95% highest posterior density (HPD) interval of the median point estimate calculated from our comparisons did not overlap with zero (indicated in bold). Results are given on the response scale and with a HPD interval probability of 0.95.

Comparison			Lower HPD	Upper HPD	Estimate
Age M-Buffer	---	Age 1-Buffer	-0.533	0.831	0.146
Age M-Buffer	---	Age 3-Buffer	-0.960	0.298	-0.329
Age M-Buffer	---	Age 8-Buffer	-0.229	1.132	0.456
Age M-Buffer	---	Age M-Edge	-0.620	0.604	-0.025
**Age M-Buffer**	**---**	**Age 1-Edge**	**1.172**	**2.742**	**1.948**
Age M-Buffer	---	Age 3-Edge	-1.367	0.073	-0.648
Age M-Buffer	---	Age 8-Edge	-0.007	1.563	0.774
Age M-Buffer	---	Age M-Core	-0.308	0.918	0.311
**Age M-Buffer**	**---**	**Age 1-Core**	**0.601**	**2.167**	**1.382**
Age M-Buffer	---	Age 3-Core	-1.267	0.182	-0.542
**Age M-Buffer**	**---**	**Age 8-Core**	**0.428**	**2.000**	**1.213**
Age 1-Buffer	---	Age 3-Buffer	-1.253	0.294	-0.475
Age 1-Buffer	---	Age 8-Buffer	-0.515	1.115	0.310
Age 1-Buffer	---	Age M-Edge	-0.966	0.609	-0.172
**Age 1-Buffer**	**---**	**Age 1-Edge**	**0.942**	**2.623**	**1.802**
**Age 1-Buffer**	**---**	**Age 3-Edge**	**-1.626**	**-0.012**	**-0.795**
Age 1-Buffer	---	Age 8-Edge	-0.234	1.504	0.630
Age 1-Buffer	---	Age M-Core	-0.648	0.935	0.164
**Age 1-Buffer**	**---**	**Age 1-Core**	**0.406**	**2.067**	**1.236**
Age 1-Buffer	---	Age 3-Core	-1.507	0.113	-0.687
**Age 1-Buffer**	**---**	**Age 8-Core**	**0.173**	**1.917**	**1.069**
**Age 3-Buffer**	**---**	**Age 8-Buffer**	**0.024**	**1.552**	**0.785**
Age 3-Buffer	---	Age M-Edge	-0.425	1.054	0.303
**Age 3-Buffer**	**---**	**Age 1-Edge**	**1.437**	**3.077**	**2.276**
Age 3-Buffer	---	Age 3-Edge	-1.045	0.414	-0.322
**Age 3-Buffer**	**---**	**Age 8-Edge**	**0.286**	**1.920**	**1.103**
Age 3-Buffer	---	Age M-Core	-0.111	1.371	0.640
**Age 3-Buffer**	**---**	**Age 1-Core**	**0.894**	**2.533**	**1.710**
Age 3-Buffer	---	Age 3-Core	-0.948	0.519	-0.212
**Age 3-Buffer**	**---**	**Age 8-Core**	**0.734**	**2.373**	**1.543**
Age 8-Buffer	---	Age M-Edge	-1.259	0.324	-0.482
**Age 8-Buffer**	**---**	**Age 1-Edge**	**0.613**	**2.350**	**1.492**
**Age 8-Buffer**	**---**	**Age 3-Edge**	**-1.901**	**-0.294**	**-1.105**
Age 8-Buffer	---	Age 8-Edge	-0.512	1.129	0.318
Age 8-Buffer	---	Age M-Core	-0.937	0.648	-0.145
**Age 8-Buffer**	**---**	**Age 1-Core**	**0.074**	**1.800**	**0.922**
**Age 8-Buffer**	**---**	**Age 3-Core**	**-1.808**	**-0.185**	**-0.996**
Age 8-Buffer	---	Age 8-Core	-0.058	1.589	0.758
**Age M-Edge**	**---**	**Age 1-Edge**	**1.159**	**2.730**	**1.976**
Age M-Edge	---	Age 3-Edge	-1.356	0.095	-0.624
**Age M-Edge**	**---**	**Age 8-Edge**	**0.010**	**1.575**	**0.803**
Age M-Edge	---	Age M-Core	-0.363	1.039	0.336
**Age M-Edge**	**---**	**Age 1-Core**	**0.581**	**2.213**	**1.406**
Age M-Edge	---	Age 3-Core	-1.271	0.234	-0.514
**Age M-Edge**	**---**	**Age 8-Core**	**0.408**	**2.039**	**1.240**
**Age 1-Edge**	**---**	**Age 3-Edge**	**-3.429**	**-1.758**	**-2.599**
**Age 1-Edge**	**---**	**Age 8-Edge**	**-2.063**	**-0.286**	**-1.173**
**Age 1-Edge**	**---**	**Age M-Core**	**-2.445**	**-0.808**	**-1.636**
Age 1-Edge	---	Age 1-Core	-1.435	0.325	-0.569
**Age 1-Edge**	**---**	**Age 3-Core**	**-3.327**	**-1.640**	**-2.491**
Age 1-Edge	---	Age 8-Core	-1.616	0.166	-0.737
**Age 3-Edge**	**---**	**Age 8-Edge**	**0.571**	**2.240**	**1.424**
**Age 3-Edge**	**---**	**Age M-Core**	**0.206**	**1.712**	**0.960**
**Age 3-Edge**	**---**	**Age 1-Core**	**1.200**	**2.860**	**2.030**
Age 3-Edge	---	Age 3-Core	-0.657	0.894	0.106
**Age 3-Edge**	**---**	**Age 8-Core**	**1.020**	**2.687**	**1.862**
Age 8-Edge	---	Age M-Core	-1.293	0.334	-0.465
Age 8-Edge	---	Age 1-Core	-0.291	1.487	0.606
**Age 8-Edge**	**---**	**Age 3-Core**	**-2.130**	**-0.465**	**-1.319**
Age 8-Edge	---	Age 8-Core	-0.449	1.322	0.438
**Age M-Core**	**---**	**Age 1-Core**	**0.276**	**1.841**	**1.069**
**Age M-Core**	**---**	**Age 3-Core**	**-1.568**	**-0.112**	**-0.853**
**Age M-Core**	**---**	**Age 8-Core**	**0.134**	**1.697**	**0.902**
**Age 1-Core**	**---**	**Age 3-Core**	**-2.735**	**-1.072**	**-1.920**
Age 1-Core	---	Age 8-Core	-1.029	0.740	-0.168
**Age 3-Core**	**---**	**Age 8-Core**	**0.917**	**2.587**	**1.757**

Table S6. Model summary tables for GLMM analyses used to explain differences in ***vegetation height*** across the chronosequence. We present model estimates and associated standard errors and 95% credible intervals for each fixed parameter and the Gaussian sigma parameter. In all models, we specified *Age M-Buffer* as the intercept (Cohort: *Age M*, *Age 1*, *Age 3*, *Age 8;* Distance: *Buffer*, *Edge*, *Core*).

Vegetation height ~ Cohort*Distance + (1 | Site)

Parameter	*Estimate*	*SE*	*95% CI*
Intercept	3.39	0.16	3.09 - 3.71
Age1	0.23	0.24	-0.25 - 0.70
Age3	0.53	0.22	0.09 - 0.97
Age8	0.02	0.24	-0.46 - 0.49
Edge	0.14	0.18	-0.23 - 0.50
Core	-0.15	0.18	-0.52 - 0.21
Age1:Edge	-0.33	0.28	-0.87 - 0.24
Age3:Edge	-0.59	0.26	-1.09 - -0.06
Age8:Edge	-0.41	0.28	-0.96 - 0.16
Age1:Core	-0.02	0.28	-0.57 - 0.54
Age3:Core	-0.42	0.26	-0.92 - 0.11
Age8:Core	-0.36	0.28	-0.92 - 0.20
sigma	0.28	0.05	0.21 - 0.39

Vegetation height ~ Cohort + Distance + (1 | Site)

Parameter	*Estimate*	*SE*	*95% CI*
Intercept	3.56	0.14	3.28 - 3.83
Age1	0.13	0.18	-0.24 - 0.49
Age3	0.21	0.17	-0.13 - 0.55
Age8	-0.22	0.18	-0.59 - 0.14
Edge	-0.18	0.11	-0.40 - 0.05
Core	-0.35	0.11	-0.57 - -0.12
sigma	0.30	0.04	0.23 - 0.39

Vegetation height ~ Cohort + (1 | Site)

Parameter	*Estimate*	*SE*	*95% CI*
Intercept	3.38	0.12	3.14 - 3.63
Age1	0.13	0.19	-0.25 - 0.50
Age3	0.21	0.17	-0.14 - 0.56
Age8	-0.23	0.19	-0.60 - 0.15
sigma	0.34	0.04	0.26 - 0.43

Vegetation height ~ Distance + (1 | Site)

Parameter	*Estimate*	*SE*	*95% CI*
Intercept	3.60	0.10	3.40 - 3.79
Edge	-0.18	0.12	-0.40 - 0.05
Core	-0.35	0.12	-0.57 - -0.12
sigma	0.30	0.05	0.23 - 0.40

Vegetation height ~ 1 + (1 | Site)

Parameter	*Estimate*	*SE*	*95% CI*
Intercept	3.42	0.08	3.27 - 3.57
sigma	0.34	0.05	0.27 - 0.45

Table S7. Pairwise comparisons of estimated marginal means between factor levels in the optimal ***vegetation height*** model: *Vegetation height ~ Distance + (1 | Site)*. We concluded that factor levels were meaningfully different if the 95% highest posterior density (HPD) interval of the median point estimate calculated from our comparisons did not overlap with zero (indicated in bold). Results are given on the response scale and with a HPD interval probability of 0.95.

Comparison			Lower HPD	Upper HPD	Estimate
Buffer	---	Edge	-0.047	0.408	0.179
**Buffer**	**---**	**Core**	**0.120**	**0.573**	**0.347**
Edge	---	Core	-0.059	0.399	0.168

Table S8. Model summary tables for GLMM analyses used to explain differences in ***soil temperature*** across the chronosequence. We present model estimates and associated standard errors and 95% credible intervals for each fixed parameter, smooth terms, and the Gaussian sigma parameter. In all models, we specified *Age M-Buffer* as the intercept (Cohort: *Age M*, *Age 1*, *Age 3*, *Age 8;* Distance: *Buffer*, *Edge*, *Core*).

Temperature ~ Cohort + Distance + s(Time, by = interaction(Cohort*Distance)) + (1 | Site)

Parameter	*Estimate*	*SE*	*95% CI*
Intercept	26.55	0.29	25.97 - 27.12
Age1	0.48	0.44	-0.42 - 1.34
Age3	0.02	0.40	-0.78 - 0.81
Age8	-0.36	0.40	-1.15 - 0.44
Core	0.28	0.09	0.10 - 0.46
sds(Time:AgeM.Buffer)	0.16	0.09	0.06 - 0.39
sds(Time:Age1.Buffer)	0.2	0.14	0.04 - 0.55
sds(Time:Age3.Buffer)	0.25	0.12	0.1 - 0.56
sds(Time:Age8.Buffer)	0.17	0.10	0.05 - 0.43
sds(Time:AgeM.Core)	0.19	0.10	0.08 - 0.45
sds(Time:Age1.Core)	0.88	0.30	0.41 - 1.60
sds(Time:Age3.Core)	0.37	0.16	0.17 - 0.76
sds(Time:Age8.Core)	0.20	0.10	0.07 - 0.47
sigma	0.60	0.04	0.53 - 0.69

Temperature ~ Cohort + Distance + s(Time, by = Cohort) + s(Time, by = Distance) + (1 | Site)

Parameter	*Estimate*	*SE*	*95% CI*
Intercept	26.55	0.29	25.98 - 27.13
Age1	0.48	0.44	-0.42 - 1.34
Age3	0.02	0.40	-0.78 - 0.81
Age8	-0.36	0.40	-1.15 - 0.44
RiverDistCore	0.28	0.09	0.10 - 0.46
sds(Time:AgeM)	0.1	0.07	0.01 - 0.28
sds(Time:Age1)	0.58	0.25	0.19 - 1.17
sds(Time:Age3)	0.23	0.12	0.07 - 0.51
sds(Time:Age8)	0.09	0.08	0.00 - 0.29
sds(Time:Buffer)	0.10	0.07	0.01 - 0.27
sds(Time:Core)	0.22	0.12	0.06 - 0.51
sigma	0.60	0.04	0.54 - 0.68

Temperature ~ Cohort + s(Time, by = Cohort) + (1 | Site)

Parameter	*Estimate*	*SE*	*95% CI*
Intercept	26.69	0.28	26.12 - 27.25
Age1	0.49	0.44	-0.41 - 1.34
Age3	0.02	0.40	-0.77 - 0.81
Age8	-0.36	0.40	-1.14 - 0.44
sds(Time:AgeM)	0.17	0.08	0.07 - 0.37
sds(Time:Age1)	0.65	0.25	0.28 - 1.25
sds(Time:Age3)	0.31	0.13	0.15 - 0.63
sds(Time:Age8)	0.19	0.1	0.07 - 0.44
sigma	0.65	0.04	0.58 - 0.73

Temperature ~ Distance + s(Time, by = Distance) + (1 | Site)

Parameter	*Estimate*	*SE*	*95% CI*
Intercept	26.54	0.19	26.16 - 26.91
Core	0.27	0.11	0.06 - 0.47
sds(Time:Buffer)	0.17	0.08	0.07 - 0.37
sds(Time:Core)	0.32	0.13	0.14 - 0.66
sigma	0.70	0.04	0.63 - 0.79

Temperature ~ 1 + s(Time) + (1 | Site)

Parameter	*Estimate*	*SE*	*95% CI*
Intercept	26.67	0.18	26.30 - 27.02
sds(Time)	0.26	0.11	0.12 - 0.54
sigma	0.73	0.04	0.66 - 0.82

Table S9. Pairwise comparisons of estimated marginal means between factor levels in the optimal ***soil temperature*** model: *Temperature ~ Cohort + Distance + s(Time, by = Cohort) + s(Time, by = Distance) + (1 | Site)*. We concluded that factor levels were meaningfully different if the 95% highest posterior density (HPD) interval of the median point estimate calculated from our comparisons did not overlap with zero (indicated in bold). Results are given on the response scale and with a HPD interval probability of 0.95.

Comparison			Lower HPD	Upper HPD	Estimate
Age M-Buffer	---	Age 1-Buffer	-1.370	0.381	-0.488
Age M-Buffer	---	Age 3-Buffer	-0.818	0.772	-0.021
Age M-Buffer	---	Age 8-Buffer	-0.453	1.144	0.367
**Age M-Buffer**	**---**	**Age M-Core**	**-0.458**	**-0.099**	**-0.277**
Age M-Buffer	---	Age 1-Core	-1.635	0.155	-0.762
Age M-Buffer	---	Age 3-Core	-1.128	0.505	-0.296
Age M-Buffer	---	Age 8-Core	-0.727	0.902	0.090
Age 1-Buffer	---	Age 3-Buffer	-0.510	1.425	0.466
Age 1-Buffer	---	Age 8-Buffer	-0.167	1.773	0.854
Age 1-Buffer	---	Age M-Core	-0.692	1.095	0.212
**Age 1-Buffer**	**---**	**Age 1-Core**	**-0.458**	**-0.099**	**-0.277**
Age 1-Buffer	---	Age 3-Core	-0.795	1.171	0.190
Age 1-Buffer	---	Age 8-Core	-0.423	1.544	0.578
Age 3-Buffer	---	Age 8-Buffer	-0.496	1.253	0.388
Age 3-Buffer	---	Age M-Core	-1.067	0.562	-0.257
Age 3-Buffer	---	Age 1-Core	-1.695	0.275	-0.744
**Age 3-Buffer**	**---**	**Age 3-Core**	**-0.458**	**-0.099**	**-0.277**
Age 3-Buffer	---	Age 8-Core	-0.805	0.988	0.111
Age 8-Buffer	---	Age M-Core	-1.467	0.163	-0.643
**Age 8-Buffer**	**---**	**Age 1-Core**	**-2.091**	**-0.119**	**-1.131**
Age 8-Buffer	---	Age 3-Core	-1.546	0.237	-0.664
**Age 8-Buffer**	**---**	**Age 8-Core**	**-0.458**	**-0.099**	**-0.277**
Age M-Core	---	Age 1-Core	-1.370	0.381	-0.488
Age M-Core	---	Age 3-Core	-0.818	0.772	-0.021
Age M-Core	---	Age 8-Core	-0.453	1.144	0.367
Age 1-Core	---	Age 3-Core	-0.510	1.425	0.466
Age 1-Core	---	Age 8-Core	-0.167	1.773	0.854
Age 3-Core	---	Age 8-Core	-0.496	1.253	0.388

Table S10. Arthropods (mean abundance per palm, with standard errors in brackets) that were collected in the ***canopy microhabitat***. All arthropod groups were used in the total abundance analysis. All but Endopterygote larvae and arthropods that we could not identify to order or other group were also used in the order-level community composition analysis.

Canopy arthropods: Abundance per palm (Standard error)

	Age M			Age 1			Age 3			Age 8		
**Order**	**Buffer**	**Edge**	**Core**	**Buffer**	**Edge**	**Core**	**Buffer**	**Edge**	**Core**	**Buffer**	**Edge**	**Core**
**Acari**	13.25 (7.15)	8.25 (5.54)	3.25 (1.65)	5.25 (2.36)	17.00 (10.11)	23.75 (21.09)	14.50 (9.90)	41.25 (21.82)	34.75 (21.61)	15.33 (5.78)	12.33 (6.33)	47.00 (33.13)
**Araneae**	22.50 (11.24)	18.25 (11.17)	12.25 (5.12)	8.25 (1.11)	23.50 (2.47)	30.00 (6.67)	12.75 (10.13)	16.25 (4.94)	22.75 (5.41)	35.33 (9.35)	29.33 (8.21)	65.33 (18.67)
**Blattodea**	4.00 (2.74)	1.00 (0.58)	0.50 (0.29)	0.75 (0.48)	0.25 (0.25)	0.25 (0.25)	1.00 (0.71)	0.25 (0.25)	0.00 (0.00)	0.33 (0.33)	0.33 (0.33)	2.00 (2.00)
**Chilopoda**	0.00 (0.00)	0.00 (0.00)	0.00 (0.00)	0.50 (0.29)	0.00 (0.00)	0.00 (0.00)	0.00 (0.00)	0.00 (0.00)	0.00 (0.00)	0.00 (0.00)	0.00 (0.00)	0.00 (0.00)
**Coleoptera**	15.75 (10.15)	6.75 (3.40)	7.00 (2.68)	21.25 (3.47)	9.00 (1.00)	15.50 (9.03)	34.50 (23.01)	15.25 (6.86)	11.25 (4.39)	33.33 (6.69)	9.33 (2.85)	31.00 (11.27)
**Collembola**	15.25 (10.90)	15.25 (8.84)	6.25 (3.54)	1.50 (0.50)	25.00 (15.85)	6.75 (2.29)	4.00 (2.12)	8.50 (3.59)	14.75 (4.59)	8.33 (4.48)	3.33 (1.86)	46.00 (41.02)
**Dermaptera**	1.75 (1.03)	1.75 (1.44)	1.75 (0.63)	1.50 (0.65)	0.00 (0.00)	0.00 (0.00)	0.50 (0.50)	0.25 (0.25)	0.00 (0.00)	2.33 (1.86)	0.00 (0.00)	5.67 (3.28)
**Diptera**	20.00 (10.81)	21.50 (13.13)	11.25 (4.53)	11.25 (3.30)	23.00 (8.03)	16.00 (5.20)	22.50 (13.67)	16.25 (4.11)	9.00 (4.06)	23.33 (5.55)	23.00 (7.77)	78.00 (59.03)
**Formicidae**	44.25 (27.55)	21.75 (10.49)	7.75 (2.75)	68.50 (43.65)	34.25 (18.99)	4.75 (0.48)	47.50 (25.98)	55.00 (45.12)	22.50 (9.51)	110.00 (28.04)	14.33 (9.02)	39.67 (16.50)
**Hemiptera**	16.25 (7.69)	14.00 (6.32)	7.50 (3.23)	8.75 (0.75)	20.00 (1.29)	11.75 (2.66)	5.25 (3.42)	9.25 (4.64)	13.75 (7.47)	10.00 (1.73)	10.67 (5.17)	13.67 (4.98)
**Hymenoptera**	18.75 (10.11)	15.00 (7.86)	17.50 (11.95)	7.50 (0.87)	17.25 (4.99)	8.25 (2.56)	55.00 (47.71)	13.00 (6.22)	15.00 (5.40)	24.33 (7.51)	22.33 (12.99)	45.33 (14.33)
**Isopoda**	0.00 (0.00)	0.25 (0.25)	0.00 (0.00)	0.00 (0.00)	0.00 (0.00)	0.00 (0.00)	0.00 (0.00)	0.00 (0.00)	0.00 (0.00)	0.00 (0.00)	0.00 (0.00)	0.00 (0.00)
**Larvae**	15.75 (6.88)	12.75 (7.89)	12.25 (2.66)	6.00 (3.19)	2.75 (1.55)	1.25 (0.25)	53.50 (47.27)	16.75 (5.88)	3.00 (1.35)	14.67 (1.33)	14.33 (7.54)	28.67 (8.82)
**Lepidoptera**	4.25 (2.39)	1.00 (0.41)	2.00 (0.71)	1.00 (0.00)	0.00 (0.00)	0.00 (0.00)	1.00 (0.58)	0.50 (0.29)	0.25 (0.25)	2.67 (0.67)	2.33 (1.86)	7.33 (1.45)
**Odonata**	0.00 (0.00)	0.00 (0.00)	0.00 (0.00)	0.00 (0.00)	0.00 (0.00)	0.25 (0.25)	0.00 (0.00)	0.00 (0.00)	0.00 (0.00)	0.00 (0.00)	0.00 (0.00)	0.00 (0.00)
**Orthoptera**	0.25 (0.25)	0.50 (0.50)	0.00 (0.00)	0.00 (0.00)	0.50 (0.29)	0.00 (0.00)	0.00 (0.00)	0.00 (0.00)	0.00 (0.00)	0.00 (0.00)	0.00 (0.00)	0.00 (0.00)
**Pseudoscorpiones**	0.00 (0.00)	0.00 (0.00)	0.00 (0.00)	0.00 (0.00)	0.00 (0.00)	0.00 (0.00)	0.00 (0.00)	0.00 (0.00)	0.00 (0.00)	0.00 (0.00)	0.33 (0.33)	0.00 (0.00)
**Psocoptera**	22.00 (16.29)	15.25 (11.37)	7.50 (4.99)	20.50 (10.24)	40.50 (25.85)	30.75 (12.51)	13.00 (4.18)	7.25 (3.54)	5.50 (2.53)	31.00 (2.89)	4.00 (3.51)	25.67 (7.86)
**Thysanoptera**	26.50 (15.71)	8.00 (4.04)	13.00 (6.65)	12.25 (3.35)	28.00 (13.40)	20.75 (14.06)	26.00 (22.06)	27.25 (13.73)	12.75 (4.52)	22.00 (8.33)	7.00 (1.73)	25.00 (12.66)
**Thysanura**	0.00 (0.00)	0.00 (0.00)	0.25 (0.25)	0.00 (0.00)	0.00 (0.00)	0.00 (0.00)	0.00 (0.00)	0.00 (0.00)	0.00 (0.00)	0.00 (0.00)	0.00 (0.00)	0.00 (0.00)
**Unknown**	0.75 (0.75)	0.00 (0.00)	0.25 (0.25)	0.00 (0.00)	0.00 (0.00)	0.00 (0.00)	0.00 (0.00)	0.00 (0.00)	0.00 (0.00)	0.00 (0.00)	0.00 (0.00)	0.00 (0.00)
**All**	**241.25 (138.63)**	**161.25 (84.50)**	**110.25 (47.70)**	**174.75 (40.34)**	**241.00 (57.95)**	**170.00 (39.30)**	**291.50 (197.75)**	**227.00 (62.36)**	**165.25 (48.66)**	**333.00 (72.34)**	**153.00 (54.08)**	**461.00 (213.52)**

Table S11. Arthropods (mean abundance per transect, with standard errors in brackets) that were collected in the ***understory microhabitat***. All arthropod groups were used in the total abundance analysis. All but arthropods that we could not identify to order or other group were also used in the order-level community composition analysis.

Understory arthropods: Abundance per transect (Standard error)

	Age M			Age 1			Age 3			Age 8		
**Order**	**Buffer**	**Edge**	**Core**	**Buffer**	**Edge**	**Core**	**Buffer**	**Edge**	**Core**	**Buffer**	**Edge**	**Core**
**Acari**	0.00 (0.00)	0.25 (0.25)	0.25 (0.25)	0.00 (0.00)	0.00 (0.00)	0.00 (0.00)	0.25 (0.25)	0.00 (0.00)	0.25 (0.25)	0.33 (0.33)	0.00 (0.00)	0.33 (0.33)
**Araneae**	14.50 (0.96)	12.25 (3.68)	19.75 (3.52)	10.75 (2.43)	17.00 (2.55)	17.50 (5.52)	7.50 (2.22)	9.25 (1.31)	8.75 (2.46)	11.67 (2.33)	10.67 (3.71)	10.00 (1.53)
**Blattodea**	0.75 (0.48)	1.00 (0.41)	0.25 (0.25)	2.75 (0.75)	2.75 (0.63)	3.75 (1.03)	1.00 (1.00)	0.75 (0.48)	1.50 (0.50)	0.67 (0.33)	0.00 (0.00)	0.67 (0.67)
**Coleoptera**	10.50 (2.90)	17.50 (3.01)	14.50 (1.66)	77.25 (22.68)	33.75 (6.90)	37.75 (12.95)	24.25 (3.50)	20.75 (2.69)	9.00 (2.45)	62.33 (47.90)	33.67 (25.67)	38.33 (26.39)
**Collembola**	0.00 (0.00)	0.00 (0.00)	2.25 (1.93)	0.25 (0.25)	0.25 (0.25)	2.00 (1.41)	0.25 (0.25)	0.25 (0.25)	0.50 (0.29)	0.00 (0.00)	0.00 (0.00)	0.00 (0.00)
**Dermaptera**	1.00 (0.71)	1.50 (0.29)	0.50 (0.50)	0.75 (0.25)	0.00 (0.00)	0.00 (0.00)	0.00 (0.00)	0.75 (0.75)	0.00 (0.00)	0.00 (0.00)	0.33 (0.33)	0.33 (0.33)
**Diptera**	190.00 (32.31)	155.25 (27.61)	180.50 (14.18)	211.00 (27.08)	210.25 (8.43)	209.75 (22.35)	330.75 (81.48)	150.75 (21.04)	136.75 (33.84)	260.00 (72.20)	122.00 (34.96)	206.33 (79.52)
**Formicidae**	8.75 (2.10)	6.50 (2.53)	8.25 (2.78)	7.75 (1.38)	27.00 (21.08)	15.00 (7.43)	4.25 (1.60)	3.75 (1.93)	2.50 (0.96)	3.00 (0.58)	2.67 (1.45)	4.33 (0.88)
**Hemiptera**	7.00 (1.78)	11.00 (2.48)	7.75 (4.77)	11.00 (2.68)	75.75 (16.49)	70.75 (12.76)	16.00 (1.96)	35.25 (4.71)	25.00 (7.79)	19.67 (13.86)	5.67 (1.86)	6.00 (2.65)
**Hymenoptera**	14.25 (1.44)	18.00 (2.74)	19.50 (3.66)	23.25 (4.87)	25.50 (2.25)	27.25 (8.23)	18.25 (0.75)	16.00 (1.15)	13.50 (5.07)	18.33 (4.67)	12.00 (2.08)	10.33 (4.33)
**Isoptera**	0.00 (0.00)	0.50 (0.29)	0.25 (0.25)	1.25 (1.25)	0.25 (0.25)	0.50 (0.50)	0.00 (0.00)	0.00 (0.00)	0.00 (0.00)	0.33 (0.33)	0.00 (0.00)	0.00 (0.00)
**Lepidoptera**	10.25 (1.25)	8.75 (1.65)	8.00 (1.68)	13.25 (2.75)	14.50 (4.03)	11.75 (3.82)	9.75 (1.80)	8.50 (1.32)	15.75 (3.61)	7.33 (1.67)	6.00 (2.00)	4.00 (0.58)
**Mantodea**	0.25 (0.25)	0.00 (0.00)	0.00 (0.00)	0.25 (0.25)	0.00 (0.00)	0.00 (0.00)	0.25 (0.25)	0.00 (0.00)	0.00 (0.00)	0.33 (0.33)	0.33 (0.33)	0.33 (0.33)
**Odonata**	0.00 (0.00)	0.00 (0.00)	0.00 (0.00)	0.00 (0.00)	0.00 (0.00)	0.25 (0.25)	0.00 (0.00)	0.00 (0.00)	0.25 (0.25)	0.33 (0.33)	0.00 (0.00)	0.00 (0.00)
**Orthoptera**	0.50 (0.29)	0.75 (0.75)	1.00 (1.00)	0.25 (0.25)	1.50 (1.19)	0.50 (0.29)	0.50 (0.29)	0.00 (0.00)	0.00 (0.00)	1.33 (0.88)	0.67 (0.67)	0.00 (0.00)
**Pseudoscorpiones**	0.00 (0.00)	0.00 (0.00)	0.00 (0.00)	0.00 (0.00)	0.00 (0.00)	0.00 (0.00)	0.00 (0.00)	0.00 (0.00)	0.00 (0.00)	0.33 (0.33)	0.00 (0.00)	0.00 (0.00)
**Psocoptera**	2.00 (0.82)	2.25 (0.25)	4.25 (1.44)	4.00 (1.41)	12.25 (1.31)	11.50 (1.55)	3.00 (0.71)	6.50 (2.40)	3.75 (1.60)	1.33 (0.33)	2.00 (0.58)	0.33 (0.33)
**Thysanoptera**	0.75 (0.48)	2.75 (0.75)	3.00 (1.47)	3.50 (1.55)	7.50 (2.63)	7.00 (3.67)	4.00 (0.82)	6.25 (1.55)	5.25 (0.85)	4.00 (3.00)	0.67 (0.33)	1.00 (0.58)
**Unknown**	13.75 (2.39)	17.50 (1.76)	14.00 (1.78)	14.50 (4.21)	15.50 (5.91)	23.50 (3.97)	15.75 (2.78)	12.25 (2.69)	17.50 (6.74)	9.67 (2.03)	9.33 (2.96)	12.33 (1.20)
**All**	**248.75 (28.48)**	**255.75 (35.27)**	**284.00 (23.78)**	**381.75 (40.55)**	**443.75 (35.48)**	**438.75 (36.02)**	**435.75 (83.47)**	**271.00 (30.05)**	**240.25 (40.69)**	**358.33 (124.80)**	**171.67 (23.69)**	**294.67 (107.02)**

Table S12. Arthropods (mean abundance per transect, with standard errors in brackets) that were collected in the ***ground microhabitat.*** All arthropod groups were used in the total abundance analysis. All but arthropods that we could not identify to order or other group were also used in the order-level community composition analysis.

Ground arthropods: Abundance per transect (Standard error)

	Age M			Age 1			Age 3			Age 8		
**Order**	**Buffer**	**Edge**	**Core**	**Buffer**	**Edge**	**Core**	**Buffer**	**Edge**	**Core**	**Buffer**	**Edge**	**Core**
**Acari**	101.50 (76.59)	11.25 (4.50)	18.75 (8.59)	6.25 (2.63)	1.00 (0.58)	2.50 (1.85)	13.00 (4.42)	13.25 (2.56)	17.50 (9.95)	16.33 (6.98)	5.00 (2.08)	7.00 (3.79)
**Araneae**	16.00 (1.96)	17.50 (2.33)	23.75 (6.17)	11.50 (1.85)	17.75 (5.45)	23.75 (15.80)	13.00 (3.19)	14.00 (4.60)	36.50 (19.46)	7.67 (3.18)	8.00 (2.65)	5.33 (1.67)
**Blattodea**	6.25 (4.92)	3.50 (1.85)	1.25 (0.25)	4.25 (0.95)	4.25 (1.97)	5.25 (2.56)	2.75 (1.80)	2.75 (1.25)	1.75 (0.25)	7.67 (4.18)	0.33 (0.33)	0.33 (0.33)
**Chilopoda**	0.25 (0.25)	0.50 (0.29)	0.75 (0.48)	0.50 (0.50)	1.00 (0.41)	0.25 (0.25)	0.75 (0.48)	0.75 (0.48)	2.00 (1.15)	0.00 (0.00)	0.33 (0.33)	0.00 (0.00)
**Coleoptera**	84.75 (20.73)	118.50 (54.83)	84.00 (16.48)	41.00 (16.76)	25.00 (5.15)	25.25 (4.68)	44.25 (8.48)	35.00 (9.76)	15.50 (4.09)	194.33 (11.68)	66.00 (23.44)	86.00 (13.65)
**Collembola**	61.00 (31.67)	640.75 (625.12)	670.25 (641.59)	15.00 (5.72)	27.25 (8.56)	33.00 (15.59)	72.50 (30.93)	113.50 (32.97)	200.50 (105.41)	45.33 (28.09)	50.33 (31.99)	18.33 (14.52)
**Dermaptera**	14.00 (2.04)	5.50 (1.55)	10.50 (5.87)	8.25 (4.50)	1.25 (0.48)	1.50 (0.96)	17.50 (6.66)	1.25 (1.25)	1.50 (0.96)	10.67 (3.93)	1.67 (0.88)	3.00 (2.52)
**Diplopoda**	0.75 (0.48)	1.25 (0.63)	1.00 (0.41)	0.25 (0.25)	6.50 (3.33)	1.25 (0.75)	0.75 (0.48)	0.25 (0.25)	2.00 (1.41)	0.33 (0.33)	1.33 (1.33)	0.33 (0.33)
**Diptera**	6.00 (1.68)	9.50 (1.50)	5.00 (1.58)	8.00 (2.45)	7.25 (4.33)	3.50 (0.96)	25.00 (7.71)	16.75 (3.50)	8.25 (2.02)	11.00 (2.31)	15.00 (4.93)	8.00 (6.11)
**Formicidae**	91.00 (12.93)	82.75 (15.36)	120.25 (25.80)	103.50 (44.15)	290.50 (95.58)	297.50 (120.21)	125.25 (23.70)	104.00 (39.28)	110.25 (27.81)	78.00 (19.04)	36.67 (11.46)	35.67 (13.45)
**Hemiptera**	11.25 (3.25)	4.00 (1.08)	11.00 (3.81)	3.25 (1.97)	17.50 (7.49)	15.00 (7.52)	11.25 (2.78)	18.50 (9.30)	16.25 (6.14)	6.67 (1.45)	4.67 (2.91)	4.33 (1.45)
**Hymenoptera**	1.00 (0.71)	1.75 (0.48)	2.50 (0.65)	2.75 (2.10)	1.75 (0.75)	2.00 (0.41)	3.25 (0.95)	2.75 (0.85)	3.25 (0.75)	2.67 (1.20)	1.33 (0.88)	1.67 (1.20)
**Isopod**	6.00 (3.08)	0.75 (0.25)	4.00 (1.78)	7.25 (2.87)	3.00 (2.04)	1.75 (1.44)	8.00 (3.24)	2.50 (2.18)	0.00 (0.00)	8.33 (3.33)	0.67 (0.33)	0.00 (0.00)
**Isoptera**	0.00 (0.00)	0.00 (0.00)	0.25 (0.25)	0.00 (0.00)	0.00 (0.00)	0.25 (0.25)	0.00 (0.00)	0.00 (0.00)	0.00 (0.00)	0.00 (0.00)	0.00 (0.00)	0.00 (0.00)
**Larvae**	4.50 (1.55)	5.00 (1.08)	3.00 (2.04)	1.00 (1.00)	1.00 (0.71)	2.25 (2.25)	5.00 (2.74)	7.25 (3.57)	3.50 (1.26)	3.67 (2.67)	10.33 (7.97)	2.00 (1.53)
**Lepidoptera**	0.25 (0.25)	0.25 (0.25)	0.00 (0.00)	0.00 (0.00)	0.00 (0.00)	0.00 (0.00)	0.00 (0.00)	0.00 (0.00)	0.00 (0.00)	1.00 (0.58)	0.00 (0.00)	0.00 (0.00)
**Neuroptera**	0.00 (0.00)	0.00 (0.00)	0.25 (0.25)	0.00 (0.00)	0.00 (0.00)	0.00 (0.00)	0.00 (0.00)	0.00 (0.00)	0.00 (0.00)	0.00 (0.00)	0.00 (0.00)	0.00 (0.00)
**Opiliones**	0.00 (0.00)	0.00 (0.00)	0.00 (0.00)	0.00 (0.00)	0.00 (0.00)	0.00 (0.00)	0.00 (0.00)	0.00 (0.00)	0.50 (0.50)	0.33 (0.33)	0.00 (0.00)	0.00 (0.00)
**Orthoptera**	34.00 (8.19)	18.75 (5.38)	41.50 (16.47)	12.75 (2.93)	16.75 (6.59)	10.75 (4.61)	9.50 (1.04)	10.25 (1.44)	3.75 (1.55)	13.33 (1.76)	14.33 (4.84)	12.33 (5.21)
**Pseudoscorpiones**	0.75 (0.25)	0.00 (0.00)	0.25 (0.25)	0.50 (0.50)	0.00 (0.00)	0.00 (0.00)	0.00 (0.00)	0.00 (0.00)	0.00 (0.00)	2.00 (0.58)	0.00 (0.00)	0.00 (0.00)
**Psocoptera**	2.00 (1.22)	0.25 (0.25)	1.25 (1.25)	0.25 (0.25)	0.75 (0.48)	0.50 (0.29)	0.25 (0.25)	1.75 (0.85)	1.00 (1.00)	0.00 (0.00)	1.67 (1.20)	0.00 (0.00)
**Scorpiones**	0.00 (0.00)	0.00 (0.00)	0.00 (0.00)	0.00 (0.00)	0.00 (0.00)	0.00 (0.00)	0.00 (0.00)	0.00 (0.00)	0.00 (0.00)	0.33 (0.33)	0.00 (0.00)	0.00 (0.00)
**Thysanoptera**	0.25 (0.25)	0.00 (0.00)	1.00 (0.41)	0.00 (0.00)	0.75 (0.75)	0.00 (0.00)	1.50 (0.96)	1.75 (1.18)	2.25 (1.11)	0.00 (0.00)	0.00 (0.00)	0.67 (0.67)
**Unknown**	0.00 (0.00)	0.00 (0.00)	0.00 (0.00)	0.00 (0.00)	0.00 (0.00)	0.00 (0.00)	0.50 (0.50)	0.00 (0.00)	0.00 (0.00)	0.00 (0.00)	0.00 (0.00)	0.00 (0.00)
**All**	**441.50 (132.04)**	**921.75 (613.82)**	**1000.50 (662.53)**	**226.25 (77.80)**	**406.75 (121.08)**	**408.00 (135.72)**	**354.25 (24.71)**	**346.25 (89.86)**	**426.25 (147.01)**	**409.67 (63.25)**	**210.33 (91.51)**	**185.00 (31.01)**

Table S13. Effects of *Cohort* and *Distance* on ***total arthropod abundance*** in the canopy, understory, and ground microhabitats. For each response, the model with the lowest exact leave-one-out information criterion (LOOIC), and its standard error, is listed first. The difference in expected log pointwise densities (ELPD) and corresponding standard errors is also provided. The optimal model is indicated in bold. The optimal model is that with the lowest LOOIC, unless the standard errors of the difference in ELPD of other models exceeded the difference in ELPD between these models and the model with the lowest LOOIC. In this case, we chose the more parsimonious model.

		Model	LOOIC	SE LOOIC	ELPD Difference	SE Difference
Abundance	*Canopy*	~ Cohort + (1 | Site)	557.2	15.6	0.0	0.0
		**~ 1 + (1 | Site)**	**557.2**	**15.4**	**0.0**	**0.7**
		~ Distance + (1 | Site)	562.9	15.3	-2.9	1.2
		~ Cohort + Distance + (1 | Site)	563.0	15.6	-2.9	0.7
		~ Cohort*Distance + (1 | Site)	566.1	15.3	-4.5	2.6
	Understory	**~ Cohort*Distance + (1 | Site)**	**1310.5**	**19.8**	**0.0**	**0.0**
		~ Distance + (1 | Site)	1327.8	21.1	-8.6	4.9
		~ Cohort + Distance + (1 | Site)	1328.0	21.5	-8.7	5.0
		~ 1 + (1 | Site)	1336.2	23.5	-12.5	6.2
		~ Cohort + (1 | Site)	1335.6	23.0	-12.9	6.4
	Ground	**~ 1 + (1 | Site)**	**1548.5**	**28.0**	**0.0**	**0.0**
		~ Cohort + (1 | Site)	1548.9	28.0	-0.2	0.5
		~ Cohort*Distance + (1 | Site)	1550.4	29.1	-0.9	2.7
		~ Distance + (1 | Site)	1553.2	28.1	-2.3	0.2
		~ Cohort + Distance + (1 | Site)	1553.8	28.3	-2.6	0.5

Table S14. Model summary tables for GLMM analyses used to explain differences in ***canopy arthropod abundance*** across the chronosequence. We present model estimates and associated standard errors and 95% credible intervals for each fixed parameter and the negative binomial shape parameter. In all models, we specified *Age M-Buffer* as the intercept (Cohort: *Age M*, *Age 1*, *Age 3*, *Age 8;* Distance: *Buffer*, *Edge*, *Core*).

Canopy abundance ~ Cohort*Distance + (1 | Site)

Parameter	*Estimate*	*SE*	*95% CI*
Intercept	5.06	0.38	4.32 - 5.83
Age1	0.13	0.53	-0.91 - 1.16
Age3	0.19	0.53	-0.87 - 1.22
Age8	0.68	0.55	-0.42 - 1.76
Edge	-0.16	0.33	-0.79 - 0.49
Core	-0.34	0.33	-0.98 - 0.32
Age1:Edge	0.41	0.47	-0.51 - 1.32
Age3:Edge	0.24	0.47	-0.69 - 1.16
Age8:Edge	-0.62	0.51	-1.60 - 0.40
Age1:Core	0.25	0.47	-0.68 - 1.16
Age3:Core	0.22	0.49	-0.75 - 1.18
Age8:Core	0.50	0.50	-0.48 - 1.48
shape	3.13	0.87	1.67 - 5.08

Canopy abundance ~ Cohort + Distance + (1 | Site)

Parameter	*Estimate*	*SE*	*95% CI*
Intercept	5.01	0.38	4.27 - 5.78
Age1	0.28	0.49	-0.71 - 1.24
Age3	0.27	0.50	-0.73 - 1.24
Age8	0.67	0.53	-0.40 - 1.69
Edge	-0.09	0.23	-0.54 - 0.36
Core	-0.12	0.23	-0.57 - 0.33
shape	2.84	0.74	1.59 - 4.47

Canopy abundance ~ Cohort + (1 | Site)

Parameter	*Estimate*	*SE*	*95% CI*
Intercept	4.93	0.36	4.24 - 5.66
Age1	0.28	0.50	-0.72 - 1.24
Age3	0.27	0.50	-0.74 - 1.23
Age8	0.67	0.53	-0.42 - 1.69
shape	3.01	0.76	1.72 - 4.67

Canopy abundance ~ Distance + (1 | Site)

Parameter	*Estimate*	*SE*	*95% CI*
Intercept	5.30	0.26	4.78 - 5.82
Edge	-0.10	0.23	-0.56 - 0.36
Core	-0.11	0.23	-0.56 - 0.35
shape	2.81	0.74	1.55 - 4.43

Canopy abundance ~ 1 + (1 | Site)

Parameter	*Estimate*	*SE*	*95% CI*
Intercept	5.22	0.23	4.76 - 5.66
shape	2.97	0.76	1.68 - 4.64

Table S15. Model summary tables for GLMM analyses used to explain differences in ***understory arthropod abundance*** across the chronosequence. We present model estimates and associated standard errors and 95% credible intervals for each fixed parameter and the negative binomial shape parameter. In all models, we specified *Age M-Buffer* as the intercept (Cohort: *Age M*, *Age 1*, *Age 3*, *Age 8;* Distance: *Buffer*, *Edge*, *Core*).

Understory abundance ~ Cohort*Distance + (1 | Site)

Parameter	*Estimate*	*SE*	*95% CI*
Intercept	4.56	0.17	4.23 - 4.90
Age1	0.29	0.24	-0.19 - 0.75
Age3	0.37	0.24	-0.11 - 0.83
Age8	0.22	0.26	-0.29 - 0.73
Edge	-0.11	0.13	-0.36 - 0.14
Core	0.00	0.13	-0.25 - 0.25
Age1:Edge	0.26	0.18	-0.09 - 0.60
Age3:Edge	-0.31	0.18	-0.66 - 0.04
Age8:Edge	-0.51	0.20	-0.90 - -0.11
Age1:Core	0.13	0.18	-0.21 - 0.48
Age3:Core	-0.58	0.18	-0.92 - -0.23
Age8:Core	-0.31	0.19	-0.69 - 0.07
shape	11.63	1.74	8.53 - 15.34

Understory abundance ~ Cohort + Distance + (1 | Site)

Parameter	*Estimate*	*SE*	*95% CI*
Intercept	4.66	0.16	4.33 - 4.99
Age1	0.43	0.22	-0.03 - 0.87
Age3	0.10	0.22	-0.36 - 0.53
Age8	-0.02	0.24	-0.50 - 0.45
Edge	-0.22	0.07	-0.37 - -0.08
Core	-0.18	0.07	-0.33 - -0.04
shape	9.51	1.35	7.10 - 12.36

Understory abundance ~ Cohort + (1 | Site)

Parameter	*Estimate*	*SE*	*95% CI*
Intercept	4.52	0.16	4.20 - 4.83
Age1	0.42	0.23	-0.04 - 0.87
Age3	0.12	0.23	-0.34 - 0.56
Age8	0.00	0.24	-0.49 - 0.48
shape	8.84	1.24	6.61 - 11.46

Understory abundance ~ Distance + (1 | Site)

Parameter	*Estimate*	*SE*	*95% CI*
Intercept	4.79	0.10	4.59 - 4.99
Edge	-0.22	0.07	-0.36 - -0.07
Core	-0.18	0.07	-0.33 - -0.04
shape	9.51	1.35	7.08 - 12.37

Understory abundance ~ 1 + (1 | Site)

Parameter	*Estimate*	*SE*	*95% CI*
Intercept	4.66	0.09	4.47 - 4.84
shape	8.84	1.24	6.61 - 11.45

Table S16. Model summary tables for GLMM analyses used to explain differences in ***ground arthropod abundance*** across the chronosequence. We present model estimates and associated standard errors and 95% credible intervals for each fixed parameter and the negative binomial shape parameter. In all models, we specified *Age M-Buffer* as the intercept (Cohort: *Age M*, *Age 1*, *Age 3*, *Age 8;* Distance: *Buffer*, *Edge*, *Core*).

Ground abundance ~ Cohort*Distance + (1 | Site)

Parameter	*Estimate*	*SE*	*95% CI*
Intercept	5.03	0.32	4.38 - 5.65
Age1	-0.66	0.45	-1.54 - 0.25
Age3	-0.19	0.45	-1.07 - 0.71
Age8	-0.14	0.48	-1.07 - 0.81
Edge	0.10	0.25	-0.38 - 0.59
Core	0.19	0.25	-0.30 - 0.67
Age1:Edge	0.47	0.35	-0.23 - 1.16
Age3:Edge	-0.22	0.35	-0.91 - 0.47
Age8:Edge	-0.65	0.39	-1.40 - 0.12
Age1:Core	0.31	0.36	-0.40 - 1.01
Age3:Core	-0.15	0.35	-0.85 - 0.54
Age8:Core	-0.87	0.37	-1.61 - -0.13
shape	2.20	0.28	1.68 - 2.79

Ground abundance ~ Cohort + Distance + (1 | Site)

Parameter	*Estimate*	*SE*	*95% CI*
Intercept	5.09	0.32	4.45 - 5.70
Age1	-0.38	0.43	-1.21 - 0.48
Age3	-0.29	0.42	-1.10 - 0.57
Age8	-0.50	0.46	-1.38 - 0.43
Edge	0.01	0.16	-0.29 - 0.32
Core	0.03	0.16	-0.28 - 0.33
shape	2.06	0.26	1.59 - 2.60

Ground abundance ~ Cohort + (1 | Site)

Parameter	*Estimate*	*SE*	*95% CI*
Intercept	5.09	0.30	4.47 - 5.68
Age1	-0.38	0.43	-1.20 - 0.50
Age3	-0.29	0.43	-1.10 - 0.57
Age8	-0.51	0.46	-1.38 - 0.42
shape	2.09	0.26	1.62 - 2.63

Ground abundance ~ Distance + (1 | Site)

Parameter	*Estimate*	*SE*	*95% CI*
Intercept	4.80	0.20	4.41 - 5.20
Edge	0.02	0.15	-0.28 - 0.32
Core	0.03	0.16	-0.27 - 0.34
shape	2.06	0.26	1.59 - 2.60

Ground abundance ~ 1 + (1 | Site)

Parameter	*Estimate*	*SE*	*95% CI*
Intercept	4.82	0.18	4.46 - 5.17
shape	2.09	0.26	1.62 - 2.64

Table S17. Pairwise comparisons of estimated marginal means between factor levels in the optimal ***understory arthropod abundance*** model: *Abundance ~ Cohort*Distance + (1 | Site)*. We concluded that factor levels were meaningfully different if the 95% highest posterior density (HPD) interval of the median point estimate calculated from our comparisons did not overlap with zero (indicated in bold). Results are given on the log scale and with a HPD interval probability of 0.95.

Comparison			Lower HPD	Upper HPD	Estimate
Age M-Buffer	---	Age 1-Buffer	-0.542	0.936	0.188
Age M-Buffer	---	Age 3-Buffer	-0.161	1.329	0.596
Age M-Buffer	---	Age 8-Buffer	-0.578	1.009	0.203
Age M-Buffer	---	Age M-Edge	-0.443	0.286	-0.080
Age M-Buffer	**---**	Age 1-Edge	**0.386**	**1.947**	**1.155**
Age M-Buffer	---	Age 3-Edge	-0.456	1.068	0.327
Age M-Buffer	---	Age 8-Edge	-0.805	0.831	0.022
Age M-Buffer	---	Age M-Core	-0.572	0.161	-0.204
Age M-Buffer	**---**	Age 1-Core	**0.140**	**1.694**	**0.915**
Age M-Buffer	---	Age 3-Core	-0.860	0.663	-0.072
Age M-Buffer	---	Age 8-Core	-0.798	0.833	0.017
Age 1-Buffer	---	Age 3-Buffer	-0.426	1.198	0.406
Age 1-Buffer	---	Age 8-Buffer	-0.860	0.871	0.015
Age 1-Buffer	---	Age M-Edge	-1.042	0.496	-0.269
Age 1-Buffer	**---**	Age 1-Edge	**0.515**	**1.401**	**0.968**
Age 1-Buffer	---	Age 3-Edge	-0.686	0.952	0.139
Age 1-Buffer	---	Age 8-Edge	-1.025	0.724	-0.164
Age 1-Buffer	---	Age M-Core	-1.147	0.387	-0.391
Age 1-Buffer	**---**	Age 1-Core	**0.296**	**1.148**	**0.729**
Age 1-Buffer	---	Age 3-Core	-1.101	0.536	-0.260
Age 1-Buffer	---	Age 8-Core	-1.053	0.701	-0.173
Age 3-Buffer	---	Age 8-Buffer	-1.259	0.468	-0.393
Age 3-Buffer	---	Age M-Edge	-1.432	0.115	-0.675
Age 3-Buffer	---	Age 1-Edge	-0.262	1.422	0.559
Age 3-Buffer	---	Age 3-Edge	-0.697	0.153	-0.270
Age 3-Buffer	---	Age 8-Edge	-1.447	0.316	-0.573
Age 3-Buffer	**---**	Age M-Core	**-1.564**	**-0.017**	**-0.800**
Age 3-Buffer	---	Age 1-Core	-0.517	1.157	0.319
Age 3-Buffer	**---**	Age 3-Core	**-1.087**	**-0.243**	**-0.670**
Age 3-Buffer	---	Age 8-Core	-1.451	0.308	-0.579
Age 8-Buffer	---	Age M-Edge	-1.095	0.543	-0.285
Age 8-Buffer	**---**	Age 1-Edge	**0.076**	**1.855**	**0.951**
Age 8-Buffer	---	Age 3-Edge	-0.769	0.986	0.123
Age 8-Buffer	---	Age 8-Edge	-0.642	0.277	-0.181
Age 8-Buffer	---	Age M-Core	-1.218	0.416	-0.408
Age 8-Buffer	---	Age 1-Core	-0.172	1.600	0.711
Age 8-Buffer	---	Age 3-Core	-1.167	0.575	-0.277
Age 8-Buffer	---	Age 8-Core	-0.637	0.286	-0.187
**Age M-Edge**	**---**	**Age 1-Edge**	**0.434**	**2.029**	**1.235**
Age M-Edge	---	Age 3-Edge	-0.386	1.170	0.408
Age M-Edge	---	Age 8-Edge	-0.755	0.914	0.105
Age M-Edge	---	Age M-Core	-0.508	0.264	-0.123
**Age M-Edge**	**---**	**Age 1-Core**	**0.202**	**1.788**	**0.996**
Age M-Edge	---	Age 3-Core	-0.797	0.759	0.009
Age M-Edge	---	Age 8-Core	-0.751	0.916	0.097
**Age 1-Edge**	**---**	**Age 3-Edge**	**-1.695**	**-0.001**	**-0.829**
**Age 1-Edge**	**---**	**Age 8-Edge**	**-2.040**	**-0.239**	**-1.131**
**Age 1-Edge**	**---**	**Age M-Core**	**-2.161**	**-0.564**	**-1.358**
Age 1-Edge	---	Age 1-Core	-0.697	0.245	-0.241
**Age 1-Edge**	**---**	**Age 3-Core**	**-2.100**	**-0.408**	**-1.228**
**Age 1-Edge**	**---**	**Age 8-Core**	**-2.053**	**-0.248**	**-1.137**
Age 3-Edge	---	Age 8-Edge	-1.194	0.589	-0.305
Age 3-Edge	---	Age M-Core	-1.315	0.252	-0.530
Age 3-Edge	---	Age 1-Core	-0.227	1.456	0.587
Age 3-Edge	---	Age 3-Core	-0.827	0.015	-0.399
Age 3-Edge	---	Age 8-Core	-1.199	0.575	-0.310
Age 8-Edge	---	Age M-Core	-1.037	0.634	-0.227
Age 8-Edge	---	Age 1-Core	-0.018	1.779	0.893
Age 8-Edge	---	Age 3-Core	-0.970	0.801	-0.096
Age 8-Edge	---	Age 8-Core	-0.487	0.456	-0.005
**Age M-Core**	**---**	**Age 1-Core**	**0.338**	**1.916**	**1.121**
Age M-Core	---	Age 3-Core	-0.653	0.900	0.131
Age M-Core	---	Age 8-Core	-0.610	1.048	0.220
**Age 1-Core**	**---**	**Age 3-Core**	**-1.839**	**-0.160**	**-0.988**
**Age 1-Core**	**---**	**Age 8-Core**	**-1.817**	**-0.010**	**-0.897**
Age 3-Core	---	Age 8-Core	-0.796	0.976	0.091

Table S18. Results of post-hoc tests assessing differences in the optimal ***understory arthropod order-level community composition*** model: *Composition ~ Cohort + (1 | Site)*. We indicate significant pairwise comparisons in bold font: *** = $p < 0.001$, ** = $p < 0.01$, * = $p < 0.05$. The significance of cohort to the model, and associated likelihood ratio test statistic, are also provided.

Factor	LRT statistic	p-value
f(Cohort)	**224.0**	**<0.001*** **

Comparison			LRT statistic	p-value
Age M	---	Age 1	109.64	**<0.001*** **
Age 1	---	Age 8	104.26	**<0.001*** **
Age 1	---	Age 3	75.56	**<0.001*** **
Age M	---	Age 3	61.32	**0.006** **
Age 3	---	Age 8	53.92	**0.013* **
Age M	---	Age 8	49.4	**0.013* **

Table S19. Results of post-hoc tests assessing differences in the optimal ***ground arthropod order-level community composition*** model: *Composition ~ Cohort + (1 | Site)*. We indicate significant pairwise comparisons in bold font: *** = $p < 0.001$, ** = $p < 0.01$, * = $p < 0.05$. The significance of cohort to the model, and associated likelihood ratio test statistic, are also provided.

Factor	LRT statistic	p-value
f(Cohort)	**228.1**	**0.008****

Comparison			LRT statistic	p-value
Age M	---	Age 3	86.78	**0.007****
Age 3	---	Age 8	81.73	**0.01****
Age M	---	Age 1	77.34	**0.013***
Age M	---	Age 8	66.78	**0.027***
Age 1	---	Age 8	65.13	**0.027***
Age 1	---	Age 3	63.54	**0.027***

Table S20. Effects of *Cohort* and *Distance* on ***spider abundance and species richness*** in the canopy, understory, and ground microhabitats. For each response, the model with the lowest exact leave-one-out information criterion (LOOIC), and its standard error, is listed first. The difference in expected log pointwise densities (ELPD) and corresponding standard errors is also provided. The optimal model is indicated in bold. The optimal model is that with the lowest LOOIC, unless the standard errors of the difference in ELPD of other models exceeded the difference in ELPD between these models and the model with the lowest LOOIC. In this case, we chose the more parsimonious model.

		Model	LOOIC	SE LOOIC	ELPD Difference	SE Difference
Abundance	*Canopy*	~ Distance + (1 | Site)	369.6	12.9	0.0	0.0
		~ Cohort + (1 | Site)	369.9	12.1	-0.2	2.3
		~ Cohort + Distance + (1 | Site)	370.1	13.4	-0.3	1.4
		**~ 1 + (1 | Site)**	**370.4**	**12.2**	**-0.4**	**2.5**
		~ Cohort*Distance + (1 | Site)	372.8	15.1	-1.6	3.0
	Understory	**~ Cohort*Distance + (1 | Site)**	**395.1**	**12.6**	**0.0**	**0.0**
		~ 1 + (1 | Site)	418.9	10.5	-11.9	6.4
		~ Cohort + (1 | Site)	418.9	12.4	-11.9	6.8
		~ Distance + (1 | Site)	421.6	10.2	-13.2	6.4
		~ Cohort + Distance + (1 | Site)	421.8	12.3	-13.4	6.7
	Ground	**~ 1 + (1 | Site)**	**720.3**	**25.3**	**0.0**	**0.0**
		~ Cohort + (1 | Site)	720.6	25.2	-0.2	0.9
		~ Distance + (1 | Site)	721.2	23.5	-0.5	2.0
		~ Cohort + Distance + (1 | Site)	722.0	23.6	-0.9	2.4
		~ Cohort*Distance + (1 | Site)	725.1	22.8	-2.4	3.7
Species Richness	*Canopy*	**~ 1 + (1 | Site)**	**217.0**	**12.7**	**0.0**	**0.0**
		~ Cohort + (1 | Site)	217.6	12.7	-0.3	0.9
		~ Cohort*Distance + (1 | Site)	218.6	11.5	-0.8	4.0
		~ Distance + (1 | Site)	220.9	13.3	-2.0	2.0
		~ Cohort + Distance + (1 | Site)	221.4	13.4	-2.2	2.4
	Understory	~ Cohort*Distance + (1 | Site)	218.3	6.0	0.0	0.0
		**~ Cohort + (1 | Site)**	**225.8**	**10.2**	**-3.8**	**4.4**
		~ 1 + (1 | Site)	228.6	7.8	-5.2	4.0
		~ Cohort + Distance + (1 | Site)	229.8	9.9	-5.8	4.2
		~ Distance + (1 | Site)	233.0	7.6	-7.4	3.8
	Ground	**~ 1 + (1 | Site)**	**445.3**	**19.0**	**0.0**	**0.0**
		~ Cohort + (1 | Site)	445.9	19.6	-0.3	1.1
		~ Cohort*Distance + (1 | Site)	447.0	19.5	-0.8	3.9
		~ Distance + (1 | Site)	447.9	18.7	-1.3	1.4
		~ Cohort + Distance + (1 | Site)	448.4	19.4	-1.6	1.9

Table S21. Model summary tables for GLMM analyses used to explain differences in ***canopy spider abundance*** across the chronosequence. We present model estimates and associated standard errors and 95% credible intervals for each fixed parameter and the negative binomial shape parameter. In all models, we specified *Age M-Buffer* as the intercept (Cohort: *Age M*, *Age 1*, *Age 3*, *Age 8;* Distance: *Buffer*, *Edge*, *Core*).

Canopy abundance ~ Cohort*Distance + (1 | Site)

Parameter	*Estimate*	*SE*	*95% CI*
Intercept	2.63	0.36	1.92 - 3.32
Age1	-0.20	0.50	-1.17 - 0.80
Age3	-0.20	0.49	-1.18 - 0.78
Age8	0.83	0.51	-0.17 - 1.84
Edge	0.10	0.35	-0.59 - 0.81
Core	-0.01	0.37	-0.71 - 0.73
Age1:Edge	0.63	0.51	-0.40 - 1.61
Age3:Edge	0.26	0.52	-0.77 - 1.27
Age8:Edge	-0.24	0.53	-1.27 - 0.82
Age1:Core	0.92	0.52	-0.14 - 1.92
Age3:Core	0.74	0.54	-0.34 - 1.78
Age8:Core	0.58	0.53	-0.46 - 1.61
shape	2.92	1.06	1.37 - 5.46

Canopy abundance ~ Cohort + Distance + (1 | Site)

Parameter	*Estimate*	*SE*	*95% CI*
Intercept	2.56	0.35	1.86 - 3.25
Age1	0.16	0.44	-0.69 - 1.03
Age3	-0.02	0.43	-0.87 - 0.84
Age8	0.83	0.46	-0.10 - 1.73
Edge	0.22	0.26	-0.31 - 0.73
Core	0.49	0.27	-0.04 - 1.01
shape	2.44	0.78	1.26 - 4.25

Canopy abundance ~ Cohort + (1 | Site)

Parameter	*Estimate*	*SE*	*95% CI*
Intercept	2.78	0.31	2.16 - 3.39
Age1	0.22	0.42	-0.60 - 1.06
Age3	0.03	0.42	-0.79 - 0.87
Age8	0.87	0.44	-0.02 - 1.74
shape	2.18	0.65	1.19 - 3.71

Canopy abundance ~ Distance + (1 | Site)

Parameter	*Estimate*	*SE*	*95% CI*
Intercept	2.76	0.26	2.24 - 3.28
Edge	0.20	0.27	-0.33 - 0.72
Core	0.51	0.26	-0.01 - 1.02
shape	2.43	0.79	1.22 - 4.27

Canopy abundance ~ 1 + (1 | Site)

Parameter	*Estimate*	*SE*	*95% CI*
Intercept	3.03	0.20	2.62 - 3.42
shape	2.17	0.67	1.14 - 3.72

Table S22. Model summary tables for GLMM analyses used to explain differences in ***understory spider abundance*** across the chronosequence. We present model estimates and associated standard errors and 95% credible intervals for each fixed parameter and the negative binomial shape parameter. In all models, we specified *Age M-Buffer* as the intercept (Cohort: *Age M*, *Age 1*, *Age 3*, *Age 8;* Distance: *Buffer*, *Edge*, *Core*).

Understory abundance ~ Cohort*Distance + (1 | Site)

Parameter	*Estimate*	*SE*	*95% CI*
Intercept	4.09	0.27	3.55 - 4.61
Age1	-0.19	0.37	-0.92 - 0.56
Age3	-0.59	0.38	-1.31 - 0.18
Age8	-0.20	0.40	-0.98 - 0.61
Edge	0.08	0.18	-0.29 - 0.44
Core	0.20	0.18	-0.16 - 0.57
Age1:Edge	-1.05	0.28	-1.60 - -0.48
Age3:Edge	0.19	0.27	-0.35 - 0.73
Age8:Edge	0.10	0.29	-0.47 - 0.67
Age1:Core	-0.93	0.28	-1.47 - -0.38
Age3:Core	0.47	0.27	-0.08 - 1.01
Age8:Core	-0.02	0.29	-0.59 - 0.56
shape	17.43	7.79	6.98 - 36.52

Understory abundance ~ Cohort + Distance + (1 | Site)

Parameter	*Estimate*	*SE*	*95% CI*
Intercept	4.22	0.26	3.70 - 4.73
Age1	-0.69	0.34	-1.36 - 0.00
Age3	-0.36	0.34	-1.00 - 0.34
Age8	-0.17	0.36	-0.86 - 0.57
Edge	-0.14	0.16	-0.46 - 0.19
Core	0.06	0.16	-0.27 - 0.38
shape	6.43	2.17	3.10 - 11.44

Understory abundance ~ Cohort + (1 | Site)

Parameter	*Estimate*	*SE*	*95% CI*
Intercept	4.20	0.23	3.71 - 4.65
Age1	-0.69	0.33	-1.34 - -0.01
Age3	-0.35	0.33	-0.99 - 0.33
Age8	-0.17	0.35	-0.85 - 0.55
shape	6.51	2.15	3.22 - 11.53

Understory abundance ~ Distance + (1 | Site)

Parameter	*Estimate*	*SE*	*95% CI*
Intercept	3.90	0.18	3.55 - 4.25
Edge	-0.11	0.16	-0.43 - 0.22
Core	0.08	0.16	-0.24 - 0.40
shape	6.39	2.20	2.96 - 11.47

Understory abundance ~ 1 + (1 | Site)

Parameter	*Estimate*	*SE*	*95% CI*
Intercept	3.89	0.15	3.59 - 4.17
shape	6.44	2.17	3.05 - 11.45

Table S23. Model summary tables for GLMM analyses used to explain differences in ***ground spider abundance*** across the chronosequence. We present model estimates and associated standard errors and 95% credible intervals for each fixed parameter and the negative binomial shape parameter. In all models, we specified *Age M-Buffer* as the intercept (Cohort: *Age M*, *Age 1*, *Age 3*, *Age 8;* Distance: *Buffer*, *Edge*, *Core*).

Ground abundance ~ Cohort*Distance + (1 | Site)

Parameter	*Estimate*	*SE*	*95% CI*
Intercept	1.59	0.32	0.95 - 2.21
Age1	-0.19	0.45	-1.06 - 0.72
Age3	-0.11	0.45	-0.98 - 0.79
Age8	-0.72	0.49	-1.66 - 0.26
Edge	0.10	0.30	-0.49 - 0.69
Core	0.37	0.30	-0.21 - 0.95
Age1:Edge	0.11	0.44	-0.75 - 0.96
Age3:Edge	-0.19	0.44	-1.04 - 0.68
Age8:Edge	0.14	0.50	-0.83 - 1.11
Age1:Core	-0.18	0.45	-1.06 - 0.71
Age3:Core	0.39	0.43	-0.46 - 1.24
Age8:Core	-0.56	0.50	-1.54 - 0.43
shape	1.57	0.31	1.06 - 2.26

Ground abundance ~ Cohort + Distance + (1 | Site)

Parameter	*Estimate*	*SE*	*95% CI*
Intercept	1.58	0.31	0.97 - 2.18
Age1	-0.20	0.40	-0.99 - 0.60
Age3	-0.01	0.40	-0.80 - 0.79
Age8	-0.82	0.44	-1.67 - 0.08
Edge	0.10	0.20	-0.30 - 0.50
Core	0.36	0.21	-0.04 - 0.77
shape	1.55	0.30	1.05 - 2.21

Ground abundance ~ Cohort + (1 | Site)

Parameter	*Estimate*	*SE*	*95% CI*
Intercept	1.73	0.30	1.12 - 2.31
Age1	-0.21	0.42	-1.04 - 0.63
Age3	0.01	0.42	-0.81 - 0.85
Age8	-0.82	0.46	-1.71 - 0.13
shape	1.51	0.28	1.04 - 2.13

Ground abundance ~ Distance + (1 | Site)

Parameter	*Estimate*	*SE*	*95% CI*
Intercept	1.37	0.22	0.93 - 1.80
Edge	0.09	0.20	-0.31 - 0.49
Core	0.36	0.21	-0.04 - 0.76
shape	1.55	0.30	1.05 - 2.23

Ground abundance ~ 1 + (1 | Site)

Parameter	*Estimate*	*SE*	*95% CI*
Intercept	1.52	0.20	1.12 - 1.90
shape	1.52	0.28	1.05 - 2.14

Table S24. Pairwise comparisons of estimated marginal means between factor levels in the optimal ***understory spider abundance*** model: *Abundance ~ Cohort*Distance + (1 | Site)*. We concluded that factor levels were meaningfully different if the 95% highest posterior density (HPD) interval of the median point estimate calculated from our comparisons did not overlap with zero (indicated in bold). Results are given on the log scale and with a HPD interval probability of 0.95.

Comparison			Lower HPD	Upper HPD	Estimate
Age M-Buffer	---	Age 1-Buffer	-0.542	0.936	0.188
Age M-Buffer	---	Age 3-Buffer	-0.161	1.329	0.596
Age M-Buffer	---	Age 8-Buffer	-0.578	1.009	0.203
Age M-Buffer	---	Age M-Edge	-0.443	0.286	-0.080
**Age M-Buffer**	**---**	**Age 1-Edge**	**0.386**	**1.947**	**1.155**
Age M-Buffer	---	Age 3-Edge	-0.456	1.068	0.327
Age M-Buffer	---	Age 8-Edge	-0.805	0.831	0.022
Age M-Buffer	---	Age M-Core	-0.572	0.161	-0.204
**Age M-Buffer**	**---**	**Age 1-Core**	**0.140**	**1.694**	**0.915**
Age M-Buffer	---	Age 3-Core	-0.860	0.663	-0.072
Age M-Buffer	---	Age 8-Core	-0.798	0.833	0.017
Age 1-Buffer	---	Age 3-Buffer	-0.426	1.198	0.406
Age 1-Buffer	---	Age 8-Buffer	-0.860	0.871	0.015
Age 1-Buffer	---	Age M-Edge	-1.042	0.496	-0.269
**Age 1-Buffer**	**---**	**Age 1-Edge**	**0.515**	**1.401**	**0.968**
Age 1-Buffer	---	Age 3-Edge	-0.686	0.952	0.139
Age 1-Buffer	---	Age 8-Edge	-1.025	0.724	-0.164
Age 1-Buffer	---	Age M-Core	-1.147	0.387	-0.391
**Age 1-Buffer**	**---**	**Age 1-Core**	**0.296**	**1.148**	**0.729**
Age 1-Buffer	---	Age 3-Core	-1.101	0.536	-0.260
Age 1-Buffer	---	Age 8-Core	-1.053	0.701	-0.173
Age 3-Buffer	---	Age 8-Buffer	-1.259	0.468	-0.393
Age 3-Buffer	---	Age M-Edge	-1.432	0.115	-0.675
Age 3-Buffer	---	Age 1-Edge	-0.262	1.422	0.559
Age 3-Buffer	---	Age 3-Edge	-0.697	0.153	-0.270
Age 3-Buffer	---	Age 8-Edge	-1.447	0.316	-0.573
**Age 3-Buffer**	**---**	**Age M-Core**	**-1.564**	**-0.017**	**-0.800**
Age 3-Buffer	---	Age 1-Core	-0.517	1.157	0.319
**Age 3-Buffer**	**---**	**Age 3-Core**	**-1.087**	**-0.243**	**-0.670**
Age 3-Buffer	---	Age 8-Core	-1.451	0.308	-0.579
Age 8-Buffer	---	Age M-Edge	-1.095	0.543	-0.285
**Age 8-Buffer**	**---**	**Age 1-Edge**	**0.076**	**1.855**	**0.951**
Age 8-Buffer	---	Age 3-Edge	-0.769	0.986	0.123
Age 8-Buffer	---	Age 8-Edge	-0.642	0.277	-0.181
Age 8-Buffer	---	Age M-Core	-1.218	0.416	-0.408
Age 8-Buffer	---	Age 1-Core	-0.172	1.600	0.711
Age 8-Buffer	---	Age 3-Core	-1.167	0.575	-0.277
Age 8-Buffer	---	Age 8-Core	-0.637	0.286	-0.187
**Age M-Edge**	**---**	**Age 1-Edge**	**0.434**	**2.029**	**1.235**
Age M-Edge	---	Age 3-Edge	-0.386	1.170	0.408
Age M-Edge	---	Age 8-Edge	-0.755	0.914	0.105
Age M-Edge	---	Age M-Core	-0.508	0.264	-0.123
**Age M-Edge**	**---**	**Age 1-Core**	**0.202**	**1.788**	**0.996**
Age M-Edge	---	Age 3-Core	-0.797	0.759	0.009
Age M-Edge	---	Age 8-Core	-0.751	0.916	0.097
**Age 1-Edge**	**---**	**Age 3-Edge**	**-1.695**	**-0.001**	**-0.829**
**Age 1-Edge**	**---**	**Age 8-Edge**	**-2.040**	**-0.239**	**-1.131**
**Age 1-Edge**	**---**	**Age M-Core**	**-2.161**	**-0.564**	**-1.358**
Age 1-Edge	---	Age 1-Core	-0.697	0.245	-0.241
**Age 1-Edge**	**---**	**Age 3-Core**	**-2.100**	**-0.408**	**-1.228**
**Age 1-Edge**	**---**	**Age 8-Core**	**-2.053**	**-0.248**	**-1.137**
Age 3-Edge	---	Age 8-Edge	-1.194	0.589	-0.305
Age 3-Edge	---	Age M-Core	-1.315	0.252	-0.530
Age 3-Edge	---	Age 1-Core	-0.227	1.456	0.587
Age 3-Edge	---	Age 3-Core	-0.827	0.015	-0.399
Age 3-Edge	---	Age 8-Core	-1.199	0.575	-0.310
Age 8-Edge	---	Age M-Core	-1.037	0.634	-0.227
Age 8-Edge	---	Age 1-Core	-0.018	1.779	0.893
Age 8-Edge	---	Age 3-Core	-0.970	0.801	-0.096
Age 8-Edge	---	Age 8-Core	-0.487	0.456	-0.005
**Age M-Core**	**---**	**Age 1-Core**	**0.338**	**1.916**	**1.121**
Age M-Core	---	Age 3-Core	-0.653	0.900	0.131
Age M-Core	---	Age 8-Core	-0.610	1.048	0.220
**Age 1-Core**	**---**	**Age 3-Core**	**-1.839**	**-0.160**	**-0.988**
**Age 1-Core**	**---**	**Age 8-Core**	**-1.817**	**-0.010**	**-0.897**
Age 3-Core	---	Age 8-Core	-0.796	0.976	0.091

Table S25. Model summary tables for GLMM analyses used to explain differences in ***canopy spider species richness*** across the chronosequence. We present model estimates and associated standard errors and 95% credible intervals for each fixed parameter. In all models, we specified *Age M-Buffer* as the intercept (Cohort: *Age M*, *Age 1*, *Age 3*, *Age 8;* Distance: *Buffer*, *Edge*, *Core*).

Canopy richness ~ Cohort*Distance + (1 | Site)

Parameter	*Estimate*	*SE*	*95% CI*
Intercept	1.19	0.34	0.50 - 1.84
Age1	-0.30	0.48	-1.24 - 0.65
Age3	-0.22	0.48	-1.17 - 0.71
Age8	0.89	0.47	-0.07 - 1.81
Edge	-0.28	0.31	-0.89 - 0.32
Core	-0.23	0.31	-0.84 - 0.36
Age1:Edge	0.70	0.44	-0.16 - 1.56
Age3:Edge	0.73	0.42	-0.10 - 1.56
Age8:Edge	-0.28	0.40	-1.07 - 0.51
Age1:Core	0.86	0.43	0.03 - 1.71
Age3:Core	0.52	0.43	-0.33 - 1.36
Age8:Core	0.35	0.37	-0.38 - 1.09

Canopy richness ~ Cohort + Distance + (1 | Site)

Parameter	*Estimate*	*SE*	*95% CI*
Intercept	1.08	0.33	0.43 - 1.73
Age1	0.11	0.43	-0.77 - 0.96
Age3	0.08	0.44	-0.82 - 0.93
Age8	0.86	0.45	-0.08 - 1.73
Edge	-0.06	0.17	-0.40 - 0.28
Core	0.17	0.16	-0.15 - 0.50

Canopy richness ~ Cohort + (1 | Site)

Parameter	*Estimate*	*SE*	*95% CI*
Intercept	1.13	0.31	0.50 - 1.74
Age1	0.11	0.43	-0.76 - 0.96
Age3	0.08	0.44	-0.80 - 0.93
Age8	0.86	0.45	-0.08 - 1.72

Canopy richness ~ Distance + (1 | Site)

Parameter	*Estimate*	*SE*	*95% CI*
Intercept	1.31	0.23	0.83 - 1.75
Edge	-0.06	0.17	-0.40 - 0.28
Core	0.17	0.16	-0.15 - 0.50

Canopy richness ~ 1 + (1 | Site)

Parameter	*Estimate*	*SE*	*95% CI*
Intercept	1.36	0.21	0.92 - 1.75

Table S26. Model summary tables for GLMM analyses used to explain differences in ***understory spider species richness*** across the chronosequence. We present model estimates and associated standard errors and 95% credible intervals for each fixed parameter. In all models, we specified *Age M-Buffer* as the intercept (Cohort: *Age M*, *Age 1*, *Age 3*, *Age 8;* Distance: *Buffer*, *Edge*, *Core*).

Understory richness ~ Cohort*Distance + (1 | Site)

Parameter	*Estimate*	*SE*	*95% CI*
Intercept	2.30	0.17	1.95 - 2.63
Age1	-0.33	0.26	-0.84 - 0.18
Age3	-0.70	0.28	-1.25 - -0.15
Age8	-0.23	0.28	-0.77 - 0.31
Edge	0.05	0.20	-0.34 - 0.43
Core	-0.10	0.20	-0.50 - 0.30
Age1:Edge	-0.64	0.34	-1.31 - 0.02
Age3:Edge	0.11	0.33	-0.54 - 0.76
Age8:Edge	0.05	0.32	-0.57 - 0.68
Age1:Core	-1.02	0.39	-1.80 - -0.28
Age3:Core	0.62	0.32	0.00 - 1.26
Age8:Core	0.08	0.33	-0.57 - 0.73

Understory richness ~ Cohort + Distance + (1 | Site)

Parameter	*Estimate*	*SE*	*95% CI*
Intercept	2.33	0.16	2.01 - 2.63
Age1	-0.76	0.21	-1.18 - -0.33
Age3	-0.43	0.20	-0.82 - -0.01
Age8	-0.18	0.21	-0.59 - 0.25
Edge	-0.04	0.13	-0.30 - 0.22
Core	-0.09	0.13	-0.35 - 0.17

Understory richness ~ Cohort + (1 | Site)

Parameter	*Estimate*	*SE*	*95% CI*
Intercept	2.29	0.14	2.00 - 2.55
Age1	-0.76	0.21	-1.18 - -0.33
Age3	-0.43	0.20	-0.82 - -0.02
Age8	-0.18	0.21	-0.59 - 0.25

Understory richness ~ Distance + (1 | Site)

Parameter	*Estimate*	*SE*	*95% CI*
Intercept	1.99	0.14	1.71 - 2.25
Edge	-0.04	0.13	-0.30 - 0.22
Core	-0.09	0.13	-0.35 - 0.17

Understory richness ~ 1 + (1 | Site)

Parameter	*Estimate*	*SE*	*95% CI*
Intercept	1.95	0.12	1.71 - 2.17

Table S27. Model summary tables for GLMM analyses used to explain differences in ***ground spider species richness*** across the chronosequence. We present model estimates and associated standard errors and 95% credible intervals for each fixed parameter. In all models, we specified *Age M-Buffer* as the intercept (Cohort: *Age M*, *Age 1*, *Age 3*, *Age 8;* Distance: *Buffer*, *Edge*, *Core*).

Ground richness ~ Cohort*Distance + (1 | Site)

Parameter	*Estimate*	*SE*	*95% CI*		
Intercept	0.67	0.29	0.07	-	1.20
Age1	-0.42	0.41	-1.21	-	0.40
Age3	-0.25	0.40	-1.03	-	0.57
Age8	-0.85	0.47	-1.76	-	0.08
Edge	-0.11	0.24	-0.58	-	0.36
Core	0.38	0.22	-0.05	-	0.81
Age1:Edge	0.64	0.36	-0.06	-	1.35
Age3:Edge	-0.29	0.39	-1.07	-	0.47
Age8:Edge	0.57	0.45	-0.31	-	1.45
Age1:Core	-0.20	0.36	-0.90	-	0.51
Age3:Core	-0.30	0.35	-0.99	-	0.39
Age8:Core	-0.58	0.49	-1.54	-	0.36

Ground richness ~ Cohort + Distance + (1 | Site)

Parameter	*Estimate*	*SE*	*95% CI*		
Intercept	0.68	0.28	0.09	-	1.20
Age1	-0.28	0.37	-0.99	-	0.47
Age3	-0.42	0.37	-1.13	-	0.35
Age8	-0.83	0.42	-1.62	-	0.03
Edge	0.08	0.16	-0.23	-	0.40
Core	0.21	0.16	-0.10	-	0.52

Ground richness ~ Cohort + (1 | Site)

Parameter	*Estimate*	*SE*	*95% CI*		
Intercept	0.79	0.26	0.23	-	1.27
Age1	-0.28	0.37	-0.98	-	0.48
Age3	-0.42	0.37	-1.13	-	0.34
Age8	-0.83	0.41	-1.61	-	0.03

Ground richness ~ Distance + (1 | Site)

Parameter	*Estimate*	*SE*	*95% CI*		
Intercept	0.34	0.20	-0.06	-	0.72
Edge	0.08	0.16	-0.23	-	0.40
Core	0.21	0.16	-0.10	-	0.51

Ground richness ~ 1 + (1 | Site)

Parameter	*Estimate*	*SE*	*95% CI*	
Intercept	0.44	0.17	0.08	0.77

Table S28. Pairwise comparisons of estimated marginal means between factor levels in the optimal ***understory spider species richness*** model: *Richness ~ Cohort + (1 | Site)*. We concluded that factor levels were meaningfully different if the 95% highest posterior density (HPD) interval of the median point estimate calculated from our comparisons did not overlap with zero (indicated in bold). Results are given on the log scale and with a HPD interval probability of 0.95.

Comparison			Lower HPD	Upper HPD	Estimate
**Age M**	**---**	**Age 1**	**0.336**	**1.185**	**0.760**
**Age M**	**---**	**Age 3**	**0.022**	**0.823**	**0.435**
Age M	---	Age 8	-0.236	0.605	0.186
Age 1	---	Age 3	-0.790	0.106	-0.326
**Age 1**	**---**	**Age 8**	**-1.044**	**-0.104**	**-0.576**
Age 3	---	Age 8	-0.699	0.196	-0.248

Table S29. Results of post-hoc tests assessing differences in the optimal ***canopy spider species-level community composition*** model: *Composition ~ Cohort*Distance + (1 | Site)*. No pairwise comparisons were significantly different from each other ($p > 0.05$). The significance of *Cohort*, *Distance*, and the interaction of these factors to the model, and associated likelihood ratio test statistics, are also provided. We indicate significance in bold font: *** = $p < 0.001$, ** = $p < 0.01$, * = $p < 0.05$.

Factor	LRT statistic	p-value
f(Cohort)	**257.1**	**0.041***
f(Distance)	**408.7**	**0.003****
f(Cohort*Distance)	**70.3**	**0.002****

Comparison			LRT statistic	p-value
Age 1-Core	---	Age 8-Core	80.67	0.099
Age 3-Edge	---	Age 8-Core	72.53	0.216
Age 1-Edge	---	Age 8-Core	72.37	0.216
Age M-Buffer	---	Age 8-Core	70.71	0.216
Age 8-Buffer	---	Age 1-Edge	70.07	0.216
Age 8-Buffer	---	Age 1-Core	69.08	0.216
Age 8-Buffer	---	Age 3-Edge	69	0.216
Age 3-Buffer	---	Age 8-Core	64.99	0.256
Age 3-Core	---	Age 8-Core	63.92	0.262
Age 8-Buffer	---	Age M-Edge	62.53	0.272
Age M-Edge	---	Age 8-Core	61.96	0.275
Age 1-Buffer	---	Age 8-Core	60.5	0.297
Age 8-Buffer	---	Age 3-Core	59.29	0.313
Age M-Core	---	Age 8-Core	56.26	0.408
Age 3-Buffer	---	Age 8-Buffer	56.1	0.408
Age 1-Buffer	---	Age 8-Buffer	52.72	0.532
Age 8-Buffer	---	Age 8-Core	52.31	0.534
Age 8-Edge	---	Age 1-Core	51.41	0.534
Age 8-Buffer	---	Age M-Core	50.06	0.557
Age M-Buffer	---	Age 1-Core	47.81	0.672
Age 8-Edge	---	Age 8-Core	47.39	0.682
Age 3-Edge	---	Age 8-Edge	46.81	0.682
Age M-Buffer	---	Age 3-Edge	45.36	0.726
Age M-Buffer	---	Age 1-Edge	45.03	0.726
Age M-Core	---	Age 1-Core	44.72	0.726
Age 1-Edge	---	Age 8-Edge	44.15	0.731
Age 3-Edge	---	Age M-Core	43.33	0.753
Age M-Buffer	---	Age 3-Core	42.58	0.779
Age M-Buffer	---	Age 8-Buffer	41.72	0.805
Age 3-Edge	---	Age 1-Core	40.69	0.822
Age M-Edge	---	Age 1-Core	40.45	0.822
Age 1-Core	---	Age 3-Core	39.63	0.843
Age 8-Buffer	---	Age 8-Edge	39.58	0.843
Age 1-Edge	---	Age M-Core	39.160	0.843
Age 3-Buffer	---	Age 1-Core	39.070	0.843
Age 1-Edge	---	Age 1-Core	39.050	0.843
Age 1-Edge	---	Age 3-Core	38.590	0.843
Age M-Edge	---	Age 3-Edge	38.020	0.843
Age M-Buffer	---	Age M-Edge	37.860	0.843
Age M-Edge	---	Age 1-Edge	37.670	0.843
Age 8-Edge	---	Age 3-Core	37.360	0.843
Age M-Edge	---	Age 8-Edge	37.340	0.843
Age 1-Edge	---	Age 3-Edge	37.060	0.843
Age M-Buffer	---	Age 8-Edge	35.420	0.843
Age 3-Buffer	---	Age 3-Edge	34.890	0.843
Age M-Buffer	---	Age M-Core	34.460	0.843
Age 3-Buffer	---	Age 8-Edge	34.260	0.843
Age 1-Buffer	---	Age 8-Edge	34.090	0.843
Age 3-Buffer	---	Age 3-Core	33.850	0.843
Age 1-Buffer	---	Age 1-Core	33.520	0.843
Age 1-Buffer	---	Age 1-Edge	33.510	0.843
Age M-Core	---	Age 3-Core	32.640	0.843
Age 3-Buffer	---	Age 1-Edge	30.740	0.843
Age 3-Buffer	---	Age M-Core	30.600	0.843
Age M-Buffer	---	Age 3-Buffer	30.140	0.843
Age M-Edge	---	Age 3-Core	29.690	0.843
Age 3-Edge	---	Age 3-Core	29.550	0.843
Age 3-Buffer	---	Age M-Edge	29.110	0.843
Age 1-Buffer	---	Age M-Core	27.830	0.843
Age M-Buffer	---	Age 1-Buffer	26.820	0.843
Age 8-Edge	---	Age M-Core	26.580	0.843
Age 1-Buffer	---	Age 3-Edge	25.530	0.843
Age 1-Buffer	---	Age 3-Core	24.470	0.843
Age 1-Buffer	---	Age M-Edge	23.570	0.843
Age 1-Buffer	---	Age 3-Buffer	21.130	0.843
Age M-Edge	---	Age M-Core	19.570	0.843

Table S30. Results of post-hoc tests assessing differences in the optimal ***understory spider species-level community composition*** model: *Composition ~ Cohort*Distance + (1 | Site)*. No pairwise comparisons were significantly different from each other ($p > 0.05$). The significance of *Cohort*, *Distance*, and the interaction of these factors to the model, and associated likelihood ratio test statistics, are also provided. We indicate significance in bold font: *** = $p < 0.001$, ** = $p < 0.01$, * = $p < 0.05$.

Factor	LRT statistic	p-value
f(Cohort)	**246.9**	**<0.001*** **
f(Distance)	**532.1**	**<0.001*** **
f(Cohort*Distance)	**166.4**	**<0.001*** **

Comparison			LRT statistic	p-value	Comparison			LRT statistic	p-value
Age M-Edge	---	Age 3-Core	118.52	0.062	Age 8-Buffer	---	Age 1-Edge	67.310	0.577
Age M-Edge	---	Age 3-Edge	109.38	0.105	Age 1-Buffer	---	Age 8-Core	66.470	0.578
Age M-Buffer	---	Age 3-Core	107.9	0.108	Age 1-Buffer	---	Age 1-Core	61.300	0.729
Age M-Edge	---	Age 1-Edge	104.72	0.123	Age 3-Buffer	---	Age 3-Edge	57.930	0.821
Age M-Core	---	Age 3-Core	103.8	0.125	Age 1-Buffer	---	Age 1-Edge	57.870	0.821
Age M-Edge	---	Age 1-Core	102.77	0.125	Age 3-Buffer	---	Age 8-Edge	57.250	0.824
Age 8-Edge	---	Age 3-Core	100.32	0.139	Age 1-Buffer	---	Age M-Core	56.840	0.827
Age 8-Buffer	---	Age 3-Core	98.17	0.149	Age 3-Buffer	---	Age 8-Buffer	55.090	0.844
Age 1-Buffer	---	Age 3-Core	96.61	0.153	Age M-Buffer	---	Age 8-Core	55.020	0.844
Age 3-Core	---	Age 8-Core	95.53	0.157	Age 8-Edge	---	Age M-Core	54.170	0.849
Age 3-Buffer	---	Age M-Edge	94.42	0.159	Age 3-Buffer	---	Age 1-Edge	54.110	0.849
Age 3-Edge	---	Age M-Core	89.98	0.212	Age 1-Buffer	---	Age 8-Edge	53.830	0.849
Age M-Buffer	---	Age 1-Core	87.34	0.244	Age M-Buffer	---	Age 8-Edge	53.120	0.849
Age M-Buffer	---	Age 3-Edge	86.87	0.247	Age M-Edge	---	Age 8-Core	50.340	0.873
Age M-Core	---	Age 1-Core	85.23	0.261	Age 3-Edge	---	Age 1-Core	50.210	0.873
Age M-Buffer	---	Age 1-Edge	85.12	0.261	Age 1-Edge	---	Age 3-Edge	49.420	0.875
Age 3-Edge	---	Age 8-Edge	84.78	0.261	Age 1-Buffer	---	Age 3-Buffer	47.480	0.907
Age 1-Edge	---	Age M-Core	84.44	0.261	Age M-Buffer	---	Age 1-Buffer	47.240	0.907
Age 3-Buffer	---	Age 3-Core	84.01	0.261	Age M-Core	---	Age 8-Core	46.520	0.907
Age 1-Core	---	Age 3-Core	81.32	0.292	Age 3-Edge	---	Age 3-Core	46.120	0.907
Age 1-Edge	---	Age 3-Core	80.03	0.318	Age 3-Buffer	---	Age 1-Core	45.240	0.907
Age 3-Buffer	---	Age M-Core	78.86	0.339	Age M-Buffer	---	Age 8-Buffer	44.970	0.907
Age M-Buffer	---	Age 3-Buffer	74.54	0.442	Age M-Edge	---	Age 8-Edge	43.910	0.907
Age 8-Buffer	---	Age 3-Edge	73.43	0.465	Age 1-Buffer	---	Age 8-Buffer	43.640	0.907
Age 3-Edge	---	Age 8-Core	72.46	0.488	Age 8-Buffer	---	Age M-Edge	42.470	0.907
Age 1-Edge	---	Age 8-Edge	72.38	0.488	Age M-Buffer	---	Age M-Edge	41.420	0.907
Age 1-Edge	---	Age 8-Core	71.09	0.514	Age M-Edge	---	Age M-Core	38.700	0.907
Age 1-Core	---	Age 8-Core	71.06	0.514	Age 8-Buffer	---	Age M-Core	38.540	0.907
Age 3-Buffer	---	Age 8-Core	70.39	0.521	Age 8-Buffer	---	Age 8-Edge	35.920	0.907
Age 1-Buffer	---	Age M-Edge	68.37	0.577	Age M-Buffer	---	Age M-Core	34.850	0.907
Age 1-Buffer	---	Age 3-Edge	68.32	0.577	Age 8-Edge	---	Age 8-Core	32.370	0.907
Age 8-Edge	---	Age 1-Core	68.09	0.577	Age 8-Buffer	---	Age 8-Core	30.250	0.907
Age 8-Buffer	---	Age 1-Core	67.45	0.577	Age 1-Edge	---	Age 1-Core	17.910	0.907

Table S31. Results of post-hoc tests assessing differences in the optimal ***ground spider species-level community composition*** model: *Composition ~ Cohort*Distance + (1 | Site)*. No pairwise comparisons were significantly different from each other ($p > 0.05$). The significance of *Cohort*, *Distance*, and the interaction of these factors to the model, and associated likelihood ratio test statistics, are also provided. We indicate significance in bold font: *** = $p < 0.001$, ** = $p < 0.01$, * = $p < 0.05$.

Factor	LRT statistic	p-value
f(Cohort)	191.9	0.209
f(Distance)	**335.0**	**0.002****
f(Distance*Cohort)	**79.5**	**0.002****

Comparison			LRT statistic	p-value	Comparison			LRT statistic	p-value
Age M-Buffer	---	Age 1-Edge	73.16	0.121	Age M-Buffer	---	Age M-Core	35.520	0.904
Age 1-Edge	---	Age M-Core	71.88	0.127	Age 1-Edge	---	Age 8-Core	34.990	0.904
Age 3-Buffer	---	Age M-Core	64.41	0.24	Age M-Buffer	---	Age M-Edge	33.120	0.932
Age 3-Edge	---	Age M-Core	58.52	0.414	Age 8-Edge	---	Age 1-Core	32.660	0.932
Age M-Core	---	Age 3-Core	56.73	0.469	Age 8-Buffer	---	Age 1-Core	32.530	0.932
Age 1-Edge	---	Age 3-Edge	55.42	0.504	Age 1-Buffer	---	Age 3-Buffer	31.900	0.932
Age 1-Edge	---	Age 3-Core	54.8	0.524	Age M-Edge	---	Age M-Core	31.790	0.932
Age 3-Buffer	---	Age 1-Edge	54.54	0.524	Age 1-Buffer	---	Age 3-Core	31.420	0.932
Age 1-Buffer	---	Age 1-Edge	52.53	0.591	Age 1-Buffer	---	Age 8-Edge	30.930	0.932
Age M-Edge	---	Age 1-Edge	52.3	0.593	Age M-Buffer	---	Age 8-Core	30.740	0.932
Age M-Buffer	---	Age 3-Buffer	50.31	0.664	Age 3-Edge	---	Age 8-Edge	30.730	0.932
Age 3-Buffer	---	Age 1-Core	48.81	0.718	Age 1-Buffer	---	Age 1-Core	29.850	0.932
Age M-Buffer	---	Age 1-Core	48.27	0.725	Age 3-Buffer	---	Age 3-Core	29.820	0.932
Age M-Core	---	Age 1-Core	47.7	0.735	Age 1-Core	---	Age 8-Core	29.340	0.932
Age 8-Edge	---	Age M-Core	47.7	0.735	Age 3-Core	---	Age 8-Core	29.310	0.932
Age 1-Core	---	Age 3-Core	46.76	0.735	Age 1-Buffer	---	Age 3-Edge	29.230	0.932
Age M-Buffer	---	Age 3-Core	46.65	0.735	Age 1-Buffer	---	Age M-Edge	28.800	0.932
Age 8-Buffer	---	Age 1-Edge	44.77	0.787	Age 3-Buffer	---	Age 3-Edge	28.720	0.932
Age M-Edge	---	Age 3-Core	44.34	0.797	Age M-Edge	---	Age 8-Edge	28.700	0.932
Age 3-Buffer	---	Age M-Edge	44.33	0.797	Age 8-Buffer	---	Age M-Edge	28.640	0.932
Age 1-Buffer	---	Age M-Core	43.76	0.797	Age 3-Buffer	---	Age 8-Core	27.480	0.932
Age 8-Buffer	---	Age M-Core	43.43	0.797	Age 8-Buffer	---	Age 3-Core	26.780	0.932
Age M-Edge	---	Age 3-Edge	42.83	0.797	Age 3-Edge	---	Age 3-Core	26.340	0.932
Age 1-Edge	---	Age 1-Core	42.72	0.797	Age 3-Buffer	---	Age 8-Buffer	25.120	0.932
Age M-Buffer	---	Age 8-Edge	42.51	0.797	Age M-Buffer	---	Age 1-Buffer	24.780	0.932
Age M-Core	---	Age 8-Core	41.63	0.797	Age 8-Buffer	---	Age 3-Edge	24.620	0.932
Age M-Buffer	---	Age 3-Edge	41.47	0.797	Age M-Edge	---	Age 8-Core	24.410	0.932
Age 3-Edge	---	Age 1-Core	40.72	0.797	Age 1-Buffer	---	Age 8-Buffer	22.040	0.932
Age 3-Buffer	---	Age 8-Edge	39.55	0.826	Age 8-Edge	---	Age 8-Core	21.530	0.932
Age 1-Edge	---	Age 8-Edge	39.24	0.826	Age 3-Edge	---	Age 8-Core	21.430	0.932
Age M-Edge	---	Age 1-Core	37.9	0.863	Age 1-Buffer	---	Age 8-Core	20.580	0.932
Age M-Buffer	---	Age 8-Buffer	36.51	0.883	Age 8-Buffer	---	Age 8-Edge	19.130	0.932
Age 8-Edge	---	Age 3-Core	35.66	0.904	Age 8-Buffer	---	Age 8-Core	14.230	0.932

Table S32. List of spider morphospecies found in the *canopy*, *understory*, *and ground microhabitats*. Only adult spiders were identified to morphospecies. For each morphospecies, we indicate whether it was found in the canopy, understory, or ground microhabitat. Boxes that are highlighted in orange indicate that morphospecies abundance in that microhabitat varied significantly across the chronosequence, as determined by univariate analyses from our species-level community composition analyses.

Family	Morphospecies	Canopy	Understory	Ground
Anapidae	Anapidae sp. 1	X		
	Anapidae sp. 2	X		
Araneidae	Araneidae sp. 1		X	
	Araneidae sp. 2		X	
	Araneidae sp. 3		X	
	Araneidae sp. 4		X	
	Araneidae sp. 5		X	
	Araneidae sp. 6		X	
	Araneidae sp. 7		X	
	Araneidae sp. 8		X	
	Araneidae sp. 9		X	
	Araneidae sp. 10		X	
	Araneidae sp. 11	X	X	
	Araneidae sp. 12		X	
	Araneidae sp. 13	X	X	
	Araneidae sp. 14	X	X	
	Araneidae sp. 15	X	X	
	Araneidae sp. 16	X		
	Araneidae sp. 17	X		
	Araneidae sp. 18	X		
	Araneidae sp. 19	X		
	Araneidae sp. 20		X	
	Araneidae sp. 21		X	
	Araneidae sp. 22	X	X	
	Araneidae sp. 23	X	X	
	Araneidae sp. 24	X	X	
	Araneidae sp. 25		X	
	Araneidae sp. 26	X		
	Gasteracantha sp. 1	X	X	
	Gasteracantha sp. 2	X	X	
	Gasteracantha sp. 3		X	
	Gasteracantha sp. 4	X	X	
	Gasteracantha sp. 5		X	
	Nephila pilipes		X	
Clubionidae	Clubiondae sp. 1		X	
	Clubiondae sp. 2	X		
	Clubiondae sp. 3	X		

**** Continued on next page**

Corinnidae	Corinnidae sp. 1		X	
	Corinnidae sp. 2	X		
	Corinnidae sp. 3	X		
	Corinnidae sp. 4	X		
Ctenidae	Ctenidae sp. 1			X
Gnaphosidae	Gnaphosidae sp. 1			X
Linyphiidae	Linyphiidae sp. 1	X		
	Linyphiidae sp. 2			X
	Linyphiidae sp. 3	X		
	Linyphiidae sp. 4			X
	Linyphiidae sp. 5	X		
	Linyphiidae sp. 6			X
	Linyphiidae sp. 7			X
	Linyphiidae sp. 8			X
	Linyphiidae sp. 9			X
	Linyphiidae sp. 10			X
	Linyphiidae sp. 11	X		
	Linyphiidae sp. 12	X		
	Linyphiidae sp. 13	X		
	Linyphiidae sp. 14	X		X
	Linyphiidae sp. 15			X
	Linyphiidae sp. 16	X		
	Linyphiidae sp. 17	X		X
	Linyphiidae sp. 18			X
	Linyphiidae sp. 19	X		X
Liocranidae	Liocranidae sp. 1			X
	Liocranidae sp. 2			X
Lycosidae	Lycosidae sp. 1			X
	Lycosidae sp. 2			X
	Lycosidae sp. 3			X
	Lycosidae sp. 4			X
	Lycosidae sp. 5			X
	Lycosidae sp. 6			X
	Lycosidae sp. 7			X
	Lycosidae sp. 8			X
	Lycosidae sp. 9			X
	Lycosidae sp. 10			X
	Lycosidae sp. 11			X
	Lycosidae sp. 12			X
	Lycosidae sp. 13			X
	Lycosidae sp. 14			X
	Lycosidae sp. 15			X
	Lycosidae sp. 16			X
	Lycosidae sp. 17			X
	Lycosidae sp. 18			X
	Lycosidae sp. 19			X
	Lycosidae sp. 20			X
	Lycosidae sp. 21			X
	Lycosidae sp. 22			X
	Lycosidae sp. 23			X
	Lycosidae sp. 24			X

**** Continued on next page**

Mysmenidae	Mysmenidae sp. 1	X	X	
	Mysmenidae sp. 2	X		
	Mysmenidae sp. 3	X		
Nesticidae	Nesticidae sp. 1	X		
Oonopidae	Oonopidae sp. 1	X		
	Oonopidae sp. 2	X		
	Oonopidae sp. 3	X		
	Oonopidae sp. 4	X		
	Oonopidae sp. 5	X		
	Oonopidae sp. 6	X		X
	Oonopidae sp. 7			X
	Oonopidae sp. 8	X		X
	Oonopidae sp. 9			X
	Oonopidae sp. 10	X		X
	Oonopidae sp. 11	X		
	Oonopidae sp. 12	X		
	Oonopidae sp. 13	X		
	Oonopidae sp. 14	X		X
	Oonopidae sp. 15	X		
	Oonopidae sp. 16	X		X
	Oonopidae sp. 17			X
	Oonopidae sp. 18			X
	Ischnothyreus sp. 1			X
Oxyopidae	Oxyopidae sp. 1		X	
	Oxyopidae sp. 2		X	
	Oxyopidae sp. 3		X	
	Oxyopidae sp. 4		X	
	Oxyopidae sp. 5		X	
	Oxyopidae sp. 6			X
Pholcidae	Pholcidae sp. 1		X	
Salticidae	Salticidae sp. 1	X	X	
	Salticidae sp. 2	X		
	Salticidae sp. 3	X		
	Salticidae sp. 4	X		
	Salticidae sp. 5	X		
	Salticidae sp. 6		X	
	Salticidae sp. 7	X		
	Salticidae sp. 8			X
	Salticidae sp. 9			X
	Salticidae sp. 10			X
	Salticidae sp. 11			X
	Salticidae sp. 12			X
	Salticidae sp. 13		X	
	Salticidae sp. 14		X	
	Salticidae sp. 15		X	
	Salticidae sp. 16		X	
	Salticidae sp. 17		X	
	Salticidae sp. 18		X	

**** Continued on next page**

Symphytognathidae	Symphytognathidae sp. 1	X		
	Symphytognathidae sp. 2	X		
Tetragnathidae	Opadometa sp. 1	X	X	
	Opadometa sp. 2		X	
	Tetragnatha sp. 1	X	X	
	Tetragnathidae sp. 1	X	X	
	Tetragnathidae sp. 2		X	
	Tetragnathidae sp. 3		X	
	Tetragnathidae sp. 4		X	
	Tetragnathidae sp. 5	X		
	Tetragnathidae sp. 6	X		
	Tylorida sp. 1	X	X	
	Tylorida sp. 2		X	
	Tylorida sp. 3	X	X	
	Tylorida sp. 4	X	X	
Theridiidae	Argyrodes sp. 1		X	
	Argyrodes sp. 2	X	X	
	Ariamnes sp. 1	X		
	Ariamnes sp. 2	X		
	Rhomphaea sp. 1	X		
	Rhomphaea sp. 2	X		
	Rhomphaea sp. 3	X		
	Theridiidae sp. 1		X	
	Theridiidae sp. 2		X	
	Theridiidae sp. 3		X	
	Theridiidae sp. 4		X	
	Theridiidae sp. 5	X	X	
	Theridiidae sp. 6		X	
	Theridiidae sp. 7		X	
	Theridiidae sp. 8	X	X	
	Theridiidae sp. 9	X		
	Theridiidae sp. 10	X		
	Theridiidae sp. 11	X		
	Theridiidae sp. 12	X		
	Theridiidae sp. 13	X		
	Theridiidae sp. 14	X		
	Theridiidae sp. 15	X		
	Theridiidae sp. 16		X	
	Theridiidae sp. 17	X		X
	Theridiidae sp. 18	X		
	Theridiidae sp. 19		X	
	Theridiidae sp. 20		X	
	Theridiidae sp. 21			X
	Theridiidae sp. 22			X
	Theridiidae sp. 23			X
	Theridiidae sp. 24			X
	Theridiidae sp. 25			X
	Theridiidae sp. 26	X	X	

**** Continued on next page**

	Theridiidae sp. 27	X		
	Theridiidae sp. 28		X	
	Theridiidae sp. 29	X		
	Theridiidae sp. 30		X	
	Theridiidae sp. 31	X		
	Theridiidae sp. 32	X		
	Theridiidae sp. 33	X		
	Theridiidae sp. 34	X		
	Theridiidae sp. 35	X		
	Theridiidae sp. 36			X
	Theridiidae sp. 37			X
	Theridiidae sp. 38			X
	Theridiidae sp. 39			X
	Theridiidae sp. 40			X
	Theridiidae sp. 41	X		
Theridiosomatidae	Theridiosomatidae sp. 1	X		
	Theridiosomatidae sp. 2		X	
	Theridiosomatidae sp. 3		X	
Thomisidae	Amyciaea sp. 1	X		
	Amyciaea sp. 2	X		
	Thomisidae sp. 1		X	
	Thomisidae sp. 2	X		
	Thomisidae sp. 3	X		
Trachelidae	Trachelidae sp. 1			X
Uloboridae	Uloboridae sp. 1		X	
	Uloboridae sp. 2		X	
	Uloboridae sp. 3	X	X	
	Uloboridae sp. 4		X	
	Uloboridae sp. 5		X	
	Uloboridae sp. 6		X	
Unknown	Unknown sp. 1			X
Zodariidae	Zodariidae sp. 1			X
	Zodariidae sp. 2			X
	Zodariidae sp. 3			X
	Zodariidae sp. 4			X

Section S1. Additional details on modelling.

Bayesian regression models (GLMMs). We used GLMMs to determine an effect of *Cohort* and *Distance* on canopy openness, variation in openness, vegetation height, soil temperature, total arthropod abundance, spider abundance, and spider species richness. We ran all GLMMs for 50,000 iterations using four chains and a thinning rate of 2. We discarded the first 4000 iterations as warmup / burn-in samples, and controlled the behaviour of the NUTS algorithm to decrease the number of divergent transitions (*adapt_delta = 0.95* or, when necessary, *0.99*). For our canopy openness analysis, we fitted *normal(0,1)* priors on model intercepts, *normal(0,5)* priors on fixed effects, and *gamma(0.01,0.01)* priors on beta-binomial phi parameters. For all other analyses, we fitted *normal(0,10)* priors on model intercepts, *normal(0,1)* priors on fixed effects, and *normal(0,1)* priors on the standard deviation of random effects. In addition to these, we fitted *normal(0,1)* priors on the standard deviation of splines in our soil temperature analysis, and *gamma(0.01,0.01)* priors on negative binomial shape parameters in our total arthropod abundance and spider abundance analyses.

We determined that mixing was good by inspecting MCMC trace plots and ensuring that Rhat values were <1.1, the ratio of effect sample size to total sample size was >0.1, and no autocorrelation was present within the MCMC chains (Muth et al., 2018). No issues were detected. We validated models by verifying that no patterns were present when Pearson residuals were plotted against fitted values, included covariates, and random effect levels. We then used posterior predictive checks to ensure that attributes of data that were simulated from each model accurately reflected the real dataset from which each model was generated (Gabry et al., 2019). Model validation and posterior predictive checks required *bayesplot* (Gabry et al., 2019) and *tidybayes* (Kay and Mastny, 2020).

Bayesian generalised linear latent variable model (GLLVM). We used a GLLVM to determine an effect of *Cohort* and *Distance* on ground cover. We ran the model for 50,000 iterations using one chain (Hui & Blanchard, 2020) and a thinning rate of 10. We discarded the first 8000 iterations as warmup / burn-in samples. We fitted a normal prior with mean zero and variance 1 on all column-specific intercepts, a normal prior with mean zero and variance 10 on the latent variable coefficients, and a half-Cauchy prior with mean zero and variance 5 on the standard deviation of the random effects normal distribution. We determined that mixing was good by inspecting MCMC trace plots. We validated our GLLVM by plotting Dunn-Smyth residuals against linear predictors, row indices, and column indices and ensuring no patterns were present. We also visually inspected Q-Q plots to ensure that a normal distribution was present.

Multivariate generalised linear models (mGLMs). We used mGLMs to determine an effect of *Cohort* and *Distance* on arthropod order-level composition and spider species-level composition. We validated models by verifying that no patterns were present when Dunn-Smyth residuals were plotted against fitted values and included covariates.

References

Brownrigg, R. 2018. maps: Draw Geographical Maps. R package version 3.5.1.

Environmental Systems Research Institute (ESRI). 2017. ArcGIS Desktop: Release 10.5.1

Gabry, J., Simpson, D., Vehtari, A., Betancourt, M., Gelman, A., 2019. Visualization in Bayesian workflow. J. R. Stat. Soc. Ser. A Stat. Soc. 182, 389–402. https://doi.org/10.1111/rssa.12378

Hui, F.K.C., & Blanchard, W. 2020. boral: Bayesian Ordination and Regression AnaLysis. R package version 3.5.1.

Kay, M., & Mastny, T. 2020. tidybayes: Tidy Data and 'Geoms' for Bayesian Models. R package version 3.5.1.

Muth, C., Oravecz, Z., Gabry, J., 2018. User-friendly Bayesian regression modeling: A tutorial with rstanarm and shinystan. Quant. Methods Psychol. 14, 99–119. https://doi.org/10.20982/tqmp.14.2.p099
